# Ancient Origin of the U2 Small Nuclear RNA Gene-Targeting Non-LTR Retrotransposons *Utopia*

**DOI:** 10.1371/journal.pone.0140084

**Published:** 2015-11-10

**Authors:** Kenji K. Kojima, Jerzy Jurka

**Affiliations:** 1 Genetic Information Research Institute, Los Altos, California, United States of America; 2 Department of Computational Biology and Medical Sciences, Graduate School of Frontier Sciences, University of Tokyo, Minato-ku, Tokyo, Japan; 3 Institute of Medical Science, University of Tokyo, Minato-ku, Tokyo, Japan; University of Muenster, GERMANY

## Abstract

Most non-long terminal repeat (non-LTR) retrotransposons encoding a restriction-like endonuclease show target-specific integration into repetitive sequences such as ribosomal RNA genes and microsatellites. However, only a few target-specific lineages of non-LTR retrotransposons are distributed widely and no lineage is found across the eukaryotic kingdoms. Here we report the most widely distributed lineage of target sequence-specific non-LTR retrotransposons, designated *Utopia*. *Utopia* is found in three supergroups of eukaryotes: Amoebozoa, SAR, and Opisthokonta. *Utopia* is inserted into a specific site of U2 small nuclear RNA genes with different strength of specificity for each family. *Utopia* families from oomycetes and wasps show strong target specificity while only a small number of *Utopia* copies from reptiles are flanked with U2 snRNA genes. Oomycete *Utopia* families contain an “archaeal” RNase H domain upstream of reverse transcriptase (RT), which likely originated from a plant RNase H gene. Analysis of *Utopia* from oomycetes indicates that multiple lineages of *Utopia* have been maintained inside of U2 genes with few copy numbers. Phylogenetic analysis of RT suggests the monophyly of *Utopia*, and it likely dates back to the early evolution of eukaryotes.

## Introduction

Transposable elements (TEs) or transposons are found from widespread eukaryotic genomes [1]. TEs are subdivided into two classes, DNA transposons and retrotransposons, and retrotransposons are further divided into two major groups, long terminal repeat (LTR) retrotransposons and non-LTR retrotransposons [2]. Non-LTR retrotransposons are considered to have diverged earlier than LTR retrotransposons and their wide distribution among eukaryotes indicates their presence in the early stages of eukaryote evolution [3].

Non-LTR retrotransposons are classified into clades based on their phylogenetic positions and protein structures [4,5]. Repbase, a comprehensive database of eukaryotic repeats, currently classifies non-LTR retrotransposons into 32 clades [1]. These clades are further grouped to 8 “groups” (*CRE*, *R2*, *Dualen*, *L1*, *RTE*, *I*, *CR1*, *Penelope*) [6,7]. Several clades of non-LTR retrotransposons show highly target sequence-specific integration into a certain type of repetitive sequences. Their targets include ribosomal RNA genes, spliced leader exons, tRNA genes, transposons, microsatellites and telomeric repeats [8][9][10–14][15]. Targeting multicopy sequences is considered a symbiotic survival strategy for TEs since integration into a single copy gene is more harmful than into a multicopy gene. In fact, many genetic diseases and cancers are caused by TE insertions into genes [16]. Although target sequence-specific TE families are also found in some DNA transposons [17], the majority of reported target-specific TE families belong to three groups of non-LTR retrotransposons: early-branched, *Tx1* clade, and *R1* clade [12]. Target-specific non-LTR retrotransposons belonging to the *Tx1* clade and the *R1* clade encode an endonuclease derived from cellular apurinic endonuclease [18], whereas other target-specific retrotransposons encode an endonuclease whose conserved motif is similar to PD-(D/E)xK-type restriction endonucleases [19]. Non-LTR retrotransposons encoding restriction-like endonuclease (RLE) have deeper origins compared to apurinic-like endonuclease (APE)-encoding retrotransposons based on the reverse transcriptase (RT) phylogeny [4]. Besides, the phylogenetic position of *Dualen*, a group of non-LTR retrotransposon that encodes both APE and RLE, indicates that APE-encoding non-LTR retrotransposons originated through the replacement of RLE by APE [7].

RLE-encoding retrotransposons are classified into 5 clades (when excluding *Dualen*) based on the RT phylogeny and protein domain structure [4,5,12,13]. Four clades (*CRE*, *R2*, *R4*, *NeSL*) are composed by mostly target-specific retrotransposons. The other clade, *HERO*, contains many non-specific retrotransposons, but some families show target specificity for microsatellites [20]. Relatively common target specificity in non-LTR retrotransposons encoding RLE allows us to speculate that during the early stages of their evolution, non-LTR retrotransposons were inserted in a target sequence-specific manner. This speculation is reasonable since the probability of gene disruption by random integration depends on gene density, and target specificity is more advantageous in the smaller genomes of unicellular eukaryotes than in the larger ones of multicellular eukaryotes. However, most families of non-LTR retrotransposons showing the same target specificity are distributed very narrowly. R2 is the most widely distributed family of target-specific non-LTR retrotransposons and it targets a specific site of 28S ribosomal RNA genes. R2 has been reported in 6 animal phyla: Arthropoda, Chordata, Echinodermata, Platyhelminthes, Nematoda, and Cnidaria [12,21,22][23]. The origin of the target specificity of R2 can be traced back some ~850 million years ago, well after the birth of eukaryotes [12,21,22].

Recent phylogenetic analysis of eukaryotes revealed “five supergroups” in eukaryotes: Excavata, Amoebozoa, Opisthokonta, SAR, and Archaeplastida [24]. Opithokonta includes animals and fungi in addition to several unicellular eukaryotic lineages. SAR is constituted by three groups, Stramenopiles, Alveolata, and Rhizaria. Stramenopiles includes oomycetes. The origin of these five supergroups is not clear, but they should date back to the early stage of eukaryotes.

We here report U2 small nuclear RNA (snRNA) gene-specific non-LTR retrotransposon families distributed among three eukaryotic supergroups: Amoebozoa, SAR and Opisthokonta. It indicates that these target-specific non-LTR retrotransposon families can be traced back to the period prior to the divergence of major eukaryotic supergroups.

## Results and Discussions

### The distribution of *Utopia*, U2 snRNA gene-associated non-LTR retrotransposon families

**Fig 1:**
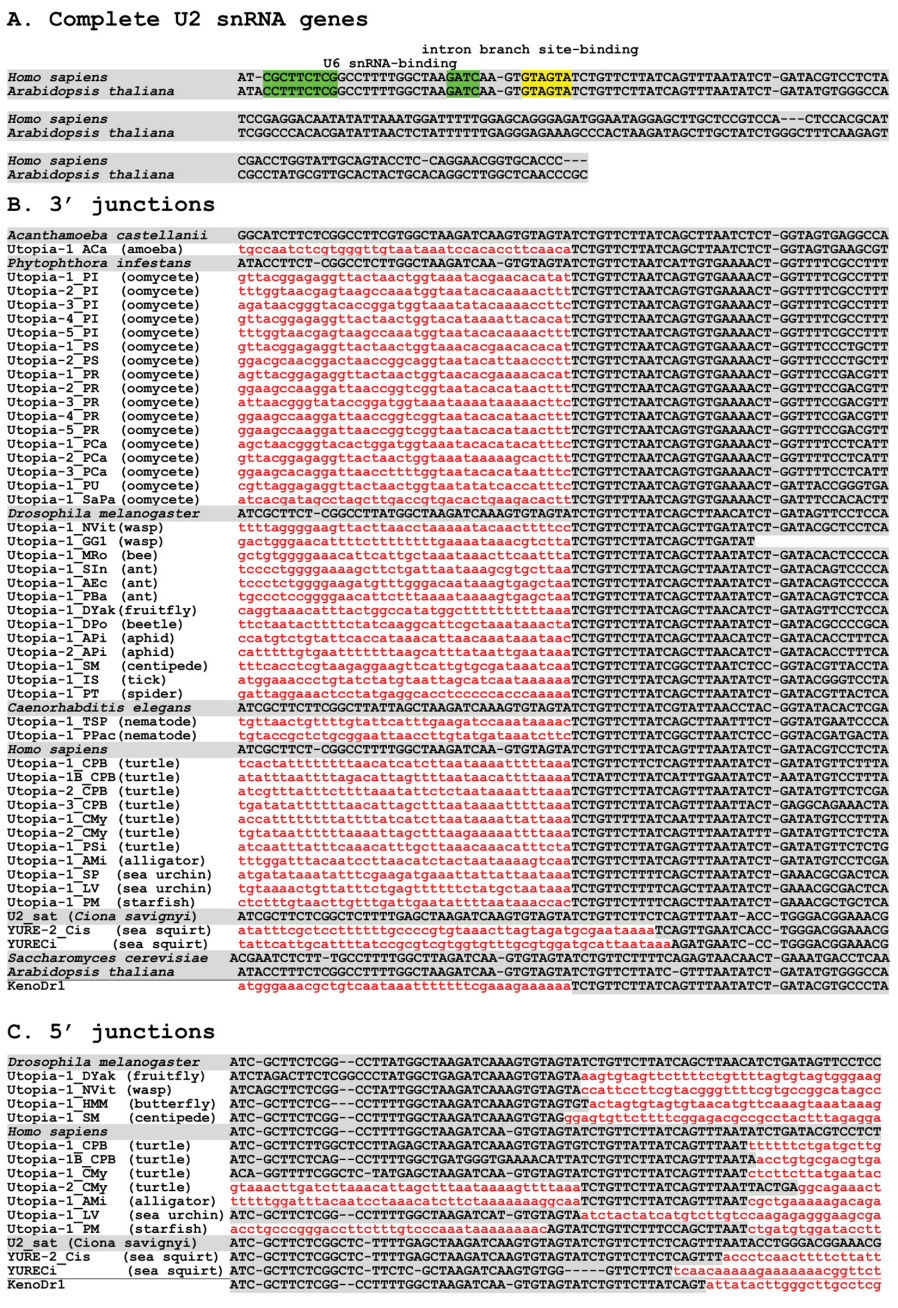
Junction sequences of *Utopia* elements and U2 snRNA genes. *Utopia* sequences are in lowercase and red-colored while U2 gene sequences are in uppercase and shaded. *Utopia* sequences are either the consensus or a representative flanked with U2 genes. (A) Complete U2 snRNA genes for human and *Arabidopsis thaliana*. These two genes are shown because they are annotated as full-length and represent the diversity of 3’ regions. Functionally important sequences are highlighted: U6 snRNA-binding, green; intron branch site-binding, yellow. (B) Comparison of 3’ junctions. (C) Comparison of 5’ junctions.

During our screening of new transposable elements and re-classification of reported transposable elements, we found three distinct non-LTR retrotransposon families that are associated with U2 snRNA genes. A non-LTR retrotransposon family from the amoeba *Acanthamoeba castellanii*, *NeSL-1_ACa* was reported to be U2 snRNA gene-specific [25]. We found one non-LTR retrotransposon family from the jewel wasp *Nasonia vitripennis* (*Utopia-1_NVit*) is associated with fragment of U2 snRNA genes. Besides, we realized that *R2I-1_PI* to *R2I-4_PI* from the oomycete *Phytophthora infestans* [26] are integrated into U2 snRNA genes at the same site (Fig 1). Using these non-LTR retrotransposons as queries, BLAST search against the reported genomes revealed related non-LTR retrotransposons from oomycetes, arthropods, nematodes, sea urchins, starfish and reptiles (Fig 1, Table 1 and S1 Table). We name them *Utopia* (U TwO snRNA gene Preferentially Inserting-or-Associating element) as most of them are flanked by U2 snRNA genes. To avoid the confusion of their target sequences, we renamed *NeSL-1_ACa* as *Utopia-1_ACa*, and *R2I-1_PI* to *R2I-4_PI* as *Utopia-1_PI* to *Utopia-4_PI*, respectively (S1 Table). It is noteworthy that *Copia-Pr1* reported by Jiang and Govers [27] is in fact a fragment of *Utopia-2_PR*.

**Table 1:** Organisms with *Utopia* families.

Classification	Organisms
Amoebozoa/Acanthamoeba	*Acanthamoeba castellanii*
SAR/Oomycetes	*Phytophthora infestans*, *Phytophthora sojae*, *Phytophthora ramorum*, *Phytophthora capsici*, *Pythium ultimum*, *Saprolegnia parasitica*, *Saprolegnia diclina*, *Hyaloperonospora arabidopsidis*, *Pseudoperonospora cubensis*
Opithokonta/Arthropoda	*Nasonia vitripennis*, *Ganaspis sp*. *G1*, *Lasioglossum albipes*, *Megachile rotundata*, *Solenopsis invicta*, *Acromyrmex echinatior*, *Atta cephalotes*, *Pogonomyrmex barbatus*, *Harpegnathos saltator*, *Camponotus floridanus*, *Drosophila yakuba*, *Heliconius melpomene*, *Chrysopa pallens*, *Dendroctonus ponderosae*, *Agrilus planipennis*, *Acyrthosiphon pisum*, *Ladona fulva*, *Daphnia pulex*, *Strigamia maritima*, *Ixodes scapularis*, *Parasteatoda tepidariorum*
Opithokonta/Nematoda	*Trichinella spiralis*, *Pristionchus pacificus*
Opithokonta/Chordata	*Chrysemys picta*, *Chelonia mydas*, *Pelodiscus sinensis*, *Anolis carolinensis*, *Alligator mississippiensis*
Opithokonta/Echinodermata	*Strongylocentrotus purpuratus*, *Lytechinus variegatus*, *Patiria miniata*

*Utopia* families were found from very diverse organisms. Eukaryotes are divided into five supergroups: Excavata, Amoebozoa, Opisthokonta, SAR, and Archaeplastida [24]. *Utopia* is present in three of those supergroups. Specifically, *A*. *castellanii* belongs to Amoebozoa. Oomycetes including *P*. *infestans* belong to SAR. Animals belong to Opisthokonta.

### Insertion sites of *Utopia*

Most *Utopia* families are followed by fragments of U2 snRNA genes (Fig 1 and S1 Fig). Forty-five out of 65 *Utopia* families have at least one copy that is flanked with U2 gene fragment (Fig 1). U2 genes are observed as the 3’ flanking sequences for 44 *Utopia* families and as the 5’ flanking sequences for 11 families. Among them, U2 genes are flanked at both sides for 10 families. In the other 45 families, we could not determine the other boundary due to several reasons. In some cases, the 5’ boundaries were not sequenced. In other cases, their low copy number did not allow us to determine the 5’ ends and 5’-truncated copies were not flanked with U2 genes. *Utopia-1_NVit* from *N*. *vitripennis* is occasionally inserted in tandem in U2 genes (S1 Fig).

The 3’ flanking U2 sequences of *Utopia* families are identical to those of *Keno*, a distinct family of non-LTR retrotransposons targeting U2 genes [12]. *Utopia* is distinct from *Keno* in that *Keno* has an apurinic/apyrimidinic-like endonuclease [12] while *Utopia* encodes an endonuclease similar to PD..D/ExK-type restriction endonucleases. *Keno* and *Utopia* have evolved target specificity for the same site in parallel. *Keno* is seen from various animals including frogs, fish, lancelet and hydra [28], but not found from outside of animals. Sequences around the *Utopia* insertion sites are highly conserved throughout a wide variety of eukaryotes.

**Fig 2:**
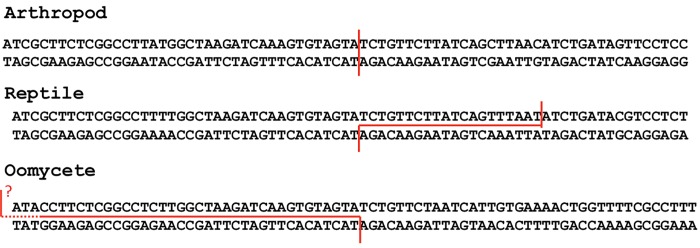
Putative cleavage sites for *Utopia* elements. Red lines indicate the cleavage sites based on the target site alterations upon insertions. Top strand cleave site is unclear for oomycete *Utopia* families due to the diversity of the 5’ flanking sequences of U2 genes.

Compared with the precise 3’ boundaries, the 5’ boundaries are varied among *Utopia* families (Fig 1), as is observed in the cases of R2 elements [22]. *Utopia* families from arthropods generate no target site duplications (TSD), while *Utopia* families from reptiles generate 20 bp TSDs. It indicates the different cleavage sites for the top strand between *Utopia* families from arthropods and reptiles (Fig 2).

In *Phytophthora*, U2 genes are often seen in an array in which different families of *Utopia* copies are inserted (S2 Fig). Because the U2 sequences downstream of *Utopia* insertions started at nucleotide 39, we expected to see the U2 gene fragment composed by nucleotides 1–38 upstream of *Utopia* insertions. However, we were not able to find U2 gene fragment upstream of *Utopia* copies; we found intact U2 genes instead. It indicates that the 5’ fragment of U2 gene is deleted upon integration. It can be explained by the top strand cleavage site upstream of the bottom strand cleavage site (Fig 2). It is considered that when the top strand cleavage site is upstream of the bottom strand cleavage site, it causes the deletion between these two cleavage sites [29]. However, the intergenic sequences of U2 genes are very diverged even without *Utopia* insertions. Besides, we could not exclude the possibility of non-homologous recombination and gene conversion between U2 genes and intervening sequences. Because of them, we could not determine the precise deletion sequences upon *Utopia* insertions. Thus it is still possible that the *Utopia* insertion duplicates a fragment of U2 gene but it is deleted due to recombination or gene conversion.

### *Utopia* families with strict target specificity for U2 snRNA genes

Five *Utopia* families are co-maintained in U2 snRNA genes in *P*. *infestans* and *P*. *ramorum*, sometimes in the same array (S2 Fig). Given the relatively low copy number of U2 genes, the maintenance of multiple *Utopia* families is not well understood. To exclude the possibility of *Utopia* insertions outside of the U2 genes, we listed all RT domain sequences of *Utopia* copies from *P*. *infestans*, *P*. *sojae*, and *P*. *ramorum* (Table 2, and S2–S4 Tables). We found a few copies per family. The most abundant *Utopia* family was *Utopia-4_PI*, but its copy number was only 14. Among those, 7 copies were sequenced from the RT domain to the 3’ terminus and all 7 were followed by fragments of U2 genes. All *Utopia* copies with intact 3’ ends were followed by U2 genes or partial U2 sequences.

**Table 2:** 3’ Flanking sequences of *Utopia* elements in three *Phytophthora* species.

	3’ Flanking sequence^1^	
Family	U2	Others
*Utopia-1_PI*	6	0
*Utopia-2_PI*	5	0
*Utopia-3_PI*	4	0
*Utopia-4_PI*	7	0
*Utopia-5_PI*	1	0
*Utopia-1_PS*	5^2^	0
*Utopia-2_PS*	1	0
*Utopia-1_PR*	2	0
*Utopia-2_PR*	2	0
*Utopia-3_PR*	3	0
*Utopia-4_PR*	3	0
*Utopia-5_PR*	1	0

1 *Utopia* copies which are 3’-truncated or whose 3’ regions are not sequenced were excluded from the analysis.

2 One *Utopia-1_PS* copy is followed by a U2 gene fragment (TCTGTTCTAATCAGTGTGAAA).

Next, we checked the copy number of U2 genes. If the genomes of *Phytophthora* contain many copies of U2 genes, target specificity for U2 genes is easily understood analogously to multiple families of R2 in ribosomal RNA genes [12][21]. We presumed that U2 sequences with intact 5’ 50 bp are functional, because all nucleotides responsible for splicing are concentrated in this region (Fig 1A). Using ~100 bp sequences of the 5’ ends of U2 genes as queries, we performed BLASTN and created a list of U2 gene sequences in the three *Phytophthora* species (S3 Fig). We found no more than 10 intact U2 genes in each genome (Table 3). Given the completed status of genome sequencing for the three *Phytophthora* species [30][31] it is likely that we found almost all U2 genes in these genomes. In all species, there are more disrupted U2 genes than intact genes. These disrupted U2 genes all lack their 5’-terminal 38 bp and are adjacent to the 3’ UTRs of *Utopia* elements. From these observations, we concluded that multiple families of *Utopia* elements are strictly U2 gene-specific and are maintained only in U2 genes at low copy numbers in *Phytophthora*.

**Table 3:** Intact and disrupted U2 snRNA genes from *Phytophthora* (oomycete) and *Nasonia* (wasp).

	*Phytophthora* ^1^			*Nasonia* ^2^		
Species	*P*. *infestans*	*P*. *sojae*	*P*. *ramorum*	*N*. *vitripennis*	*N*.*longicornis*	*N*. *giraulti*
Intact	7	10	10	15	3	2
Disrupted	41	11	14	77	7	5

1 All disrupted U2 genes are flanked by 3’ ends of *Utopia* copies.

2 Numbers of U2 genes in NCBI Trace Archives were shown. All disrupted U2 genes are flanked by 3’ ends of *Utopia* copies.

We also investigated the strictness of target specificity of *Utopia* families from three species of *Nasonia* wasps. Almost all 3’ termini of *Utopia* copies were flanked by either U2 genes or other *Utopia* copies (Table 4). Disrupted U2 genes outnumber intact U2 genes (Table 3). The data indicate that *Utopia* families from *Nasonia* are also strictly U2 gene-specific and maintained inside of U2 genes.

**Table 4:** 3’ Flanking sequences of *Utopia* elements in three *Nasonia* species.

	3’-Flanking sequences		
Species	U2	*Utopia*	Others
*N*. *vitripennis*	74	20	0
*N*. *longicornis*	7	1	1
*N*. *giraulti*	6	7	1

Numbers of 3’ junctions found in NCBI Trace Archives are shown.

### *Utopia* families with target preference for U2-like sequences

We could not detect the termini of some *Utopia* families, such as from water flea *Daphnia pulex*, green anole *Anolis carolinensis* as well as some oomycetes and insects (S1 Fig) because of their low copy numbers, old ages and/or incomplete sequence information. We also found that some *Utopia* elements were occasionally inserted outside of U2 snRNA genes. Some *Utopia* families, such as those from *Phytophthora* and *Nasonia*, are strictly inserted in U2 genes while others, such as those from reptiles and some ants, show weaker target specificity (S1 Fig). Three reported non-LTR retrotransposon families, *YURECi*, *YURE_CSa* and *YURE-2_Cis* (renamed from *R2-1a_Cis*) from sea squirts *Ciona intestinalis* and *Ciona savignyi* [12][32] are related to one another and to *Utopia* families. *YURECi* and *YURE-2_Cis* are occasionally inserted into a family of U2 gene-derived satellite repeats U2_sat, reflecting their close relationships to *Utopia* (Fig 1, and S1 Fig).

We investigated crocodilian *Utopia* insertions as representatives for weakly target-specific *Utopia* families. We analyzed *Utopia* insertions from the crocodile *Crocodylus porosus* genome and searched orthologous loci from other reptiles and birds. Due to its old age, *Utopia* copies from *C*. *porosus* can be detected by the comparison with the consensus sequence of *Utopia* copies from the alligator *Alligator mississippiensis* (*Utopia-1_AMi*). We found *Utopia* insertions that are either crocodile-specific, crocodile-and-gharial-specific, or shared by alligator, crocodile and gharial (S4 Fig), indicating that *Utopia* has been inserted outside of U2 genes since before the split of alligators and crocodiles/gharials ~103 million years ago and after the split of crocodiles and gharials, ~64 million years ago [33]. At their junctions, short sequences similar to the target site in the U2 genes, such as TGTAGTATCTG, TCTGTTCTT, and TAGTATCTATT, can be often recognized, which indicates that the target specificity of the crocodilian *Utopia* families has been weakened but not completely diminished. Recognizable target site duplications are rarely present at the junctions.

### Variable protein domain structures for *Utopia*

**Fig 3:**
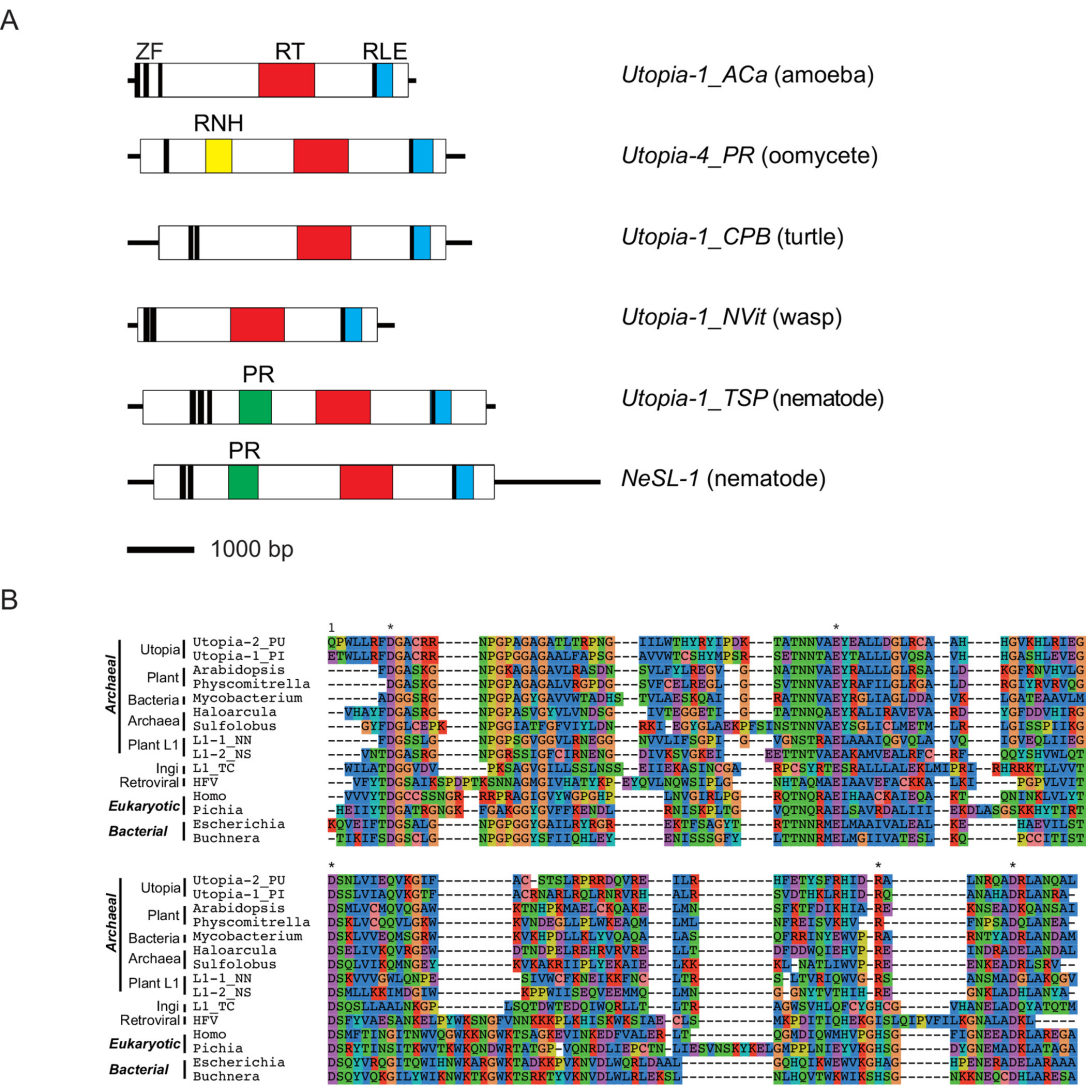
Domain organization of representative *Utopia* families. (A) The schematic structures of representative *Utopia* families. Horizontal lines indicate the full length of elements in scale. Open boxes represent protein-coding regions and filled boxes indicate domains. Black vertical lines indicate zinc finger (ZF) motifs. RT, reverse transcriptase; RLE, restriction-like endonuclease; RNH, ribonuclease H; PR, *Ulp1*-like cysteine protease. (B) Alignment of RNase H domains from various non-LTR retrotransposons, retrovirus as well as cellular RNase H domains. Conserved 5 residues (D-E-D-H/R-D) are indicated by asterisks.

*Utopia* families generally encode one protein that includes one to three zinc finger (ZF) motifs at the N-terminus, an RT domain at the middle and one ZF and an RLE domain at the C-terminus (Fig 3). *Utopia-1_TSP* from the nematode *Trichinella spiralis* shows an N-terminal *Ulp1*-like cysteine protease domain (Fig 3). *NeSL* families from nematodes also contain an N-terminal *Ulp1*-like cysteine protease domain [13]. The protein sequence alignment revealed that these cysteine protease domains are similar to one another (S5 Fig). *Utopia-1_PPac* from another nematode *Pristionchus pacificus* lacks a protease domain.

**Fig 4:**
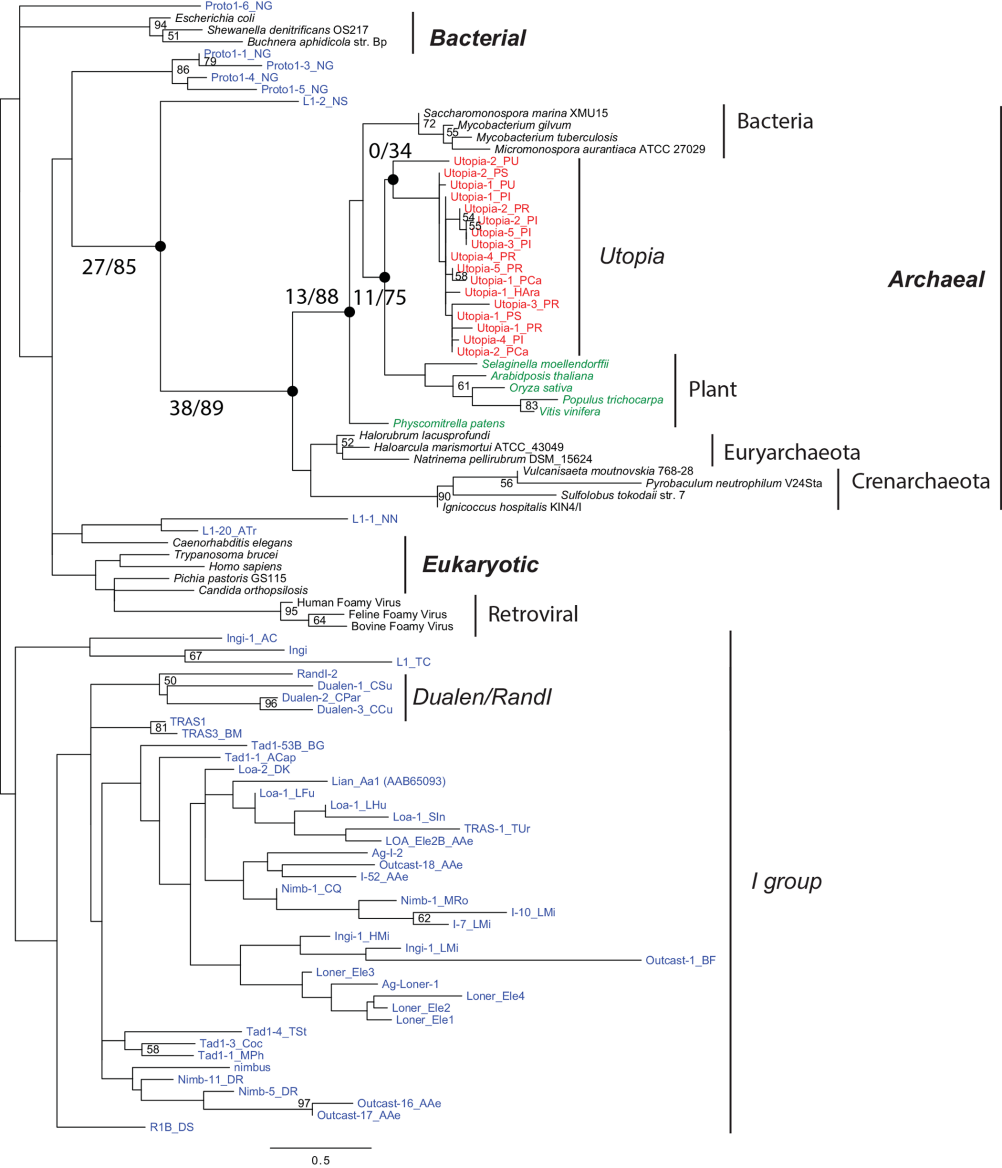
The phylogeny of RNase H domains from oomycete *Utopia* families, other non-LTR retrotransposons, and cellular RNase H genes. Cellular RNase H domains used here are identical to those in [34]. Numbers at nodes indicate bootstrap values of 100 replicates; only values over 50 are shown. We show bootstrap values and aLRT values at 5 nodes. The RNase H domains of *Utopia* are colored in red, those of plant are in green and those of other non-LTR retrotransposons are in blue.

*Utopia* families from oomycetes all encode a ribonuclease H (RNase H) domain upstream of RT. The position of RNase H in oomycete *Utopia* families, upstream of the RT domain, is unique. Besides, *Utopia* families outside of oomycetes do not encode RNase H. These facts indicate that the acquisition of RNase H is a relatively recent event in the *Utopia* evolution. RNase H has been reported from various clades of non-LTR retrotransposons, the *I* group (the clades *Ingi*, *I*, *Nimb*, *Loa*, *R1*, *Tad1*, *Loner*, and *Outcast*), plant *L1*, *Proto1* and *Dualen/RandI* [4,5][34]. We performed the phylogenetic analysis of RNase H domains from various non-LTR retrotransposons with representatives of cellular RNase H (Fig 4). Due to the short sequences of RNase H, the statistical supports are weak. Yet the RNase H domains of *Utopia* are close to archaeal-like RNase H from plants in the phylogeny. Smyshlyaev et al. [34] reported that plant L1 has an RNase H similar to archaeal RNase H. Our analysis is not intended to reanalyze their data, but we did not observe the cluster of archaeal RNase H and plant L1 RNase H supported by bootstrap analysis. The phylogenetic analysis in their article was supported not by bootstrap values, but by the approximate likelihood-ratio test of the branches (aLRT). We got higher numbers of statistical supports when we use aLRT (Fig 4). aLRT values support the position of *Utopia* RNase H inside of archaeal RNase H. One of the reported characteristics shared among archaeal-like RNase H is the replacement of histidine (H) of the conserved 5 residues D-E-D-H-D by arginine (R) [34]. Accordingly, the RNase H of *Utopia* contains R at the position of H (Fig 3B). Based on these data, we hypothesize that oomycete *Utopia* elements acquired cellular archaeal-like RNase H domains relatively recently, possibly from a plant that oomycetes infected, though the origin of RNase H domains of plant L1 as well as those of *Utopia* remains to be clarified.

### Phylogenetic analysis of RT

**Fig 5:**
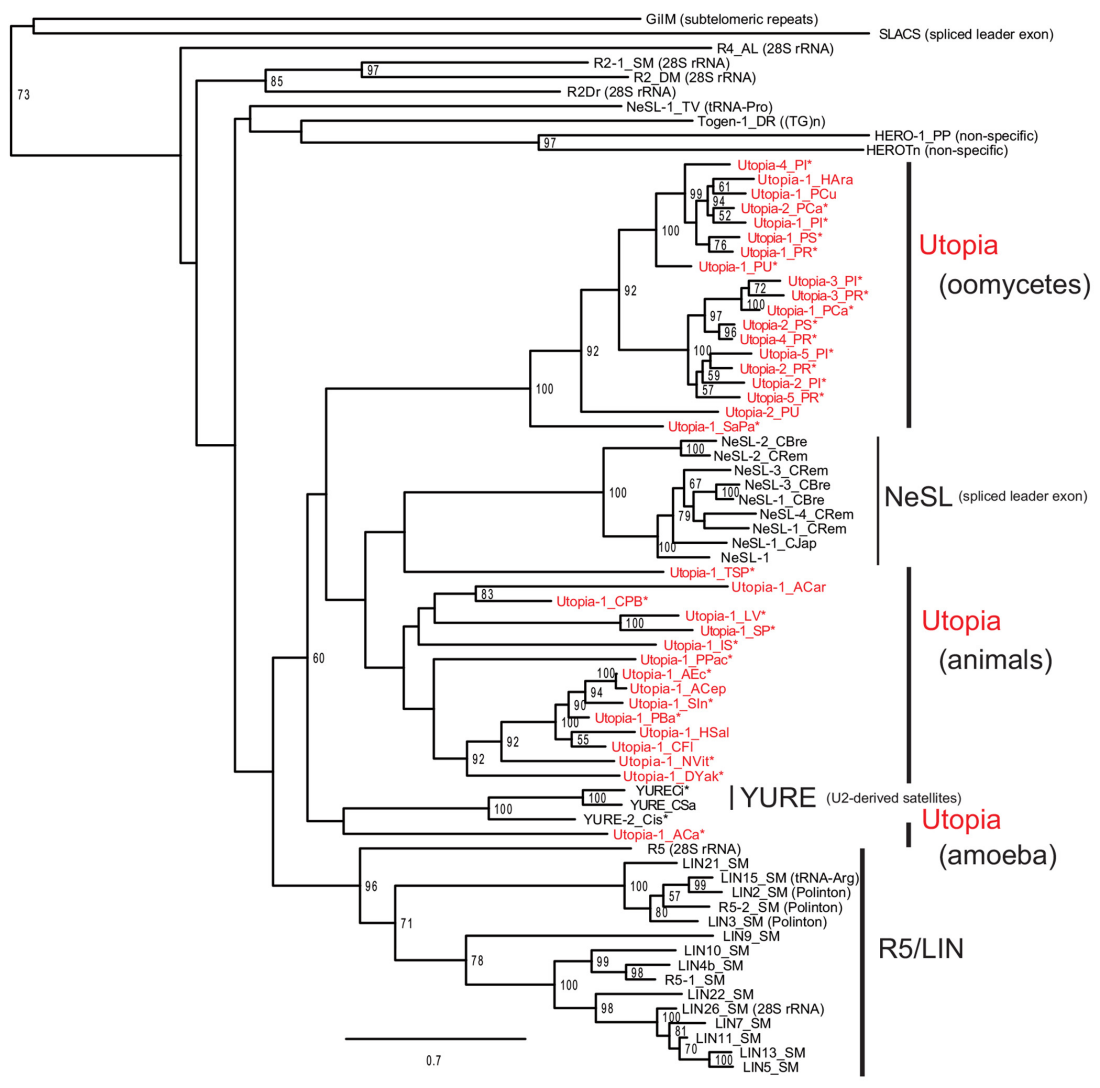
The phylogeny of *Utopia* and other non-LTR retrotransposons related to the *NeSL* clade. SLACS and GilM were used as outgroups [4],[46]. Numbers at nodes indicate bootstrap values of 100 replicates; only values over 50 are shown. Asterisks indicate *Utopia* families of which at least one copy is flanked by U2 gene sequences. Target repeats are shown in parentheses after family names other than *Utopia*.

To determine the phylogenetic position of *Utopia* and the origin of its target specificity, we performed phylogenetic analysis based on the RT domain sequences (Fig 5). Preliminary analysis indicated that *Utopia* is close to *NeSL*. Thus first we determined the target specificity of families related to *NeSL*. *NeSL-1_TV* was revealed to be tRNA-Pro gene-specific and *Togen* families (*Togen-1_DR*, *Togen-1_SSa*, *Togen-1_OM*, *Togen-1_GMo*, and *Togen-1_CCar*; all from fish) are (TG)_n_ microsatellite-specific (S6 Fig). *LIN* families from planaria also show new target specificities: for 28S rRNA genes (*LIN9_SM*, *LIN24_SM*, *LIN26_SM*), for tRNA-Arg genes (*LIN15_SM*), and for tRNA-Glu genes (*LIN25_SM*) (S6 Fig).

Historically, *NeSL-1* and *R5* are classified into the *NeSL* clade [13,35]. In Fig 5, however, the monophyly of *NeSL-1* and *R5* is not well supported. Nevertheless, in this article, we prefer to keep the *NeSL* clade as including both *NeSL-1* and *R5*. All *Utopia* families are positioned in the lineage that both *NeSL-1* and *R5* belong to. Thus here, we claim that *Utopia* belongs to the *NeSL* clade. We did not get strong supports for the phylogenetic positions of *NeSL-1_TV* and *Togen* families. Their phylogenetic positions and the relationships to the *HERO* and *NeSL* clades are still to be resolved.

Families that share target specificity with *NeSL-1* are clustered well (NeSL in Fig 5). In addition to *NeSL*, several clusters are supported by high bootstrap values: oomycete *Utopia* (100%), insect *Utopia* (92%), *YURE* (100%), and *R5/LIN* (96%). The target specificity has been diversified in the *LIN/R5* lineage of platyhelminthes. *Utopia* families from *Phytophthora* were clustered into two lineages, one of which includes families from several different genera: *Utopia-1_PU* from *Pythium*, *Utopia-1_PCu* from *Pseudoperonospora*, and *Utopia-1_HAra* from *Hyaloperonospora*. This indicates long-term maintenance of multiple *Utopia* lineages in oomycetes.

*Utopia*, *NeSL* and *YURE* are clustered together with bootstrap support at 60%. It is not high enough to conclude their monophyly, but the deep phylogeny of non-LTR retrotransposons are not always supported by ample bootstrap values due to the limit of alignable sites and their old ages [22][36]. As described above, *YURE* families show some preference for U2 snRNA gene-derived satellites (Fig 1 and S1 Fig). It is consistent with the phylogenetic position of *YURE*. *Utopia-1_TSP* from a nematode *Trichinera spiralis* positioned close to the lineage of spliced leader-specific *NeSL* families from nematodes. The data combined with the presence of a protease domain indicates that *NeSL* families branched off *Utopia* by changing their target specificities. *Utopia-1_PPac* does not cluster with other nematode families, showing that nematodes have two different lineages of *Utopia/NeSL*.

It is reasonable to consider that the common ancestor of *Utopia*, *NeSL* and *YURE* was U2 snRNA gene-specific. *NeSL* is narrowly distributed, only in nematodes, and their protein structure is similar to that of *Utopia-1_TSP*. *YURE* was found from sea squirts and it shows relatively weak specificity to U2 snRNA gene-derived satellites. *Utopia* is the most widely distributed and show target specificity for U2 snRNA gene at some extent. Although horizontal transfer of *Utopia* between eukaryotic supergroups is not excluded due to its sporadic distribution, our data indicate that the common ancestor of *Utopia* was present before the divergence of major eukaryotic supergroups.

### Maintenance of target specificity for a low-copy-number repeat

The target specificity for a low-copy-number repeat, such as U2 snRNA genes, does not seem a good survival strategy for transposons. Indeed, compared to transposons targeting rRNA genes, transposons targeting snRNA genes are few. Only *Keno* and *Utopia* for U2, *Dada-U1* for U1 and *Dada-U6* for U6 have been reported [12][17]. The low copy number of target repeat is likely related to the frequency of families that show weak target specificity in the *Utopia* lineage.

In such condition, the maintenance of multiple lineages of *Utopia* in oomycetes is a surprise. Multiple families of *Utopia* have been maintained in the genomes of several *Phytophthora* species. All *Utopia* copies are flanked with U2 snRNA genes. Besides, the phylogenetic analysis revealed that at least two lineages of *Utopia* have been maintained in oomycetes since before the divergence of several genera. It is hard to consider that 5 *Utopia* families have been maintained in the genome of *Phytophthora* with around 10 copies of intact U2 genes. To understand the maintenance of *Utopia* in a low-copy-number repeat family, we consider that the concept of population genetics is necessary.

It is proved by mathematical study that homing endonuclease genes (HEG) can be persisted without horizontal transfer in some conditions [37]. HEG is a group of genetic parasites that is present at a specific single-copy locus [38]. HEG is usually coupled with self-splicing intron or intein, which allows it to be spliced out when transcribed. Due to this, HEG is considered to be more harmless compared to target-specific non-LTR retrotransposons. However if it is fixed, it is susceptible to degeneration because no empty target site is present.

Similar to HEG, *Utopia* is probably persisted unfixed. Oomycetes are diploid with both asexual and sexual reproduction. *Utopia* can be present not evenly in each haploid genome. Sexual reproduction shuffles *Utopia* insertions, and the cell lines that have too few intact U2 genes go to extinct, while those with more intact U2 genes can replicate. Our results may attract theoretical studies necessary for further understanding of the survival and evolution of transposons.

## Conclusions

*Utopia* is the first lineage of target-specific non-LTR retrotransposons found in diverse eukaryotes beyond major eukaryotic supergroups. The phylogeny suggests that they share the common origin of target specificity. Our analysis revealed that target sequence specificity for multicopy genes is a strategy for non-LTR retrotransposons even for low copy number genes, and its origin likely dates back to the early evolution of eukaryotes.

## Methods

Genomic sequences of various species were obtained mostly from NCBI GenBank, and sequences of known non-LTR retrotransposons were obtained from Repbase [1] (http://www.girinst.org/repbase). The crocodilian genome sequences were sequenced by the International Crocodilian Genomes Working Group [39]. The sequences of retrotransposons reported in this work are deposited in Repbase (http://www.girinst.org/repbase).

New non-LTR retrotransposons were identified by repeated BLAST [40] and CENSOR [41] searches using genomic sequences of various species with known elements as queries. The consensus sequences were derived using the majority rule applied to the corresponding set of multiple aligned copies of retrotransposons.

Representative RNase H sequences for each clade of non-LTR retrotransposons were chosen randomly from Repbase. The data set of cellular RNase H domains reported in Smyshlyaev et al. [34] were used. The RNase H domain sequences of non-LTR retrotransposons were aligned with the aid of MAFFT [42]. Gblocks was used to choose comparable sites for phylogenetic analysis [43]. It took 42 sites for the phylogenetic analysis with the least strict options. ProtTest was performed at the ProtTest server (http://darwin.uvigo.es/software/prottest2_server.html) and it indicated LG+I+G is the most appropriate model in the Akaike Information Criterion and Baysian Information Criterion [44]. A maximum likelihood tree was constructed by PhyML [45] with bootstrap values (1000 replicates) using the model LG+I+G.

The RT domain sequences of non-LTR retrotransposons spanning motif 0 to 9* [4] were aligned with the aid of MAFFT [42]. We excluded *Utopia* families whose sequences show some ambiguity caused by old ages or incomplete sequencing. Other than *Utopia*, all non-LTR retrotransposons reported to belong to the *NeSL* clade in Repbase were used. SLACS and GilM were used as outgroups [4],[46]. ProtTest indicated LG+I+G+F is the most appropriate model based on the Akaike Information Criterion and LG+I+G on the Baysian Information Criterion [44]. Maximum likelihood trees were constructed by PhyML [45] with bootstrap values (100 replicates) using the amino acid substitution model LG+I+G.

The phylogenetic trees were drawn with the aid of FigTree 1.3.1 (http://tree.bio.ed.ac.uk/software/figtree/).

## Supporting Information

S1 Fig The 3’ junction sequences for *Utopia* insertions. The sequences hit by censor search with the 3' terminal 70 bps of *Utopia* elements are shown with their 3' flanking sequences. Accession numbers and the positions for the 3' terminal 70bp of *Utopia* elements (in parentheses) are shown. If there are more than 20 copies, the top 20 hits are shown. If the top 20 hits do not include copies flanked with U2 genes, representative insertions flanked by U2 genes are also shown. The nucleotides of *Utopia* are in blue while nucleotides of U2 genes or U2_sat are in red.
(PDF)

S2 Fig Tandem arrays of *Utopia* elements and U2 snRNA genes in three *Phytophthora* species. (A) Schematic structure of U2 gene tandem arrays. (B) Alignment of U2 snRNA genes shown in A. U2 sequences are shaded. Names in parentheses indicate the 5’ flanking *Utopia* elements of U2 snRNA gene fragments.
(PDF)

S3 Fig All U2 sequences in three species of *Phytophthora*: *P*. *infestans*, *P*. *sojae* and *P*. *ramorum*. The sequences of U2 snRNA genes are shown in uppercase. Unsequenced regions are shown by “n”.
(PDF)

S4 Fig Non-U2 insertions of crocodilian *Utopia* elements. The sequence accession numbers or scaffold numbers, and nucleotide positions are shown below each alignment. Sequences similar to the specific insertion site in the U2 genes are underlined. Nucleotides of *Utopia* insertions are colored in blue. TSDs are in red. Sequences representing the original uninserted state of the locus, such as the orthologous loci from turtles, birds or mammals and the consensus sequences for transposable elements, are shown if available.
(PDF)

S5 Fig Alignment of *Ulp1*-like protease domains of *Utopia-1_TSP* and *NeSL* elements as well as cellular *Ulp1* proteins. (PDF)

S6 Fig Target sequences of *NeSL*-related non-LTR retrotransposons. Retrotransposon sequences are in lowercase while flanking sequences are in uppercase. Sequences similar to target sequences are in red.
(PDF)

S1 Table *Utopia* transposons used in this study. (PDF)

S2 Table All RT-coding sequences of *Utopia* elements in *P*. *infestans*. (PDF)

S3 Table All RT-coding sequences of *Utopia* elements in *P*. *sojae*. (PDF)

S4 Table All RT-coding sequences of *Utopia* elements in *P*. *ramorum*. (PDF)
