# Supplementary material for: Ancient Origin of the U2 Small Nuclear RNA Gene-Targeting Non-LTR Retrotransposons Utopia
Source: PLoS One. 2015 Nov 10;10(11):e0140084. doi: 10.1371/journal.pone.0140084 (PMC4640811; doi:10.1371/journal.pone.0140084)
Supplement: S1 Fig — The sequences hit by censor search with the 3' terminal 70 bps of Utopia elements are shown with their 3' flanking sequences. Accession numbers and the positions for the 3' terminal 70bp of Utopia elements (in parentheses) are shown. If there are more than 20 copies, the top 20 hits are shown. If the top 20 hits do not include copies flanked with U2 genes, representative insertions flanked by U2 genes are also shown. The nucleotides of Utopia are in blue while nucleotides of U2 genes or U2_sat are in red. (PDF) [file pone.0140084.s001.pdf]

S1 Figure.

***Acanthamoeba castellanii* (amoeba)**

**Utopia-1_ACa**	ACATCAGAGTTCCTGCGAAGGGATACACTCTGCCAATCTCGTGGGTTGTAATAAATCCACACCTTCAACA
U2 snRNA gene (Acanthamoeba)	------------------------------GGCATCTTCTCGGCCTTCGTGGCTAAGATCAAGTGTAGTATCTGTTCTTATCAGCTTAATCTCTGGTAGTGAGGCCA
AEYA01001147_[475918-475987]	ACATCAGAGTTCCTGCGAAGGGATACACTCTGCCAATCTCGTGGGTTGTAATAAATCCACACCTTCAACATCTGTTCTTATCAGCTTAATCTCTGGTAGTGAAGCGT
AHJI01001615_[61773-61842]	ACATCAGAGTTCCTGCGAAGGGATACACTCTGCCAATCTCGTGGGTTGTAATAAATCCACACCTTCAACATCTGTTCTTATCAGCTTAATCTCTGGTAGTGAAGCGT

***Phytophthora infestans* (oomycete)**

**Utopia-1_PI**	AGTGACATACATATTTTAGCCTAGATTTCAGTTACGGAGAGGTTACTAACTGGTAAATACGAACACATAT
U2 snRNA gene (P.infestans)	--------------------------------ATACCTTCTCGGCCTCTTGGCTAAGATCAAGTGTAGTATCTGTTCTAATCATTGTGAAAACTGGTTTTCGCCTTT
AATU01000132_[794-863]	AGTGACATACATATTTTAGCCTAGATTTCAGTTACGGAGAGGTTACTAACTGGTAAATACGAACACATA-TCTGTTCTAATCAGTGTGAAAACTGGTTTTCGCCTTT
AATU01000163_[2968-3037]	AGTGACATACATATTTTAGCCTAGATTTCAGTTACGGAGAGGTTACTAACTGGTAAATACGAACACATATTCTGTTCTAATCAGTGTGAAAACTGGTTTTCGCCTTT
AATU01001281_[4653-4722]	AGTGACATACATATTTTAGCCTAGATTTCAGTTACGGAGAGGTTACTAACTGGTAAATACGAACACATATTCTGTTCTAATCAGTGTGAAAACTGGTTTTCGCCTTT
AATU01001283_[28417-28486]	AGTGACATACATATTTTAGCCTAGATTTCAGTTACGGAGAGGTTACTAACTGGTAAATACGAACACATATTCTGTTCTAATCAGTGTGAAAACTGGTTTTCGCCTTT
AATU01001288_[3877-3808]	AGTGACATACATATTTTAGCCTAGATTTCAGTTACGGAGAGGTTACTAACTGGTAAATACGAACACATATTCTGTTCTAATCAGTGTGAAAACTGGTTTTCGCCTTT
AATU01001290_[2248-2179]	AGTGACATACATATTTTAGCCTAGATTTCAGTTACGGAGAGGTTACTAACTGGTAAATACGAACACATATTCTGTTCTAATCAGTGTGAAAACTGGTTTTCGCCTTT
AATU01001290_[15341-15272]	AGTGACATACATATTTTAGCCTAGATTTCAGTTACGGAGAGGTTACTAACTGGTAAATACGAACACATATTCTGTTCTAATCAGTGTGAAAACTGGTTTTCGCCTTT
AATU01001295_[2315-2246]	AGTGACATACATATTTTAGCCTAGATTTCAGTTACGGAGAGGTTACTAACTGGTAAATACGAACACATATTCTGTTCTAATCAGTGTGAAAACTGGTTTTCGCCTTT
AATU01000133_[1751-1682]	AGTGACATCCATATTTTAGCCTAGATTTCAGTTACGGAGAGGTTACTAACTGGTAAATACGAACACATATTCTGTTCTAATCAGTGTGAAAACTGGTTTTCGCCTTT
AATU01000138_[8748-8817]	AGTGACATACATATTTTAGCATAGATTTCAGTTACGGAGAGGTTACTAACTGGTAAATACGAACACATATTCTGTTCTAATCAGTGTGAAAACTGGTTTTCGCCTTT
AATU01001290_[8778-8719]	----------ATATTTTAGCCTAGATTTCAGTTACGGAGAGGTTACTAACTGGTAAATACGAACACATATTCTGTTCTAATCAGTGTGAAAACTGGTTTTCGCCTTT
AATU01001281_[24721-24790]	AGTGACATACATATTTTAGCCTAGATTTCAGTTACGGAGAGGTTACTAACTGGTAAATACGAACACAT
	--------------------------------ATACCTTCTCGGCCTCAAGGCTAAGATCAAGTGTAGTATCTGTTCTAATCAGTGTGAAAACTGGTTTTCGCCTTT

**Utopia-2_PI**	CATTCAATTAGGTAGCGACCAAATTACAAATTTGGTAACGAGTAAGCCAAATGGTAATACACAAAACTTT
U2 snRNA gene (P.infestans)	--------------------------------ATACCTTCTCGGCCTCTTGGCTAAGATCAAGTGTAGTATCTGTTCTAATCATTGTGAAAACTGGTTTTCGCCTTT
AATU01000162_[836-905]	CATTCAATTAGGTAGCGACCAAATTACAAATTTGGTAACGAGTAAGCCAAATGGTAATACACAAAACTTTTCTGTTCTAATCAGTGTGAAAACTGGTTTTCGCCTTT
AATU01000164_[6222-6291]	CATTCAATTAGGTAGCGACCAAATTACAAATTTGGTAACGAGTAAGCCAAATGGTAATACACAAAACTTTTCTGTTCTAATCAGTGTGAAAACTGGTTTTCGCCTTT
AATU01001283_[7966-8035]	CATTCAATTAGGTAGCGACCAAATTACAAATTTGGTAACGAGTAAGCCAAATGGTAATACACAAAACTTTTCTGTTCTAATCAGTGTGAAAACTGGTTTTCGCCTTT
AATU01001283_[14851-14920]	CATTCAATTAGGTAGCGACCAAATTACAAATTTGGTAACGAGTAAGCCAAATGGTAATACACAAAACTTTTCTGTTCTAATCAGTGTGAAAACTGGTTTTCGCCTTT
AATU01001287_[10463-10394]	CATTCAATTAGGTAGCGACCAAATTACAAATTTGGTAACGAGTAAGCCAAATGGTAATACACAAAACTTTTCTGTTCTAATCAGTGTGAAAACTGGTTTTCGCCTTT
AATU01001299_[486-417]	CATTCAATTAGGTAGCGACCAAATTACAAATTTGGTAACGAGTAAGCCAAATGGTAATACACAAAACTTTTCTGTTCTAATCAGTGTGAAAACTGGTTTTCGCCTTT
AATU01004588_[507-438]	CATTCAATTAGGTAGCGACCAAATTACAAATTTGGTAACGAGTAAGCCAAATGGTAATACACAAAACTTTTCTGTTCTAATCAGTGTGAAAACTGGTTTTCGCCTTT
AATU01000166_[1773-1842]	CATTCAATTAGGTAGCGACCAAATTACAAATTTGGTAACGAGTAAGCCAAATGGTAATACACAAAACTC-TCTGTTCTAATCAGTGTGAAAACTGGTTTTCGCCTTT
AATU01000169_[752-821]	CATTCAATTAGGTAGCGACCAAATTACAAATTTGGTAACGAGTAAGCCAAATGGTAATACACAAAACTC-TCTGTTCTAATCAGTGTGAAAACTGGTTTTCGCCTTT
AATU01000150_[2465-2396]	CATTAAATTAGGTAGCGACCAAATTACAAATTTGGTAACGAGTAAGCCAAATGGTAATACACAAAACTTTTCTGTTCTAATCAGTGTGAAAACTGGTTTTCGCCTTT
AATU01000141_[3850-3919]	CATTCAATTAGGTAGCGACCAAATCACAAATTTGGTAACGCGTAAGCCAAATGGTAATACACAAAACTTTTCTGTTCTAATCAGTGTGAAAACTGGTTTTCGCCTTT
AATU01000173_[1020-951]	CATTTAATTAGGTAGCGACCAGATTACAAACTTGGTAACGAGTAAGCCAAATGGTAATACACAAAACTTTTCTGTTTTAATCAGTGTGAAAACTGGTTTTCGCCTTT
AATU01000143_[7898-7829]	CATTAAATTAGGTAGCGACCAAATTACAAATTTGTTAACGAGTAAGCCAAATGGTAATACACAAAACTTTTCTGTTCTAATCAGTGTGAAAACTGGTTTTCGCCTTT
AATU01000145_[3880-3811]	CATTAAATTAGGTAGCGACCAAATTACAAATTTGGTAACGAGTGAGCCAAATGGTAATACACAAAAATT-TCTGTTCTAATCAGTGTGAAAACTGGTTTTCGCCTTT
AATU01000149_[569-500]	CATTAAATTAGGTAGCGACCAAATTACAAATTTGTTAGCGAGTAAGCCAAATGGTAATACACAAAACTTTTCTGTTCTAATCAGTGTGAAAACTGGTTTTCGCCTTT
AATU01000141_[15317-15248]	CATTAAATTACGTAGCGACCAAATTACAAATTTGTTAACGAGTAAGCCAAATGGTAATACACAAAACTTTTCTGTTCTAATCAGTGTGAAAACTGGTTTTCGCCTTT
AATU01000156_[1090-1026]	-----AATTAGGTAGCGACCAAATTACAAATTTGGTAACGAGTAAGCCAAATAGTAATACACAAAACGTTTCTGTTCTAATCAGTGTAAAAACTGGTTTTCGCCTTT
AATU01000128_[689-752]	-----AATTAGGTAGCGACCAAATTACAA-TTTGGTAACGAGTAAGCCAAATGGTAATGCACAAAACTTTTCTGC-TCTAATCAGTGGGAAAACTGTTTTCCGCCTT
AATU01002598_[2606-2540]	CATTCA-TTAGG-AGCGACCAAATTACAA-TTTGGTAACGAGTAAGCCAAATGGTAATACACAAAACTTTTCTGTTCTAATCAGTGTGAAAACTGGTTTTCGCCTTT
AATU01000176_[666-604]	CATTCAATTAGGCAGCG------TACAAA-TTTGGTAACGAGTAAGCAAAATGGTAATACACAAAACTTTTCTGTTCTAATCAGTGTGAAAACTGGTTTTCGACTTT

**Utopia-3_PI**	TAGATATCAGATAGGGAACCTTTATTAGGGAGATAACGGGTACACCGGATGGTAAATATACAAAACCTTC
U2 snRNA gene (P.infestans)	--------------------------------ATACCTTCTCGGCCTCTTGGCTAAGATCAAGTGTAGTATCTGTTCTAATCATTGTGAAAACTGGTTTTCGCCTTT
AATU01001281_[11633-11702]	TAGATATCAGATAGGGAACCTTTATTAGGGAGATAACGGGTACACCGGATGGTAAATATACAAAACCTTCTCTGTTCTAATCAGTGTGAAAACTGGTTTTCGCCTTT
AATU01001283_[21877-21946]	TAGATATCAGATAGGGAACCTTTATTAGGGAGATAACGGGTACACCGGATGGTAAATATACAAAACCTTCTCTGTTCTAATCAGTGTGAAAACTGGTTTTCGCCTTT
AATU01001290_[21840-21771]	TAGATATCAGATAGGGAACCTTTATTAGGGAGATAACGGGTACACCGGATGGTAAATATACAAAACCTTCTCTGTTCTAATCAGTGTGAAAACTGGGTTTCGCCTTT
AATU01001290_[28899-28830]	TAGATATCAGATAGGGAACCTTTATTAGGGAGATAACGGGTACACCGGATGGTAAATATACAAAACCTTCTCTGTTCTAATCAGTGTGAAAACTGGTTTTCGCCTTT
AATU01001291_[842-773]	TAGATATCAGATAGGGAACCTTTATTAGGGAGATAACGGGTACACCGGATGGTAAATATACAAAACCTTCTCTGTTCTAATCAGTGTGAAAACTGGTTTTCGCCTTT
AATU01001297_[1488-1419]	TAGATATCAGATAGGGAACCTTTATTAGGGAGATAACGGGTACACCGGATGGTAAATATACAAAACCTTCTCTGTTCTAATCAGTGTGAAAACTGGTTTTCGCCTTT
AATU01001292_[6217-6149]	TAGATATCAGATAGGGAACCTTTATTAGGGAGATAACGGGTACACCGGATG-TAAATATACAAAACCTTCTCTGTTCTAATCAGTGTGAAAACTGGTTT-CGCCTTT

**Utopia-4_PI**	AGTGACATACATATTTTAGCCTAGATTTCAGTTACGGAGAGGTTACTAACTGGTACATAAAATTACACAT
U2 snRNA gene (P.infestans)	--------------------------------ATACCTTCTCGGCCTCTTGGCTAAGATCAAGTGTAGTATCTGTTCTAATCATTGTGAAAACTGGTTTTCGCCTTT
AATU01001281_[18194-18263]	AGTGACATACATATTTTAGCCTAGATTTCAGTTACGGAGAGGTTACTAACTGGTACATAAAATTACACATTCTGTTCTAATCAGTGTGAAAACTGGTTTTCGCCTTT
AATU01001283_[1136-1205]	AGTGACATACATATTTTAGCCTAGATTTCAGTTACGGAGAGGTTACTAACTGGTACATAAAATTACACATTCTGTTCTAATCAGTGTGAAAACTGGTTTTCGCCTTT
AATU01001287_[1146-1077]	AGTGACATACATATTTTAGCCTAGATTTCAGTTACGGAGAGGTTACTAACTGGTACATAAAATTACACATTCTGTTCTAATCAGTGTGAAAACTGGTTTTCGCCTTT
AATU01001295_[8860-8791]	AGTGACATACATATTTTAGCCTAGATTTCAGTTACGGAGAGGTTACTAACTGGTACATAAAATTACACATTATGTTCTAATCAGTGTGAAAACTGGTTTTCGCCTTT
AATU01016980_[2343-2274]	AGTGACATACATATTTTAGCCTAGATTTCAGTTACGGAGAGGTTACTAACTGGTACATAAAATTACACATTCTGTTCTAATCAGTGTGAAAACTGGTTTTCGCCTTT
AATU01000137_[6083-6152]	AGTGACATACATATTTTAGCATAGATTTCAGTTACGGAGAGGTTACTAACTGGTACATAAAATTACACATTCTGTTCTAATCAGTGTGAAAACTGGTTTTCGCCTTT
AATU01000138_[2089-2158]	AGTGACATACATATTTTAGTATAGATTTCAGTTACGGAGAGGTTACTAACTGGTACATAAAATTACACATTGTGTTCTAATCAGTGTGAAAACTGGTTTTCGCCTTT
AATU01000138_[5435-5504]	AGTGACATACATATTTTAGTATAGATTTCAGTTACGGAGAGGCTACTAACTGGTACATAAAATTACACATTGTGTTCTAATCAGTGTGAAAACTGGTTTTCGCCTTT
AATU01010950_[5047-5116]	AGTGACATGCATATTTTAGCCTAGATTTCAGTTACGGAGAGGTTACTAACTGGTACATACACTTACACATTATGTTCTAATCAGTGTGAAAACTAGTTTCCGTCTTT
AATU01000181_[12715-12784]	AGTGACATGCATATTTTAGCCTAGATTTCAGTTACGGAGTGGTTACTTACTGGTACATACACTTACACATTATGTTCTTATCAGTGTGAAAACTAGTTTCCGTCTTT

**Utopia-5_PI**	ATATTTTAGACGTAGCGACCAAATTACAAATTTGGTAACGAGTAAGCCAAATGGTAATACACAAAACTTT
U2 snRNA gene (P.infestans)	--------------------------------ATACCTTCTCGGCCTCTTGGCTAAGATCAAGTGTAGTATCTGTTCTAATCATTGTGAAAACTGGTTTTCGCCTTT
AATU01010946_[527-458]	ATATTTTAGACGTAGCGACCAAATTACAAATTTGGTAACGAGTAAGCCAAATGGTAATACACAAAACTTTTCTGTTCTAATCAGTGTGAAAACTGGTTTTCGCCTTT
AATU01000164_[6-38]	--------------------------------------ACGAGTAAGCCAAATGGTAATACACAAAACTCTCTGTTCTAATCAGTGTGAAAACTGGTTTTCGCCTTT

***Phytophthora sojae* (oomycete)**

**Utopia-1_PS**	AGTGACATGCATATTTTAGCCTACATTTCAGTTACGGAGAGGTTACTAACTGGTAAACACGAACACACAT
U2 snRNA gene (P.infestans)	--------------------------------ATACCTTCTCGGCCTCTTGGCTAAGATCAAGTGTAGTATCTGTTCTAATCATTGTGAAAACTGGTTTTCGCCTTT
AAQY01000581_[1405-1474]	AGTGACATGCATATTTTAGCCTACATTTCAGTTACGGAGAGGTTACTAACTGGTAAACACGAACACACATTCTGTTCTAATCAGTGTGAAAACTGGTTTCCCTGCTT
AAQY01000741_[374-443]	AGTGACATGCATATTTTAGCCTACATTTCAGTTACGGAGAGGTTACTAACTGGTAAACACGAACACACATTCTGTTCTAATCAGTGTGAAAACTGGTTTCCCTGCTT
AAQY01000742_[2350-2419]	AGTGACATGCATATTTTAGCCTACATTTCAGTTACGGAGAGGTTACTAACTGGTAAACACGAACACACATTCTGTTCTAATCAGTGTGAAAACTGGTTTCCCTGCTT
AAQY01001201_[145529-145460]	AGTGACATGCATATTTTAGCCTACATTTCAGTTACGGAGAGGTTACTAACTGGTAAACACGAACACACATTCTGTTCTAATCAGTGTGAAAACTGGTTTCCCTGCTT
AAQY01001201_[148837-148768]	AGTGACATGCATATTTTAGCCTACATTTCAGTTACGGAGAGGTTACTAACTGGTAAACACGAACACACATTCTGTTCTAATCGGTGTGAAAACTGGTTTCCCTGCTT
AAQY01001202_[551-482]	AGTGACATGCATATTTTAGCCTACATTTCAGTTACGGAGAGGTTACTAACTGGTAAACACGAACACACATTCTGTTCTAATCAGTGTGAAAACTGGTTTCCCTGCTT
AAQY01002698_[579-510]	AGTGACATGCATATTTTAGCCTACATTTCAGTTACGGAGAGGTTACTAACTGGTAAACACGAACACACATTGTGTTCTAATCGGTGTGAAAACTGGTTTCCCTGCTT

**Utopia-2_PS**	CAATTAGGGACTCCCAGGTAGTCACTACCAGGACGCAACGGACTAACCGGCAGGTAATACATTAACCCTT
U2 snRNA gene (P.infestans)	--------------------------------ATACCTTCTCGGCCTCTTGGCTAAGATCAAGTGTAGTATCTGTTCTAATCATTGTGAAAACTGGTTTTCGCCTTT
AAQY01000576_[3334-3403]	CAATTAGGGACTCCCAGGTAGTCACTACCAGGACGCAACGGACTAACCGGCAGGTAATACATTAACCCTTTCTGTTCTAATCAGTGTGAAAACTGGTTTCCCTGCTT
AAQY01000577_[4663-4732]	CAATTAGGGACTCCCAGGTAGTCACTACCAGGACGCAACGGACTAACCGGCAGGTAATACATTAACCCTTTCTGTTCTAATCAGTGTGAAAACTGGTTTCCCTGCTT
AAQY01000578_[1145-1214]	CAATTAGGGACTCCCAGGTAGTCACTACCAGGACGCAACGGACTAACCGGCAGGTAATACATTAACCCTTTCTGTTCTAATCAGTGTGAAAACTGGTTTCCCTGCTT
AAQY01002699_[721-691]	---------------------------------------GGACTAACCGGCAGGTAATACATTAACCCTTTCTGTTCTAATCAGTGTGAAAACTGGTTTCCCTGCTT

***Phytophthora ramorum* (oomycete)**

**Utopia-1_PR**	TAGTGACATGCATATTTTAGCCTAGATTTCAGTTACGGAGAGGTTACTAACTGGTAACACGAAAACACAT
U2 snRNA gene (P.infestans)	--------------------------------ATACCTTCTCGGCCTCTTGGCTAAGATCAAGTGTAGTATCTGTTCTAATCATTGTGAAAACTGGTTTTCGCCTTT
AAQX01001560_[198-129]	TAGTGACATGCATATTTTAGCCTAGATTTCAGTTACGGAGAGGTTACTAACTGGTAACACGAAAACACATTCTGTTCTAATCAGTGTGAAAACTGGTTTCCGACGTT
AAQX01002784_[626-557]	TAGTGACATGCATATTTTAGCCTAGATTTCAGTTACGGAGAGGTTACTAACTGGTAACACGAAAACACATTCTGTTCTAATCAGTGTGAAAACTGGTTTCCGACGTT

**Utopia-2_PR**	CACATAGCGACTACCAGGTATTTACTACCTGGAAGCCAAGGATTAACCGGTCGGTAATACACATAACTTT
U2 snRNA gene (P.infestans)	--------------------------------ATACCTTCTCGGCCTCTTGGCTAAGATCAAGTGTAGTATCTGTTCTAATCATTGTGAAAACTGGTTTTCGCCTTT
AAQX01001558_[601-532]	CACATAGCGACTACCAGGTAATTACCACCTGGAAGCCAAGGATTAACCGGTCGGTAATACACATAACTTTTCTGTTCTAATCAGTGTGAAAACTGGTTTCCGACGTT
AAQX01001555_[2493-2433]	--------GACTACC-GGTAATTACCACCTGGAAGCCAAGGATTAACCGGTCGGTAATACGCATAACTTTTCTGTTCTAATCAGTGTGAAAACTGGTTTCCGACGTT
AAQX01002538_[669-737]	CACATAGCGACTACCAGGTA-TTACTACCTGGAAGCCAAGGATTAACCGGTCGGTAATACACATAACTTTTCTGTTCTAATCAGTGTGAAAACTGGTTTCCGACGTT
AAQX01002782_[6629-6560]	CACATAGCGACTACCAGGTATTTACTACCTGGAAGCCAAGGATTAACCGGTCGGTAATACACATAACTTTTCTGTTCTAATCAGTGTGAAAACTGGTTTCCGACGTT
AAQX01005908_[820-751]	CACATAGCGACTACCAGGTATTTACTACCTGGAAGCCAAGGATTAACCGGTCGGTAATACACATAACTTTTCTGTTCTAATCAGTGTGAAAACTGGTTTCTGACGTT

**Utopia-3_PR**	GTTAGACATCGGATAGGAACTTCTTAGAAAATTAACGGGTATACCGGATGGTAAATAAAATAAAAACTTC
U2 snRNA gene (P.infestans)	--------------------------------ATACCTTCTCGGCCTCTTGGCTAAGATCAAGTGTAGTATCTGTTCTAATCATTGTGAAAACTGGTTTTCGCCTTT
AAQX01001559_[501-432]	GTTAGACATCGGATAGGAACTTCTTAGAAAATTAACGGGTATACCGGATGGTAAATAAAATAAAAACTTCTCTGTTCTAATCAGTGTGAAAACTGGTTTCCGACGTT
AAQX01003011_[137-68]	GTTAGACATCGGATAGGAACTTCTTAGGAAATTAACGGGTATACCGGATGGTAAATAAAATAAAAACTTCTCTGTTCTAATCAGTGTGAAAACTGGTTTCTGACGTT
AAQX01002785_[675-606]	GTTAGACATCGGATAGGAACTTCTTAGGAAATTAACGGGTATACCGGATGGTAAATAAAATAAACACTTCTCTGTTCTAATCAGTGTGAAAACTGGTTTCCGACGTT

**Utopia-4_PR**	CACATAGCGACTACCAGGTATTTACTACCTGGAAGCCAAGGATTAACCGGTCGGTAATACACATAACTTT
U2 snRNA gene (P.infestans)	--------------------------------ATACCTTCTCGGCCTCTTGGCTAAGATCAAGTGTAGTATCTGTTCTAATCATTGTGAAAACTGGTTTTCGCCTTT
AAQX01001562_[388-319]	CACATAGCGACTACCAGGTATTTACTACCTGGAAGCCAAGGATTAACCGGTCGGTAATACACATAACTTTTCTGTTCTAATCAGTGTGAAAACTGGTTTCCGACGTT
AAQX01005529_[613-544]	CACATAGCGACTACCAGGTATTTACTACCTGGAAGCCAAGGATTAACCGGTCGGTAATACACATAACTTTTCTGTTCTAATCAGTGTGAAAACTGGTTTCCGACGTT
AAQX01005637_[418-487]	CACATAGCGACTACCAGGTATTTACTACCTGGAAGCCAAGGATTAACCGGTCGGTAATACACATAACTTTTCTGTTCTAATCAGTGTGAAAACTGGTTTCCGACGTT

```
Utopia-5_PR                       CACATAGCGACTACCAGGTATTTACTACCTGGAAGCCAAGGATTAACCGGTCGGTAATACACATAACTTT
U2 snRNA gene (P.infestans)       --------------------------------ATACCTTCTCGGCCTCTTGGCTAAGATCAAGTGTAGTATCTGTTCTAATCATTGTGAAAACTGGTTTTCGCCTTT
AAQX01001557_[1145-1076]          CACATAGCGACTACCAGGTATTTACTACCTGGAAGCCAAGGATTAACCGGTCGGTAATACACATAACTTTTCTGTTCTAATCAGTGTGAAAACTGGTTTCCGACGTT
```

***Phytophthora capsici LT1534* (oomycete)**

```
Utopia-1_PCa                      TAGATATCAGATAGGAACCTTCCTTTAGGAAGCTAACGGGTACACTGGATGGTAAATACACATACATTTC
U2 snRNA gene (P.infestans)       --------------------------------ATACCTTCTCGGCCTCTTGGCTAAGATCAAGTGTAGTATCTGTTCTAATCATTGTGAAAACTGGTTTTCGCCTTT
ADVJ01008371_[1869-1800]          TAGATATCAGATAGGAACATTCCTTTGGAAAGCTAACGGGTACACTGGATGGTAAATACACATACATTTCTCTGTTCTAATCAGTGTGAAAACTGGTTTTCCTCATT
ADVJ01008367_[510-441]            TAGATATCAGATAGGAACCTTCCTTTGGAAAGCTAACGGGTACACTGGATGGTAAATACACATACATTTCTCTGTTCTAATCAGTGTGAAAACTGGTTTTCCTCATT
ADVJ01006931_[3500-3567]          TAGATATCAGATAGGAACCTTCCTTTAGGAAGCTAACGGGTACACTGGATGGTAAATACACATACAT-TCTCTGTTCTAATCAGTGTGAAAACTGGTTTTCCTCATT
ADVJ01006928_[1141-1210]          TAGATATCAGATAGGAACCTTCCTTTGGAAAGCTAACGGGTACACTGGATGGTAAATACACATACATTTCTCTGTTCTAATCAGTGTGAAAACTGGTTTTCCTCATT
ADVJ01005502_[942-873]            TAGATATCAGATAGGAACCTTCCTTTGGAAAGCTAACGGGTACACTGGATGGTAAATACACATACATTTCTCTGTTCTAATCAGTGTGAAAACTGGTTTTCCTCATT
ADVJ01005498_[320-251]            TAGATATCAGATAGGAACATTCCTTTAGGAAGCTAACGGGTACACTGGATGGTAAATACACATACATTTCTCTGTTCTAATCAGTGTGAAAACTGGGTTTTCCTCAT
ADVJ01005490_[1150-1081]          TAGATATCAGATAGGAACATTCCTTTAGGAAGCTAACGGGTACACTGGATGGTAAATACACATACATTTCTCTGTTCTAATCAGTGTGAAAACTGGTTTTCCTCATT
ADVJ01009748_[94-25]              TAGATATCAGATAGGAACCTTCCTTTAGGAAGCTAACGGGTACACTGGATGGTAAATACACATACATTTCTCTGTTCTAATCAGTGTGCTCCTT
ADVJ01005488_[641-592]            ------------------CTACCTTTAGGAAGCTAACGGGTACACTGGATGGTAAATACACATACAT-TCTCTGTTCTAATCAGTGTGAAAACTGGTTTTCCTCATT

Utopia-2_PCa                      GTGACATACATATTTTAGGCTACATTTCAGTTACGGAGAGGTTACTAACTGGTA--AATAAAAAGCACTT-T
U2 snRNA gene (P.infestans)       ----------------------------------ATACCTTCTCGGCCTCTTGGCTAAGATCAAGTGTAGTATCTGTTCTAATCATTGTGAAAACTGGTTTTCGCCTTT
ADVJ01005511_[779-711]            GTGACATACATATTTTAGGCTACATTTCAGTTACGGAGAGGTTACTAACTGGTA--AATAAAAAGCACTT-TTCTGTTCTAATCAGTGTGAAAACTGGTTTTCCTCATT
ADVJ01005494_[623-555]            GTGACATACATATTTTAGGCTACATTTCAGTTACGGAGAGGTTACTAACTGGTA--AATAAAAAGCACTT-TTCTGTTCTAATCAGTGTGAAAACTGGTTTTCCTCATT
ADVJ01005492_[2999-2931]          GTGACATACATATTTTAGGCTACATTTCAGTTACGGAGAGGTTACTAACTGGTA--AATAAAAAGCACTT-TTCTGTTCTAATCAGTGTGAAAACTGGTTTTCCTCATT
ADVJ01005514_[941-876]            ---GATATCAGATTTTAGGCTACATTTCAGTTACGGAGAGGTTACTAACTGGTA--AATAAAAAGCACTT-TTCTGTTCTAATCAGTGTGAAAACTGGTTTTCCTCATT
ADVJ01007874_[1163-1216]          GTGACATACATATTTTAGACTACATCTCAGTTACGGAGAGGTTACTAACTGGTAGTAAATACGAACACATATTCTGTTCTAATCAGTGTAAAAACTGGTTTTCCTCATT

Utopia-3_PCa                      TAAATAGGGACTACCAGGTAATTACTACCTGGAAGCACAGGATTAACCTTTTGGTAATACACATAATTTC
U2 snRNA gene (P.infestans)       --------------------------------ATACCTTCTCGGCCTCTTGGCTAAGATCAAGTGTAGTATCTGTTCTAATCATTGTGAAAACTGGTTTTCGCCTTT
ADVJ01008819_[235-166]            TAAATAGGGACTACCAGGTAATTACTACCTGGAAGCACAGGATTAACCTTTTGGTAATACACATAATTTCTCTGTTCTAATCAGTGTGAAAACTGGTTTTCCTCATT
ADVJ01008816_[657-588]            TAAATAGGGACTACCAGGTAATTACTACCTGGAAGCACAGGATTAACCTTTTGGTAATACACATAATTTCTCTGTTCTAATCAGTGTGAAAACTGGTTTTCCTCATT
ADVJ01005507_[2677-2608]          TAAATAGGGACTACCAGGTAATTACTACCTGGAAGCACAGGATTAACCTTTTGGTAATACACATAATTTCTCTGTTCTAATCAGTGTGAAAACTGGTTTTCCTCATT
ADVJ01003864_[253-194]            TAAATAGGGACTACCAGGTAATTACTACCCGGAAGCACAGGATTAAC----------CACACATAATTTCTCTGTTCTAATCAGTGTGAAAACTGGTTTTCCTCATT
```

***Pythium ultimum DAOM BR144* (oomycete)**

```
Utopia-1_PU                       GTGACATGCATATTCAAACCGATACATATTCGTTAGGAGAGGTTACTAACTGGTAATATATCACCATTTC
U2 snRNA gene (P.infestans)       --------------------------------ATACCTTCTCGGCCTCTTGGCTAAGATCAAGTGTAGTATCTGTTCTAATCATTGTGAAAACTGGTTTTCGCCTTT
ADOS01001692_[6503-6572]          GTGACATGCATATTCAAACCGATACATATTCGTTAGGAGAGGTTACTAACTGGTAATATATCACCATTTCTCTGTTCTAATCAGTGTGAAAACTGATTACCGGGTGA
ADOS01001321_[21992-22061]        GTGACATGCATATTCAAACCGATACATATTCGTTAGGAGAGGTTACTAACTGGTAATATATCACCATTTCTCTGTTCTAATCAGTGTGAAAACTGATTACCGGGTGA
ADOS01000638_[3343-3274]          GTGACATGCATATTCAAACCGATACATATTCGTTAGGAGAGGTTACTAACTGGTAATATATCACCATTTCTCTGTTCTAATCAGTGTGAAAACTGATTACCGGGTGA
```

Utopia-2_PU
The 3' terminus is uncertain.

***Saprolegnia parasitica CBS 223.65* (oomycete)**

```
Utopia-1_SaPa                     GAACTGTCCACTGAATCTGGACTTGGTTAAATCACGATAGCCTAGCTTGACCGTGACACTGAAGACACTT
U2 snRNA gene (P.infestans)       --------------------------------ATACCTTCTCGGCCTCTTGGCTAAGATCAAGTGTAGTATCTGTTCTAATCATTGTGAAAACTGGTTTTCGCCTTT
Supercontig_2.73_[45192-45123]    GAACTGTCCACTGAATCTGGACTTGGTTAAATCACGATAGCCTAGCTTGACCGTGACACTGAAGACACTTTCTGTTTTAATCAGTGTGAAAACTGATTTCCACACTT
```

***Saprolegnia diclina* (oomycete)**

Utopia-1_SaDi
The 3' terminus is uncertain.

***Hyaloperonospora arabidopsidis* (oomycete)**

Utopia-1_HAra
The 3' terminus is uncertain.

***Pseudoperonospora cubensis* (oomycete)**

Utopia-1_PCu
The 3' terminus is uncertain.

***Nasonia vitripennis* (jewel wasp)**

```
Utopia-1_NVit                     AAGCAGGCCAAGCGCCGTGTTCTA-ACCCCGTTTTAGGGGAAGTTACTTAACCTAAAAA-TACAACTTTTCC
U2 snRNA gene (fruit fly)         ---------------------------------ATCGCTTCTCGGCCTTATGGCTAAGATCAAAGTGTAGTATCTGTTCTTATCAGCTTAACATCTGATAGTTCCTCCA
NW_001815525_[20724-20655]        AAGCAGGCCAAGCGCCGTGTTCTA-ACCCCGTTTTAGGGGAAGTTACTTAACCTAAAAA-TACAACTTTTCCTCTGTTCTTATCAGCTTGATATCTGATACGCTCCTCA
NW_001815525_[27075-27006]        AAGCAGGCCAAGCGCCGTGTTCTA-ACCCCGTTTTAGGGGAAGTTACTTAACCTAAAAA-TACAACTTTTCCTCTGTTCTTATCAGCTTGATATCTGATACGCTCCTCA
NW_001815525_[29381-29312]        AAGCAGGCCAAGCGCCGTGTTCTA-ACCCCGTTTTAGGGGAAGTTACTTAACCTAAAAA-TACAACTTTTCCTCTGTTCTTATCAGCTTGATATCTGATACGCTCCTCA
NW_001815255_[16955-16895]        AAGCAGGCCAAGCGCCGTGTTCAC-ACCCCGTTTTAGGGGAAGTTACTTAACATAAAAA-TACCTAATTTCCTCTGTTCTTATCAGCTTGATATCTGATACGCTCCTCA
NW_001815170_[348567-348619]      -AGCAGGCCAAGCGCCATGTTTTC-ACCCCGTTCCAGGGACAGT--CCTAATTTAAA---TGAAAGAATTCCTCTGTTTTTATCAGCTTGATATCTGATACGATTCTCA
NW_001815525_[24769-24700]        AAGCAGGCCAAGCGCCATGTTCTA-ACCCCGTTTTAGGGGAAGTTACTTAACCTAAAAA-TACAACTTTTCCTCTGTTCTTATCAGCTTCTCGGCCTATTGGCTAAGAT
NW_001815525_[17981-17922]        -AGCAGGCCAAGCGCCGTGTTCAC-ACCCCGTTTTAGGGGAAGTTACTTAACATAAAAA-TACCTAATTTCCTCTGTTCTTATCAGCTTGATATCCTCATTGAGGAGCC
NW_001820416_[1109972-1110033]    AAGCAGGCCAAGCGCCGTGTTCTA-ACCCCGTTTTAGGGGAAGTTACTTAACATTAAAAATACCTAATTTCCTCTGTTCTTATTACGAAGAGAAGGCCTGCACCGATCC
NW_001820416_[1108636-1108575]    AAGCAGGCCAAGCGCCGTGTTCTA-ACCCCGTTTTAGGGGAAGTTACTTAACATTAAAAATA---CCATTCCTTCGTACGGGTTTTCGTGCCGGCATAGCCGGGTGGGA
Utopia-1_NVit                     -----------------------------------------------------------------CCATTCCTTCGTACGGGTTTTCGTGCCGGCATAGCCGGGTGGGA
```

***Nasonia longicornis* (jewel wasp)**

```
Utopia-1_NVit                     AAGCAGGCCAAGCGCCGTGTTCTA-ACCCCGTTTTAGGGGAAGTTACTTAACCTAAAAA-TACAACTTTTCC
U2 snRNA gene (fruit fly)         ---------------------------------ATCGCTTCTCGGCCTTATGGCTAAGATCAAAGTGTAGTATCTGTTCTTATCAGCTTAACATCTGATAGTTCCTCCA
ADAP01198410_[242-183]            -AGCAGGCCAAGCGCCGTGTTCTA-ACCCCGTTTTAGGGGAAGTTACTTAACATAAAAA-TACCTAATTTCCTCTGTTCTTATCAGCTTGATATCTGATACGCTCCTCA
ADAP01022836_[929-988]  -         AGCAGGCCAAGCGCCGTGTTCTA-ACCCCGTTTTAGGGGAAGTTACTTAACATAAAAA-TACCTAATTTCCTCTGTTCTTATTA
ADAP01123373_[142-198]  -         AGCAGTCCAAGCGCCATGTTTTTCACCTCGTTTTAGGGGAAGTTA-TTATTATAAAAA---CGACGATTCCTTCGTACAAGTTTT-GTGCCGGCATAGCCGGGTAGGG
Utopia-1_NVit                     -----------------------------------------------------------------CCATTCCTTCGTACGGGTTTTCGTGCCGGCATAGCCGGGTGGGA
```

***Nasonia giraulti* (jewel wasp)**

```
Utopia-1_NVit                     AAGCAGGCCAAGCGCCGTGTTCTA-ACCCCGTTTTAGGGGAAGTTACTTAACCTAAAAA-TACAACTTTTCC
U2 snRNA gene (fruit fly)         ---------------------------------ATCGCTTCTCGGCCTTATGGCTAAGATCAAAGTGTAGTATCTGTTCTTATCAGCTTAACATCTGATAGTTCCTCCA
ADAO01172112_[925-985]            AAGCAGGCCAAGCGCCGTGTTCTA-ACCCCGTTTTAGGGGAAGTTACTTAACATAAAAA-TACCTAATTTCCTCTGTTCTTATTA
ADAO01155664_[74-130]             -AGCAGTCCAAGCGCCATGTTTTTCACCTCGTTTTAGGGGAAGTTA-TTATTATAAAAA---CGACGATTCCTTCGTACAAGTTTT-GTGCCGGCATAGCCGGGTGGGG
Utopia-1_NVit                     -----------------------------------------------------------------CCATTCCTTCGTACGGGTTTTCGTGCCGGCATAGCCGGGTGGGA
```

***Ganaspis* sp. G1 (parasitoid wasp)**

```
Utopia-1_GG1                      GTATATTACCCAGCAGGGAGCTTGTCCCCTGACTGGGAACATTTTCTTTTTTTTGAAAATAAACGTCTTA
U2 snRNA gene (fruit fly)         -------------------------------ATCGCTTCTCGGCCTTATGGCTAAGATCAAAGTGTAGTATCTGTTCTTATCAGCTTAACATCTGATAGTTCCTCCA
GAIW01019287_[92-23]              GTATATTACCCAGCAGGGAGCTTGTCCCCTGACTGGGAACATTTTCTTTTTTTTGAAAATAAACGTCTTATCTGTTCTTATCAGCTTGATAT
```

***Megachile rotundata* (alfalfa leafcutter bee)**

```
Utopia-1_MRo                      CTGAGAAATTTATGTATCACCCTGCAGCTTCGGCTGTGGGGAAACATTCATTGCTAAATAAACTTCAATTTA
U2 snRNA gene (fruit fly)         ---------------------------------ATCGCTTCTCGGCCTTATGGCTAAGATCAAAGTGTAGTATCTGTTCTTATCAGCTTAACATCTGATAGTTCCTCCA
AFJA01010887_[2214-2145]          CTGAGAAATTTATGTATCACCCTGCAGCTTCGGCTGTGGGGAAACATTCATTGCTAAATAAACTTCAATTTATCTGTTCTTATCAGCTTAATATCTGATACACTCCCCA
AFJA01013412_[221-154]            --GAGAAATTTATGTATCACCCTGCAGCTTCGGCTGTGGGGAAACATTCATTGTTAAATAAACTTCAATTTATCTGTTCTTATCAGCTTAATATCTGATACACTCCCCA
AFJA01003025_[614-556]            -----------ATGTACCACCCTGCAGCTTCGGCTGTGGGGAAACATTCATTGTTAAATAAACTTCAATTTATCTGTTCTTATCAGCTTAATATCTGATACACTCCCCA
AFJA01011480_[625-557]            -TAAGAGATTTATGTATCACCCTGCAGCTCCGGCTGTGGGGAAACATTAATTGAAAAATAAACTCCAATTTATCTGTTCTTATCAGCTTAATATCTGATACACTCCCCA
AFJA01013089_[21-88]              --GAGAAATCTATGTATCACCCCGTAGCCTCGGCTGTGGGGAAACATGTTTTGAAAAATAAACTCCAATT-ATCTGTTCTTATCAGCTTAATATCTGATACACTCCCCA
AFJA01016432_[625-682]            -----------ATGTATCACCCTGCAGCTTCGGCTGTGGGGAAACATTAATTGAAAA-TAAACTCCAATTTATCTGTTCTTATCAGCTTAATATCTGATAC
AFJA01016501_[90-32]              -----------ATGTATCACCCTACAGCTTCGGCTGTGGGGAAACATTCAGTGAAAAATAAAGTCCAATTTATCTGTTCTTATCAGCTTAATATCTGATAC
AFJA01003025_[2330-2280]          -----------ATGTATCACCCCGCAGCTTCGGCTGTGGGGAAACATTAACTGAAAAATAAAAATTCGTCCTCGATCTCCTCCGAATTTCCCCACGATATGCCTCACTC
```

***Lasioglossum albipes* (bee)**

Utopia-1_LAl
The 3' terminus is not sequenced.

Solenopsis invicta **(red imported fire ant)**

**Utopia-1_SIn**	CTTTGACTGTAWTGACCCCAGGGGGTACTGTCCCCTGGGGAAAAGCTTTT-GATTAATAAAGCGTGCTTAA
U2 snRNA gene (fruit fly)	--------------------------------ATCGCTTCTCGGCCTTATGGCTAAGATCAAAGTGTAGTATCTGTTCTTATCAGCTTAACATCTGATAGTTCCTCCA
AEAQ01028046_[1438-1507]	CTTTGACTGTATTGACCCCAGGGGGTACTGTCCCCTGGGGAAAAGCTTCT-GATTAATAAAGCGTGCTTAATCTGTTCTTATCAGCTTAATATCTGATACAGTCCCCA
AEAQ01004819_[1707-1776]	CTTTGATTGTAACGACCCCAGGGGGTACTGTCCCCTGGGGAAAAGCTTTT-GATGAATAAAGCGTGCTTAATCTGTTCTTATCAGCTTAATATCTGATACAGTCCCCA
AEAQ01083385_[359-428]	CTTTGATTGTAACGACCCCAGGGGGTACTGTCCCCTGGGGAAAAGCCTTT-GATGAATAAAGCGTGCTTAATCTGTTCTTATCAGCTTAATATCTGATACAGTCCCCA
AEAQ01083008_[1070-1140]	CTTTGACTGTATTGACCCCAGGGGGTACTGTCCCCTGGGGAAAAGCTTTTTGATTAATAAAGCGTGCTTAATCCGTTTGGTCCAAATCTGCGGAAAACCTCTTCTATT
AEAQ01010025_[1647-1580]	CTTTGACTGTAATGACTCCAGGGGGTACTGTCCCCTGGGGAAAAGCTTCT-GATTAATAAAGCGTGCTTTATTTACAAATCATTAAGGCGACATAAGACCATGTTGTA
AEAQ01029229_[83-152]	CTTTGACTGTATTGACCCCAGGGGGTACGTCCCCTGGGGAAAAGCTTTTT-GATTAATAAAGCGTGCTTAATCCGTTTGGTCCAAATCTGCGGAAAACCTCTTCTATT

Acromyrmex echinatior **(leaf-cutting ant)**

**Utopia-1_AEc**	GTTTGTATTACCCCAGAGGGGAATTGTCCCTCTGGGGAAAAAAAATGATTGGAAAAATAAAGTGAGCTAA
U2 snRNA gene (fruit fly)	-------------------------------ATCGCTTCTCGGCCTTATGGCTAAGATCAAAGTGTAGTATCTGTTCTTATCAGCTTAACATCTGATAGTTCCTCCA
AEVX01016289_[542-479]	-TTTGTATTACCCCAGAGGGGAAGGTCCCTCTGGGGAAGATGTTTGGGACAA-----TAAAGTGAGCTAATCTGTTCTTATCAGCTTAATATCTGATACAGTCCCCA
AEVX01008733_[11980-11915]	-TTTGTATTACCCCAGAGGGGAATTGTCCCTCTGGGGAAAATAATTGAGAAAAA---TAAAGTGAGCTAATCTGTTCTTATCAGCTTAATATCTGATACAGTCCCAA
AEVX01010371_[11632-11568]	GTCTGTATTACCCCAGAGGGGAATTGTCCCTCTGGGGAAAATGATTGAAAAA-----TAAAGTGAGCTAATCTCTTTTTACTAATATTATTATATAATATATTATAT
AEVX01004272_[31881-31946]	GTCTGTATTACCCCAGAGGGGAATTGTCCCTCTGGAGCAAATGATTGAAAAAA----TAAAGTGAGCTAATCCTTTTTTACTAATATTCCTATTGAGCTAGCATTAA
AEVX01013037_[116-180]	GTTTGTATTACCCCAGAGGGGAAATGTCCCTCTGGGGCAAATGATTGAAAAA-----TAAAGTGAGCTAATTAGTTCGTCACAGTTTAATCAAACTATAATAAAAAA
AEVX01002336_[417-481]	GTTTGTATTACCCCAGAGGGGAAATGTCCCTCTGGGGCAAATGATTGAAAAA-----TAAAGTGAGCTAATTAGTTCGTCACAGTTTAATCAAACTATAATAAAAAA
AEVX01007924_[35336-35399]	GTCTGTATTACCCCAGAGAGGAATTGTCCCTCTGGGGAAAATGATTGAAAAA-----TAAAGTGAGTTATCCCTTTTTAATATTCCCTTTTTAATATTCCTATTGAG
AEVX01014258_[2014-2079]	-TTTGTATTACCCCAGAGGGGAATTGTCCCACTGGGAAAGATAACTGAGAAAAA---TAAAGTGAGCTAATCCCTTTTTACTAATATTCCTGTTAAGCTAGCATTAA
AEVX01013275_[3971-3906]	-TTTGTATTACCCCAGAGGGGAATTGTCCCACTGGGAAAGATAACTGAGAAAAA---TAAAGTGAGCTAATCCCTTTTTACTAATATTCCTGTTAAGCTAGCATTAA
AEVX01006095_[1-65]	-TCTGTATTACCCCAGAGGGGAATTGTCCCTCTGGGGAAGATAATTGAGAAAAA---TAAAGTCTGCTACGCGGGGAAGGTCATCCAAGAAATTCTGCCACCTGTCT
AEVX01005487_[4407-4346]	--TTGTATTACCCCAGAGGGGAAATGTCCCTCTGGGGCACATGATTGAAAA------TAAAGTGAGTTAATTTGTAATATTATTTTTCTAAATTATAACTAAAAAAA
AEVX01012669_[49768-49706]	--TTGTATCACCCCAGAGGGGGAATGTCCCTCTGAGGAAAAAGATTGATGAA-----TAAAGTGAGCTAATCTGTTATTTTTTAGCTTTGATAGACGTACTATGGTG
AEVX01008327_[1762-1701]	--TTGTAATACCTCAGAGGGGAAATGTCCCTCTGGGGAAGAAGCCTGAAAA------TAAAGCGAGCTAATCTGTTAAAAAAAAACAATTTGTTAACAACCTTTTGA
AEVX01003492_[82451-82385]	-TCTGTATTATCCCAGAGGGGAAGTGTTCCCTCTGAGGAAGATTTTTGATGAAA--TAAAAGCGAGCTAATTCTAAACCTTTTTTACCCGATCCACAGATTGCGTCA

Atta cephalotes**(leaf-cutting ant)**

**Utopia-1_AEc**	GTTTGTATTACCCCAGAGGGGAATTGTCCCTCTGGGGAAAAAAAATGATTGGAAAAATAAA----------GTGAGCTAA
U2 snRNA gene (fruit fly)	---ATCGCTTCTCGGCCTTATGGCTAAGATCAAAGTGTAGTATCTGTTCTTATCAGCTTAACATCTGATAGTTCCTCCA
ADTU01020076_[11381-11317]	-TTTGTATTACCCCGGAGGGCA--TGTCCCTTTGGGGAAGTCATTTGAGAAAAATAAAA------------GTGAGCTAATCTGTTCTTATCAGCTTAATATCTGATACAGTCCCCA
ADTU01020074_[5970-5910]	-TTTGTATTATCTCAGAGAGAAAATGTCATTCTGGGGAAATATTTTGAAAAATAAAAGCTAATTTTATTTTTCAAGCTAATCTGTTCTTATCAGTTTCATATCTGATACAGTTTCCA
ADTU01002675_[6096-6037]	-TTTGTATTATCCCAGAAAGGAAATATCTCTCTGGGGAA----TTTGAAAATAAAAA---------------CGAGCTAATCTGTTCTTATATTGAAATGAGTATGTAGTCATGGAG
ADTU01013138_[6651-6592]	-----TATTACCCGAGAGAGGAAATGTCCCTCTGGGGAAGATATTTGAAAATAAAA---------------GTGAGCTAATATATTTGTTACTTATCTCTGTGACGGTCAGTGGTCC
ADTU01021008_[17388-17449]	-TTTGTATTATCCCAGAGGGGA--TGTCCCTCTGGGGAAGTCATTTGAGAAAAATAAAA------------GTTAGCACTTTCCACCATGGAAGCGGAGTTCATTGCACTAGCGGTA

Utopia-1_ACep
The 3' terminus is not sequenced.

Pogonomyrmex barbatus **(red harvester ant)**

**Utopia-1_PBa**	CTTATGTACCCACCCCGGAGGGTGAGATGCCCTCCGGGGAACATTAATTT-AAAATAAAAGTGAGCTAA
U2 snRNA gene (fruit fly)	------------------------------ATCGCTTCTCGGCCTTATGGCTAAGATCAAAGTGTAGTATCTGTTCTTATCAGCTTAACATCTGATAGTTCCTCCA
ADIH01013170_[382-318]	-TTATGTATCCACCCCGGAGGGTGAGATGCCCTCCGGGGAACATTC-TTT-AAAATAAAAGTGAGCTAATCTGTTCTTATCAGCTTAATATCTGATACAGTCTCCA
ADIH01013824_[9626-9690]	-TTATGTATCCACCCCGGAGGGTGAGATGCCCTCCGGGGAATATTC-TTT-AAAATAAAAGTGAGCTAATCTGTTCTTATCAGCTTAATATCTGATACAATCTCCA
ADIH01006726_[1189-1125]	-TTGTGTATCCACCCCGGAGGGTGAGATGCCCTCCGGGGAACATTA-GTT-AAAATAAAAGTGTGCTAATCTGTTCTTATCAGCTTAATATCTGATACAGTCTCCA
ADIH01004088_[1102-1165]	--TGTGTATACACCCCGGAGGGTGAGATGCCCTCCGGGGAACATTA-TTT-AAAATAAAAGTGTGCTAATCTGTTCTTATCAGCTTAATATCTGATACAGTCTCCA
ADIH01004156_[140-204]	--TGTGTATACACCCCGGAGGGTGAGATGCCCTCCGGGGAACATTAATTTTAAAATAAA-GTGAGCTAATCTGTTCTTATCAGCTTAATATCTGATACAGTCTCCA
ADIH01004101_[327-263]	--TGTGTATACACCCCGGAGGGTGAGATGCCCTCCGGGGAACATTAATTTTAAAATAAA-GTGAGCTAATCTGTTCTTATCAGCTTAATATCTGATACAGTCTCCA
ADIH01004141_[399-336]	-TTATGTATCCACCCCGGAGGGTGAGATGCCCTCCGGGGAACATTA-GTT-AAAATAAA-GTGTGCTAATCTGTTCTTATCAGCTTAATATCTGATACAGTCTCCA
ADIH01018466_[9664-9603]	----TGTATCTACCCCGGGGGGTGAGATGCCCTCCGGGGAACATTC-TTT-AAAATAAAAGTGTGCTAATCTGTTCTTATCAGCTTAATATCTGATACAGTCTCCA
ADIH01004090_[341-277]	CCTGTGTATCTACCCCGGAGGGTGTGATGCCCTCCGGGGAACATTG-CTT-AAAATAAA-GTGTGCTAATCTGTTCTTATCAGCTTAATATCTGATACAGTCTCCA
ADIH01004093_[1140-1079]	----TGTATCTACCCCGGGGGGTGAGATGCCCTCCGGGGAACATTC-TTT-AAAATAAAAGTGTGCTAATCTGTTCTTATCAGCTTAATATCTGATACAGTCTCCA
ADIH01019771_[9796-9728]	CTTATGTACCCACCCCGGAGGGTGAGATGCCCTCCGGGGAACATTA-TTT-AAAATAAAAGTATGCTAGCTCACTGCGGTAACACGAAATAAAATTTAATAAGCTG
ADIH01014020_[13611-13544]	--TATGTATCCACCCCGGAGGGTGAGATGCCCTCCGGGGAATATTAATTT-AAAATAAAAGTGAGTTAATTCATTTAAAGTGATGTAATTAAATGAAATATAGGAT
ADIH01023609_[369-429]	CTTATGTATCCACCCCGGAGGGTGAGATGCCCTCCGGGGAACATTA-TTT-AAAATAAAAGTGG-----CTTCAATGTCCCCTACAATCAGCGTTAAAATTATCAA
ADIH01006667_[853-917]	-TTATGTATCCACCCCGGAGGGTGAGATGCCCTCCGGGGAACATTC-TCT-AAAATAAAAGTGAGCTAATATTAATATGTTTAAATAATTAACTAAAAATTACACT
ADIH01023608_[1240-1303]	--TATGTATCCACCCCGGAGGGTGAGATGCCCTCCGGGGAACATTA-TTT-AAAATAAAAATGTGCTAATCTGTTTTTCCCTGAAGGAACACAATGTCACGGATAC
ADIH01004128_[1038-1103]	CTTATGTATCCATCCCGAAGGGTGAGATGCCCTCCGGGGAACATTC-TCT-AAAATAAAAGTGAGCTAATACTAATATGTTTAAATAATTAACTAAAAGTTACACT
ADIH01021822_[2040-2105]	CTTTTGTATCTACCCCGGGGAGTGAGATGCCTTCCGGGAAACATTAATTTTAAA-TAAA-GTGAGCTAATCTATATTGTATGCATTTACATTCTTTTAATTAAGAA

Harpegnathos saltator **(Jerdon's jumping ant)**

**Utopia-1_HSal**	CTATGCCGGAATATACATTGTAATTACCCCGGGGGGCATGCCCCTCGGGGAACATTTATTGC-TAAATAAA
U2 snRNA gene (fruit fly)	--------------------------------ATCGCTTCTCGGCCTTATGGCTAAGATCAAAGTGTAGTATCTGTTCTTATCAGCTTAACATCTGATAGTTCCTCCA
AEAC01026118_[526-596]	CTATGCCTGAAAATATACTGTATTTACCCCGGGGGGCATGCCTCCCGGGGAACATTTATTGTGTAAATAAATCTGTGAGTTATCTGTGTTTGCAATCTGTGTCCCCCT
AEAC01006728_[28053-28123]	CTATGCCTGAAATAATTCTGTATTTATTCCGGGGGGCATGCCCCTCGGGGAACATTTATTGTCTAAATAAAAGTATTCGAAGATTAAAGTTTTCCA
AEAC01025536_[1653-1585]	CTAGGCCTGAAATAATATTGTAATTACCCCGGGGGGCTTGTCCCTCGGGGAACATTTATTG--AAAATAAATTCGCAACTAATATATAGTATAATATATAGTATAGTA
AEAC01015619_[271-204]	CTATGCCTGAAAATATACTGTATTTACCCCGGGGGGCATGCCTCCCAGGGAACATCTATTGTGTAAATGTAGAAAATGTAGGGCTTTGAGAGGACTTTATTTTGGTAC
AEAC01009535_[1670-1600]	CTATGCTTGAAATAATTTTGTATTTACTCAGGGGGGCATGCCCCTCGAGGAACATTTATTGTCTAAATAAAAATGTACTAATAGGACTTTATTTTGATGCCAGAAACA
AEAC01023855_[409-462]	-----------------TTGTATTTACTCCGGGGGGCATGCCCCTCGGGGAATATTTATTGTCTAAATAAAGTATCGAAGGTTAAAGTACTTTTCGGCCTACCTTTGG
AEAC01020622_[7166-7097]	CTGTGCCTGATACGATATTGCAATTACCCCGGGGGACTTGTCCCCCGGGGAACATTTATTGA-TAAATAAATTGGAACGTATGTGTGAATCTTTTTTGAAACTTTTGT
AEAC01001290_[108-48]	CTATGCCTGAAATAATTTTGTATTTACTCCGGGGGGCATGCCCCTCGAGGAACATTTATTGTCGATATCCAGTATTCCGTCGCGTACTTTAAGGCCTACCTTTGGGTA
AEAC01015029_[107-38]	CTATGCCTGAAGAGATGTTGTATTTACCCCGGGGAGCAAGCCCTCCGAGGAACATTTATTGT-AAAATAAAGTTAAAGGATCTCCGCTTCTACTGGGAGTAGAGAGTC
AEAC01024804_[814-883]	CTAGGCCTGAATATACACTGTAATTACCCCAGGGGTCTTGTCCCCCGGGGAACATTAATTCG-TGAATAAACGTGCAACTAACTGTTCGAAATACCTTCCTCCAGCAT
AEAC01023649_[535-603]	CTATACCTATAATAATACTGTATTTACCCCGGGGGGTTTGCCCCGGGG--AACATTTATTGTTTAAATAAAGTCTTTCTCCGTTGATATCTCCGCTTCTACTGGGAGT
AEAC01018547_[1010-1080]	CTATGCCTGTAACAGTGTTGTATATACCCCAGGGGGCAAGCCCCCCGGAGAACATTTATCGTTTAAATAAAGTTTTATGTCGAAGGTTAAAGTTTTCCAAGATTTCT
AEAC01017254_[43395-43444]	----------------------CCCCGGGGGGCTTATGTAAGCCCCACGGGGAACATTTTGTTTAAATAAAGTTTTAAATATTGTCTAAGGTTAAAGTTTTCCAACAT

Camponotus floridanus **(Carpenter ant)**

**Utopia-1_CFl**	CCGTGACTCTTGTACCTGCACCCCGGGGGG-TCTGTCCCCGGGG--AACATTAATTGAAATAAAA
U2 snRNA gene (fruit fly)	-----------------------------ATCGCTTCTCGGCCTTATGGCTAAGATCAAAGTGTAGTATCTGTTCTTATCAGCTTAACATCTGATAGTTCCTCCA
AEAB01000713_[5718-5648]	CCGTAATTCTTGTACCTGCACCCCGGGGAG-TTTGTCTCCTGGGG-AACATTAATTGAAAATAAA---GGCAATTATTTTCTCGCGAACCATCCGATGGTAGAAA
AEAB01024608_[268-206]	--------CTTGTATTTGCATCCGGGGGAG-TCTGTCCCTCGAGG-AACATTGATTGAAATAAAA---GGCAATTAATTATCTTATTTTGCTTTTTCCCATCTTT
AEAB01018037_[1340-1296]	-----------------------GGGGGGG--TTGTCCCCCGGGG-AACATTAATTGAAAATAAA---GGCAATATATTCCATGCAATTTTTCATACTCAAAATA
AEAB01019243_[4886-4939]	CCGTAACTCTTGTACCTGCACTCCGGGGGG-TTTGTCCCCGGGG--AACATTAATTG----TA-----TGGCGTCGTTTGGTTGCATGTTCGTTCAATCTTAGTC
AEAB01013137_[1456-1515]	---TGATTTATGTGCCTGCACCCCGGGGGG-TCTGTCCCCCGGGG-AACATTATTTAAAAATAAA---GTGGCTAATTTACATCCAACACTTCTTCTTTCAATGT
AEAB01017439_[783-843]	---TGACTATGTATTTGCACCCCGGGGGGGGCCTGTTCCCCGGGG-AACATTAGTTGAAATAAAAAA-TTATTCCACACAATTTTTTGTCGCTATACTCAAAATA
AEAB01031512_[510-578]	---TGACTGTATATCAGCACCCCGGGGGGG-CCTATCCCTCCGGGGAACATTAATTGACAATAAAAA-TTAATTCCACACAATTTCTCATACTCAAAATATTCCA
AEAB01000539_[4174-4121]	-----------TATCAGCACCCCGGGGGGG-CCTGTCCCCCCGGGGAACATTAATTGAAAATAAAAAATTTATTCCACACAATTTCTCATACTCAAAATAAACCA
AEAB01029477_[637-697]	---TGACTATATATTTGCACCCCGGGGAGG-CCTGTCCCCCGGGG-AACATTAGTTGAAAATAAAAAATATTGGACCAATAAATCAATTTAATTTTTAACCAATA

Drosophila yakuba **(fruit fly)**

**Utopia-1_DYak**	CACAATAACACTCACCCGGCCTGCCCCAGAGGCAGGTAAACATTTACTGGCCATATGGCTTTTTTTTTAA
U2 snRNA gene (fruit fly)	---------------------------------ATCGCTTCTCGGCCTTATGGCTAAGATCAAAGTGTAGTATCTGTTCTTATCAGCTTAACATCTGATAGTTCCTCCA
chr2R_[6281223-6281156]	CACAATAACACTCACTTGGCCTGCCCCAGAGGCAGGTAAACATTTACTGGCCATATGGCTTTTTTTTTTAAATCTGTTCTTATCAGCTTAACATCTGATAGTTCCTCCA
chr2L_[8596472-8596406]	CACAATAACACTCACTTGGCCTGCCCCAGAGGCAGGTAAACATTTACTGGCCATATGGCTTTTTTTTATA--TCTGTTCTTATCAGCTTAACATCTGATAGTTCCTCCA
chr2L_[11102622-11102679]	-------------ACCCGGCCTGCCCCAGAGGCAGGTAAACATTTACTGGCCATATGGCTAATTTTTTTAA-TCTGTTCTTATCAGCTTAACATCTGATAGTTCCTCCA
chr2L_[9653799-9653865]	-ACAATAGCGCTCACCCGGCCTGCCCCAGAGGCAGGCAAACATTTACTGGCCATATTATGGTTATTTTAAA-TCTGTTCTTATCAGCTTAACATCTGATAGTTCCTCCA

Heliconius melpomene melpomene **(Postman butterfly)**

**Utopia-1_HMM**	TGTAAAATTAAACTTTATATTTGTGTTGTATCTTT-AGAATACAGAGTTAATAATTATAAATATA
U2 snRNA gene (fruit fly)	--------------------------ATCGCTTCTCGGCCTTATGGCTAAGATCAAAGTGTAGTATCTGTTCTTATCAGCTTAACATCTGATAGTTCCTCCA
CAEZ01003146_[6463-6394]	TGTAAAATTAAACTTTATATTTGTGTTGTATCTTT-AGAATACAGAGTTAATAATTATAAATATAGTTTATATGTGGGCCGATCCCGGCGGCACTGCAATGC
CAEZ01003147_[1579-1642]	TGTAAAATTAAACTTTATATTTGTGGTGTATCTTT-AGAATACAGAGTTAATAATTATAAATATAAATAAATACAGATAGAATCATAGTTAAACCGTCATCA
CAEZ01009982-[107828-107767]	TGTATAATTAAACTTTATATTTATTTTGTATCTTT-AGAATACAGAGTTAATAAATATAAATAAATACAGATAGAATCATAGTTAAAGTGTTATCATTCAGA
CAEZ01003605_[793-854]	TGTATAATTAAGCTTTATATTTATTTTATATCTTT-AGAATACAGAGTTAATAAATATAAATAAATACAGATACAGATAGAATCATAGTTAAACCGTCATAA
CAFA01011426_[3175-3239]	TGTATAATTAAACTTTTTTATTTATTTTGTATTTTTAGAATACAGAGTTAATAATTATAAATATAAATAAATACAGATAGAATCATAGTTAGAGTCATCATT

***Chrysopa pallens* (lacewing)**

Utopia-1_CPa
The 3' terminus is not sequenced.

***Dendroctonus ponderosae* (mountain pine beetle)**

```
Utopia-1_DPo                   CAAAAACTCTTATACACCACTGTTGTAATACTCTTTTCTTTT---CTTTGTCAA-GCATTCGCTGAATAAACTA
U2 snRNA gene (fruit fly)      -------------------------------------ATCGCTTCTCGGCCTTATGGCTAAGATCAAAGTGTAGTATCTGTTCTTATCAGCTTAACATCTGATAGTTCCTCCA
APGL01015132_[1167-1106]       CAAGAGCTCTTATTCATCACTGTTCTAATACTTTT------------CTATCAAGGCATTCGCTAAATAAACTA--TCTGTTCTTATCAGCTTAATATCTGATACGCCCCGCA
APGK01019509_[9015-8954]       CAAGAGCTCTTATTCATCACTGTTCTAATACTTTT------------CTATCAAGGCATTCGCTAAATAAACTA--TCTGTTCTTATCAGCTTAATATCTGATACGCCCCGCA
APGL01002790_[705-766]         CAAGAGCTCTTATTCATCACTGTTCTAATACTTTT------------CTGTCAAAGCATTCGCTAAATAAACTA--TCTGTTCTTATCAGCTTAATTGCGTGAAACTTTGTGT
GAFX01020405_[79-10]           CAAAAACTCTTATACACCACTGTTGTAATACTCTTTTCTTTT---CTTTGTCAA-GCATTCGCTGAATAAACTATATCTGTTC
APGK01020568_[98-170]          CAAAAACTCTTATCCACCACTGTTATAATACCTTTTTTTTTTTTCTTTGTCAA-GCATTCGCTAAATAAACTATATCTGTTTTGTGCAGTTTGTGTCCCCAAGTTCCAGCAT
APGL01020145_[1868-1807]       CAAGAGCTCTTATTCATCACTGTTCTAATACTTTT------------CTGTCAAAGCATTCGCTAAATAAATTATACTGGCGGAGGAAACTTTATTTGCCACAGATGGTTCCG
APGK01045337_[2795-2734]       CAAGAGCTCTTATTCATCACTGTTCTAATACTTTT------------CTGTCAAAGCATTCGCTAAATAAATTATACTGGCGGAGGAAACTTTATTTGCCACAGATGGTTCCG
```

***Agrilus planipennis* (emerald ash borer)**

Utopia-1_APl
The 3' terminus is not sequenced.

***Acyrthosiphon pisum* (pea aphid)**

```
Utopia-1_APi                   TACCCCCCCCCCCCATGTTTTTTGTCCTCCCCATGTCTGTATTCACCATAAACATTAACAAATAAATAAC
U2 snRNA gene (fruit fly)      -------------------------------ATCGCTTCTCGGCCTTATGGCTAAGATCAAAGTGTAGTATCTGTTCTTATCAGCTTAACATCTGATAGTTCCTCCA
Contig44797_[2764-2695]        TACCCCCCCCCCCCATGTTTTTTGTCCTCCCCATGTCTGTATTCACCATAAACATTAACAAATAAATAACTCTGTTCTTATCAGCTTAACATCTGATACACCTTTCA
Contig69178_[1276-1213]        ------CCCCCCCCATGTTTTTTGTCCTCCCCATGTCTGTATTCACCATAAACATTAACAAATAAATAACTCTGTTCTTATCAGCTTAACATCTGATACACCTTTCA
Contig19516_[2174-2111]        ------CCCCCCCCATGTTTTTTGTCCTCCCCATGTCTGTATTCACCATAAACATTAACAAATAAATAACTCTGTTCTTATCAGCTTAACGCCGATTAAACAGATCC
Contig65555_[1404-1341]        ------CCCCCCCCATGTTTTTTGTCCTCCCCATGTCTGTATTCACCATAAACATTAACAAATAAATAACTCTGTTCTTATCAGCTTAACGACAGCGATCCGCTTCC
Contig17834_[1490-1554]        TACCCCCCCCCCCCATGTTTCTTT-CCGCCCCATTTTTGTATTCACCATAAACATTA-CA--TAATAAACTCTGTTCTTATTCGTATCAACTTCTTCGTACAGCTAC
Contig17835_[5637-5573]        TACCCCCCCCCCCCATGTTTCTTT-CCGCCCCATTTAGTATTCACCATAAACATTA-CA--TAATAAACTCTGTTCTTATTCGTATCAACTTCTTCGTACAGCTAC
Contig19151_[13967-14026]      -----CCCCCCCCTATGTTTCTTT-CCGCCCCAATCTTGTACTTACCATAAACATTG-AA--TAATAAACTCTGTTCTTATCAGCTTAACTGCTTTAACTTCAGTCC
Contig17833_[1488-1544]        ---------CCCCCCCTTTTTTATGCACCCCCATACTTGTAT-CACCATAAACATTCCACAAATAAAA-CTCTATCTTAAACCGTGATTTTTATGTATCTATTATCT
```

```
Utopia-2_APi                   TCTCGATTTATTTTGTCTATGTAGGTTCCCCATTTTTGTGAATTTTTTTAAGCATTTATAATTGAATAAA
U2 snRNA gene (fruit fly)      --------------------------------ATCGCTTCTCGGCCTTATGGCTAAGATCAAAGTGTAGTATCTGTTCTTATCAGCTTAACATCTGATAGTTCCTCCA
Contig72453_[896-827]          TCTCGTTTTATTTTGTCTATGTAGGTTCCCCATTTTTGTGAATTTTTTTAAGCATTTATAATTGAATAAA-TCTGTTCTTATCAGCTTAACATCTGATACACCTTTCA
Contig67620_[2417-2348]        TCTCGATTTATTTTGTTTATGTAGGTTCCCCATTTTTGTGAATTTTTTTAAGCATTTATAATCGAATAAA-TCTGTTCTTATCAGCTTAACATCTGATACACCTTTCA
Contig19151_[9438-9507]        TCACGATTTATTTTGTCTATGTAGGTTCCCCATTGTTGTGAATTTTTTTAAGCATTTATACTTTAATAAA-TCTGTTCTTATCAGCTTAACGAGGTTCTTTTTTATAA
Contig47041_[2214-2282]        TCTCTATTTCTTTTTTCTATGTAGGTTCCCCATTGTTGTGA-TTTTTTTAAGCATTTATACTCTAATAAAATCTGTTCTTATCAGCTTAACTGCAAGCGTATCCAACT
Contig15706_[4922-4866]        ----------TTTTATTTATGTAGGTTCCTCATTTTTGTGA--TTTTTTAAACATTGATATCTAATAAAA-TCTGTTCTTATCGGCTTAACGAGAGTAAATCCTACGG
Contig56270_[649-718]          TCTCGATTTATTTTGTCTATGTAGGTTCCCCATTTTTGTGAATTTTTTTAAGCATTTATAATTGAATAAA-TCTGTTCAAATCTGTAACAAATCTTTGAGACTTAAAC
```

***Ladona fulva* (Scarce chaser)**

Utopia-1_LFu
The 3' terminus is uncertain.

Utopia-2_LFu
The 3' terminus is uncertain.

Utopia-3_LFu
The 3' terminus is uncertain.

Utopia-4_LFu
The 3' terminus is uncertain.

Utopia-5_LFu
The 3' terminus is uncertain.

Utopia-6_LFu
The 3' terminus is uncertain.

***Daphnia pulex* (water flea)**

Utopia-1_DPu
The 3' terminus is uncertain.

Utopia-2_DPu
The 3' terminus is uncertain.

```
Utopia-3_DPu                   TGATCACATCCGCCGTAACGAMCGACCTGCCCCCACCGAMTCTACWCCATAATTC-AGCCATC
U2 snRNA gene (fruit fly)      -------------------------ATCGCTTCTCGGCCTTATGGCTAAGATCAAAGTGTAGTATCTGTTCTTATCAGCTTAACATCTGATAGTTCCTCCA
ACJG01010356_[70-1]            TGATCACATCCGCCGTAATGAACGACCTACCCCCACCGAATCTACACCATAATTC-AGCCATCATCTGTCTA
ACJG01010545_[5823-5894]       TGATCACATCCGCCGTAACGACCGACCTGCCCCCACCGACTCTACTCCATAATTCCAGCCATCGTTTTGTCTCTCTCTTTCTGTCATTTACCTTCACCTTC
```

***Strigamia maritima* (centipede)**

```
Utopia-1_SM                    ACAACTAACTTATTTCTGTTTGTTTCATTGTTTCACCTCGTAAGAGGAAGTTCATTGTGCGATAAATCAA
U2 snRNA gene (fruit fly)      -------------------------------ATCGCTTCTCGGCCTTATGGCTAAGATCAAAGTGTAGTATCTGTTCTTATCAGCTTAACATCTGATAGTTCCTCCA
AFFK01010958_[4747-4816]       ACAACTAACTTATTTCTGTTTGTTTCATTGTTTCACCTCGTAAGAGGAAGTTCATTGTGCGATAAATCAATCTGTTCTTATCGGCTTAATCTCCGGTACGTTACCTA
```

***Ixodes scapularis* (deer tick)**

```
Utopia-1_IS                    CTCTAACTTACCAAAATTAAGCAATCACCGATGGAAACCCTGTATCTATGTAATTAGCATCAATAAAAAA
U2 snRNA gene (fruit fly)      -------------------------------ATCGCTTCTCGGCCTTATGGCTAAGATCAAAGTGTAGTATCTGTTCTTATCAGCTTAACATCTGATAGTTCCTCCA
ABJB010432309_[291-360]        CTCTAACTTACCAAAATTAAGCAATCACCGATGGAAACCCTGTATCTATGTAATTAGCATCAATAAAAAATCTGTTCTTATCAGCTTAATATCTGATACGGGTCCTA
ABJB010705441_[919-858]        --------TACCAAAATTAAGCAATCACCGATGGAAACCCTGTATCTATGTAATTAGCATCAATAAAAAATCTGTTCTTATCAGCTTAATATCTGATACGGGTCCTA
```

***Parasteatoda tepidariorum* (common house spider)**

```
Utopia-1_PT                    TTTGCTGTTATTTTTTAGTACCTCGCCTATGATTAGGAAACTCCTATGAGGCACCTCCCCCACCCAAAAA
U2 snRNA gene (fruit fly)      -------------------------------ATCGCTTCTCGGCCTTATGGCTAAGATCAAAGTGTAGTATCTGTTCTTATCAGCTTAACATCTGATAGTTCCTCCA
AOMJ01245954_[17490-17421]     TTTGCTGTTATTTTTTAGTACCTCGCCTATGATTAGGAAACTCCTATGAGGCACCTCCCCCACCCAAAAATCTGTTCTTATCAGCTTAATATCTGATACGTTACTCA
AOMJ01125151_[4664-4595]       TTTGCTGTTATTTTGTAGTACCTCGCCTATGATTAGGAAACTCCTATGAGGCACCTCCCCCACCCAAAAATCTGTTCTTATCAGCTTAATATCTGATACGTTACTCA
AOMJ01160383_[23446-23514]     TTTGCTGTTAATTTTAGTACCTCGCCTATGATTAGGAAACTCCTATGAGGCACCTTCCACACCCAAAAA-TCTGTCATCAGACTTACCAAGAGATGACCAAAGCCAC
AOMJ01109883_[37471-37403]     TTTGCTGTTATTTTTTAGTACCTCGCCTATGATTAGGAAACTCCTATGAGGCACCTCCCCCACCCAAAA-TTTTATTAGGGTAACAAAAATTTTAACCATTGTAAAA
```

***Trichinella spiralis* (nematode)**

```
Utopia-1_TSP                   GTATTT--AATTTTG-CTATTAACAATTCAGTTTGTTAACTGTTTTGTATT-CATTTGAAGATCCAAATAAAAC
U2 snRNA gene (C.elegans)      ----------------------------------ATCGCTTCTTCGGCTTATTAGCTAAGATCAAAGTGTAGTATCTGTTCTTATCGTATTAACCTACGGTATACACTCGA
ABIR02006578_[578-509]         GTATTT--AATTTTG-CTATTAACAATTCAGTTTGTTAACTGTTTTGTATT-CATTTGAAGATCCAAATAAAACTCTGTTCTTATCAGCTTAATTTCTGGTATGAATCCCA
ABIR02006372_[418-349]         GTATTT--AATTTTG-CTATTAACAATTCAGTTTGTTAACTGTTTTGTATT-CATTTGAAGATCCAAATAAAACTCTGTTCTTATCAGCTTAATTTCTGGTATGAATCCCA
ABIR02005094_[44-113]          GTATTT--AATTTTG-CTATTAACAATTCAGTTTGTTAACTGTTTTGTATT-CATTTGAAGATCCAAATAAAACTCTGTTCTTATCAGCTTAATTTCTGGTATGAATCCCA
ABIR02001268_[544-475]         GTATTT--AATTTTG-CTATTAACAATTCAGTTTGTTAACTGTTTTGTATT-CATTTGAAGATCCAAATAAAACTCTGTTCTTATCAGCTTAATTTCTGGTATGAATCCCA
ABIR02001268_[6047-5978]       GTATTT--AATTTTG-CTATTAACAATTCAGTTTGTTAACTGTTTTGTATT-CATTTGAAGATCCAAATAAAACTCTGTTCTTATCAGCTTAATTTCTGGTATGAATCCCA
ABIR02001265_[70068-69999]     GTATTT--AATTTTG-CTATTAACAATTCAGTTTGTTAACTGTTTTGTATT-CATTTGAAGATCCAAATAAAACTCTGTTCTTATCAGCTTAATTTCTGGTATGAATCCCA
ABIR02003119_[80-150]          GTATTT--AATTTTG-CTATTAACAATTCAGTTTGTTAACTGTTTTGTATTTCATTTGAAGATCCAAATAAAACTCTGTTCTTATCAGCTTAATTTCTGGTATGAATCCCA
ABIR02002588_[2031-1961]       GTATTT--AATTTTG-CTATTAACAATTCAGTTTGTTAACTGTTTTGTATTTCCTTTGAAGATCCAAATAAAACTCTGTTCTTATCAGCTTAATTTCTGGTATGAATCCCA
ABIR02006176_[357-288]         GTATTT--AATTTTAATTACTAACAATTCAGTTTTGAAACTGTTTTGTATT-CATTTGAAGATCCAAATAAAAATCTGTTCTTATCAGCTTAATTTCTGGTATGAATCCCA
ABIR02006045_[700-769]         GTATTT--AATTTTAATTACTAACAATTCAGTTCTGAAACTGTTTTGTATT-CATTTGAAGATCCAAATAAAAATCTGTTCTTATCAGCTTAATTTCTGGTATGAATCCCA
ABIR02001265_[61979-61910]     GTATTT--AATTTTAATTACTAACAATTCAGTTCTGAAACTGTTTTGTATT-CATTTGAAGATCCAAATAAAAATCTGTTCTTATCAGCTTAATTTCTGGTATGAATCCCA
ABIR02001305_[1566-1614]       ----------------------ACAACTCAGT--ACTAACTGTTTTGTATT-CATTTGAAGATCCAAATAAAACTCTGTTCTTATCAGCTTAATTTCTGGTATGAATCCCA
ABIR02006045_[1437-1507]       GTATTTTAAATTTTGTTTCCTTACAATTCAGTTTGTTAATTGTTTTGTATT-G-TTTGACGATCCAAATAAAATTTCTTTTTTTTACAGCTTAATTTCTGGTATGAATCTC
ABIR02001265_[69338-69268]     GTATTTTAAATTTTGTTTCCTTACAATTCAGTTTGTTAATTGTTTTGTATT-G-TTTGACGATCCAAATAAAATTTCTTTTTTTAACAGCTTAATTTCTGGTATGAATCCC
```

***Pristionchus pocificus* (nematode)**

```
Utopia-1_PPac                    TACTCACTATTCACGTATGTTTTCGTGTACTGTACCGCTCTGCGGAATTAACCTTGTATGATAAATCTTC
U2 snRNA gene (C.elegans)        ------------------------------ATCGCTTCTTCGGCTTATTAGCTAAGATCAAAGTGTAGTATCTGTTCTTATCGTATTAACCTACGGTATACACTCGA
chrUn_[100460264-100460333]      TACTCACTATTCACGTATGTTTTCGTGTACTGTACCGCTCTGCGGAATTAACCTTGTATGATAAATCTTCTCTGTTCTTATCGGCTTAATCTCCGGTACGATGACTA
chrUn_[114822581-114822516]      TACTCACTATTCAC---TGT-ATCGTGTACTGTACCGCTCTGCGGAATTAACCTTGTATGATAAATCTTCTCTGTTCTTATCGGCTTAATCTCCGGTACGTTGTCTA
```

***Ciona savignyi* (sea squirt)**

```
YURE-2_Cis                             TCATGATA-ATATTTCGCTCCTTTTTTGCCCCGTGTAAACTTAGTNGATGCGAATAAAA-TCAGTTGAATCA
U2 snRNA gene (human)                  ----------ATCGCTTCTCGGC-CTTTTG-GCTAAGATCAAGTGTAGTATCTGTTCTTATCAGTTTAAT-ATCTG-----------ATACGT--CCTCTA
U2_sat (Ciona savignyi)                ----------ATCGCTTCTCGGCTCTTTTGAGCTAAGATCAAGTGTAGTATCTGTTCTTCTCAGTTTAAT-ACCTGGGACGGAAACGATTCGTTTCCTCTA
paired_scaffold_386_[228455-228524]    TCATGATA-ATATTTCGCTCCTTTTTTGCCCCGTGTAAACTTAGTAGATGCGAATAAAA-TCAGTTGAATCACCTGGGACGGAAACGATTCGT--CCTCTA
paired_scaffold_248_[246339-246271]    TTATGATA-ATATTTCGCTCTTATTTTGCCTTGTGTAA-CTTAGTTGATGCAAATAAAA-TCAGTTGAATCACCTGGGACGGAAACGATTCGTTTCCTCTA
paired_scaffold_120_[144218-144286]    TTATGATA-ATATTTCGCTTTTATTTTGCCTTGTGTAA-CTTAGTTGATGCAAATAAAA-TCAGTTGAATCACCTGGGACGGAAACGATTCGTTTCCTCTA
paired_scaffold_377_[1038767-1038700]  --ATGATA-ATATTTCGCTCCTTTTTTGCCCCGTGTAAACTTAGTAGATGCGAATAAAA-TCAGTTGAATCATATTATTAAATCAGCATACTTGATATTTA
paired_scaffold_379_[39720-39788]      TTATGATA-ATATTTCGCTCCTTTTTTGCCCCGTGTAAACTAAGTAGATGCGAATAAAA-TCAGTTGAATCTGTCAGATAAGCATAATTATCATACACGAC
paired_scaffold_322_[428849-428917]    TTATGATA-ATATTTCGCTCCTATTTTGCCTTGTGTAA-CTTAGTTGATGCAAATAAAA-TCAGTTGAATCATCTTACGCTTGGACGAAGTTTCTGCTTAT
paired_scaffold_146_[63915-63983]      TTATGATA-ATATTTCGCTCCTATTTTGCCTTGTGTAA-CTTAGTCGACGCAAATAAAA-TCAGTTGAATCAAATCTTATATGAAATAATCTTAATTTTCC
paired_scaffold_66_[403049-402981]     TTATGATA-ATATTTCGCTCCTATTTTGCCTTGTGTAA-CTTAGTTGATGCAAGTAAAA-TCAGTTAAATCAAAATTTCTGTCTAGATTCACCATCACACA
paired_scaffold_152_[22143-22211]      TTATGATA-ATATTTCGCTCCTTTTTCGCTTTGTGTGA-CTTAGTTGACGCAAATAAAA-TCAGTTGAATCAAATCAGGGCGAGGATGTACACGTCATCGG
paired_scaffold_157_[865050-865119]    TTATGATATATATTTCGCTCCTATTTTGCTTTGTGTGA-CTTAGTTGATGCAAATAAAA-TCAGTTGAATCAACAAATAAACATTATAACAAAAGGACAAA
paired_scaffold_323_[357708-357774]    TTATGATA-ATATTTCGCTCCTTTTTCGTCCCGTACAAA-TT-GT-GACGCAAATAAAA-TCAGTTGAATCAAGTGGGTGCACCAAATAAAATCGAATGCT
paired_scaffold_422_[1375897-1375829]  TTATGATA-ATATTTCGCTCCTATTTTGACTTGTGTTA-CATAGTGGATGCAAATAAAA-TCAGTTGAATCAAAATATTTTTCTATTTTATTTTTTTGCGC
paired_scaffold_377_[658467-658403]    TCATGATA--TATTTCGCTCCTTTTTCGCCTTGTGAA---TTAGT-GATGCAAATAAAA-TCAGTTGAATCATATGCATTCGTCTGACTCGCTTACGTACT
paired_scaffold_323_[699049-699114]    TTATGATA-ATATTTCGCTCCTTTTTCGCCCCGTGCAAA-TT-GT--GCACAAATAAAA-TCGGTTGAATCAAAAAACAGTTTTTCCTCCTTCCCTTTCAA
paired_scaffold_248_[271418-271352]    TCATGATA-ATATTTCGCTCCTTTTTACACT-GTAAATAA---GTTGATGCGAATAAAAATCAGTTGAATCAATCTCAGTTGCATATAAAATTTTAAATAT
paired_scaffold_112_[1168712-1168643]  TTATGATATATATTTCGCTCCTATTTCGCTTTGTGTGA-CTTAGTTGACGCAAATAAAA-TCAGTTAAATCAAAACAGTCATTGACCATTGTCATTCCATC
paired_scaffold_58_[59253-59189]       --ATGATA-ATATTTCTCTCCTTTTTCGCCCTGTACAAA-TT-GT-GACGCAAATAAAA-TCAGTTGAATCATCTTTCAACTAGATATACACTGCAGTGAA
paired_scaffold_142_[416455-416390]    TTATGATA-ATATTTCGCTCCTTTTTCGCACTGTGATTAAGT----GATGCAAATAAAA-TCAGTGGAATCAAAAAATGGATCAAATCGCCAAGTCCTTCT
paired_scaffold_445_[434008-433945]    TTATGATA-ATATTTCGCTCCTTTTTCGCACTGTGAATAAGT----GATGCAAATAAAA-TCAGTTGAATAAATTCGCGTACATGCAGAACAAGAATTACA
paired_scaffold_27_[589545-589607]     TTATGATA--TATTTCGCTCCTATTTCGCCCTGTGAATTAGT----GATGCAAATAAAA-TCAGTTGAATGACAGGGGTGACTAAGTAGCCACATTAGACC
```

```
YURE_CSa                               TAACAATATGCGCGCAACGCAAAAACGAAAAAGCTCACGAAAAAGCTCACGCAAAAGCGCAACCACAAAA
U2 snRNA gene (human)                  ----------ATCGCTTCTCGGC-CTTTTG-GCTAAGATCAAGTGTAGTATCTGTTCTTATCAGTTTAAT-ATCTG-----------ATACGT--CCTCTA
U2_sat (Ciona savignyi)                ----------ATCGCTTCTCGGCTCTTTTGAGCTAAGATCAAGTGTAGTATCTGTTCTTCTCAGTTTAAT-ACCTGGGACGGAAACGATTCGTTTCCTCTA
paired_scaffold_299_[92458-92527]      TAACAATATGCGCGCAACGCAAAAACGAAAAAGCTCACGAAAAAGCTCACGCAAAAGCGCAACCACAAAAGCGCAAACACAAACGCGCCACACTTTATCCA
paired_scaffold_299_[85847-85916]      TAACAATATGCGCGCAACGCAAACACGAAAAAGCTCACGAAAAAGCTCACGCAAAAGCGCAACCACAAAAGCGCAAACACAAACGCGCCACACTTTATCCA
paired_scaffold_287_[37293-37349]      TAACAATATGCGCGCAACGCAAACACGAAAAAGCTCACGCAAAAGCGCAACCACAAACGCG-CCACACTTTATCCATCACTAGACCCTCCCAAAGCATGAT
paired_scaffold_283_[456511-456442]    TAACAATACGCGCGCAAAAGCAAAGAAAAAA-GCCCACGCAAAAGCGCAACTACAAAAGCGCAACTACAAATACTTGTCACAAAAGCGCAAGCACGAATGT
paired_scaffold_337_[329266-329322]    TAACAATATGCGCACAAAATCACACAAAAAAGCACACGCAAAAGCGCAACCACAAAAGCGCAAGCACAAATGCGTCACAATTAATCCACCACTAGACCCTC
```

***Ciona intestinalis* (sea squirt)**

```
YURECi                        CAATTTTATTCATTGCATTTTATCCGCGTCGT--GGTGTTTGCGTGGATGCATTAATAAAAGATGAAATT-CC
U2 snRNA gene (human)         --------ATCGCTTCTCGGC-CTTTTG-GCTAAGATCAAGTGTAGTATCTGTTCTTATCAGTTTAA-TA-TCTG-----------ATACGT--CCTCTA
U2_sat (Ciona savignyi)       --------ATCGCTTCTCGGCTCTTTTGAGCTAAGATCAAGTGTAGTATCTGTTCTTCTCAGTTTAA-TA-CCTGGGACGGAAACGATTCGTTTCCTCTA
scaffold_301_[25086-25023]    -AATTTTATTCATTGCATTTTATCCGCGTCGT--GGTGTTTGCGTGGATGCATTAATAAAAGATGAA-TC-CCTGGGACGGAAACGATTCGTTTCCTCTA
scaffold_209_[58401-58465]    CAATTTTATTTATTGCATTTTATCCGCGTCGT--GGTGTTTGCGTGGATGCATTAATAAAAGATGAA-CC-CCTGGGACGGAAACGATTCGTTTCCTCTA
scaffold_120_[223750-223819]  CAATTTTATTCATTGCATTTTATCCGCGTCGT--GGTGTTTGCGTGGATGCAGTAATAAATGATGAAATT-CCTGGTCTTTTCCCCATCCCCTTTTGTCT
scaffold_879_[16798-16867]    CAATTTTATTCATTGCATTTTATCCGCGTCGT--GGTGTTTGCGTGGATGCAGTAATAAATGATGAAATT-CCTGGTCTTTTCCCCATCCCCTTCTGTCT
chr05q_[1182214-1182283]      CAATTTTATTCATTGCATTTTATCCACGTCGT--GGTGTTTGCGTGGATGCAGTAATAAATGATGAAATT-CCTGGTCTTTTTCCCATTCCCTTCTGTCT
chr05q_[1182632-1182701]      CAATTTTATTCATTGCATTTTATCCACGTCGT--GGTGTTTGCGTGGATGCAGTAATAAATGATGAAATT-CCTGGTCTTTTTCCCATTCCCTTCTGTCT
chr07q_[3145403-3145354]      -------------------TTATCCGCGTCGT--GGTGTTTGCGAGGATGCATGAATAAAAGATGAA-TT-TCTGTGCCCCAGCAGATACAGGTGATCGA
chr06q_[2451728-2451660]      CAATTTTATTTATTGCATTTTATCCGCGTCGT--GGTGTTTGCGTGGATGCATTAATAAAAGATGAA-TT-CCAATACTATAGGCGCCAAATCCATAAAT
scaffold_120_[22425-22357]    CAATTTTATTTATTGCATTTTATCCGCGTCGT--GGTGTTTGCGTGGATGCATTAATAAAAGATGAA-TT-CCAATACTATAGGCGCCAAATCTGCGGTG
scaffold_362_[21068-21136]    CAATTTTATTTATTGCATTTTATCCGCGTCGT--GGTGTTTGCGTGGATGCATTAATAAAAGATGAA-TT-CCAATACTATAGGCGCCAAATCCATAAAT
scaffold_120_[92582-92650]    CAATTTTATTTATTGCATTTTATCCGCGTCGT--GGTGTTTGCGTGGATGCATTAATAAAGGATGAA-TT-CCAATACTATAGGCGCCAAATCCATAAAT
scaffold_362_[60311-60379]    CAATTTTTTTTATTGCATTTTATCCGCGTCGT--GGTGTTTGCGTGGATGCATTAATAAAAGATGAA-TT-CCAATACTATAGGCGCCAAATCTGCAGTG
scaffold_641_[1004-1072]      CAATTTTTTTTATTGCATTTTATCCGCGTCGT--GGTGTTTGCGTGGATGCATTAATAAAAGATGAA-TT-CCAATACTATAGGCGCCAAATCTGCGGTG
scaffold_120_[51578-51509]    CAATTTTATTTATTGCATTTTATCCTGCGTGG--TGGTGTTTGCGTGGATGCATTAATAAAAGATGAATT-CCAATACTATAGGCGCCAAATCCATAAAT
scaffold_120_[94202-94258]    ------------TTGCATTTTATCC-GCGTCG--TGGTGTTTGCGTGGATGCATTAATAAAAGATGAATT-CCAATACTATAGGCGCCAAATCTGCGGTG
chr05q_[107660-107724]        CAATTTTATTTATTGCTTTTTATCCGCGTCGT--GGTGTTTGCGTGGATGCATTAATAAAAGATGAA-CC-CCACTAACGTGTTCAAAAATGTGGCCGTT
scaffold_113_[172169-172233]  CAATTTTATTTATTGCTTTTTATCCGCGTCGT--GGTGTTTGCGTGGATGCATTAATAAAAGATGAA-CC-CCACTAACGTGTTCAAAAATGTGGCCGTT
scaffold_209_[39131-39194]    -AATTTTATTTATTGCATTTTATCCGCGTCGT--GGTGTTTGCGTGGATGCATTAATAAAAGATGAA-CC-CCCCTGGGACGGAAACGATTCGTTTCCTC
scaffold_427_[42796-42725]    CAATTTTATTTATTGCATTTTATCCGCGTCGTGTGGTGTTTGCGTTGATGCATTAATAAAAGATAAAATTTCCGCCTGCAACTTATATTACAAAGCACCG
scaffold_62_[493188-493250]   CAATTTAATTTATTGCATTTTATCCGCGTCGT--GGTGTTTGCGTGAAAGCATAAATAAAATATGTAAAA-CCTCGTCTTGACAAAAAAAGTTATATTAT
```

***Chrysemys picta bellii* (painted turtle)**

```
Utopia-1_CPB                  GAAACTTCTATGCTCAAACTCTGTTCACTATTTTTTTT-AACATCATCTTAATAAAATTTTTAAA
U2 snRNA gene (human)         ---------------------------ATCGCTTCTCGGCCTTTTGGCTAAGATCAAGTGTAGTATCTGTTCTTATCAGTTTAATATCTGATACGTCCTCTA
AHGY01527796_[3021-3090]      GAAACTTCTATGCTCAAACTCTGTTCACTATTTTTTTT-AACATCATCTTAATAAAATTTTTAAATCTGTTCTTCTCAGTTTAATATCTGATATGTTCTTTA
AHGY01044076_[19116-19185]    GAAACTTCTATGCTCAAACTCTGTTCACTATTTTTTTT-AACATCATCTTAATAAAATTTTTAAATCTGTTCTTATCCATGACACACATTGTAAGAGTGGGG
AHGY01060163_[12745-12814]    GAAACTTCTATGCTCAAACTCTGTTCACTATTTTTTTT-AACATCATCTTAATAAAATTTTTAAATCTGTTCTTTCTTTCCCCTTTCTGTACCTCAGTTTAT
AHGY01062633_[4030-3961]      GAAACTTCTATGCTCAAACTCTGTTCACTATTTTTTTT-AACATCATCTTAATAAAATTTTTAAATCTGTTTTCATTTTGGGTATGTGTGAGCCGCTGAAAA
AHGY01131761_[23553-23484]    GAAACTTCTATGCTCAAACTCTGTTCACTATTTTTTTT-AACATCATCTTAATAAAATTTTTAAATCTGTTTTTAGCTATTCATTTGAAAGTTTCTCATGGC
AHGY01144444_[19541-19610]    GAAACTTCTATGCTCAAACTCTGTTCACTATTTTTTTT-AACATCATCTTAATAAAATTTTTAAATCTGTTAAAAAAGCTTCCCCACCCTAAAAGAAAAATA
AHGY01158080_[1312-1381]      GAAACTTCTATGCTCAAACTCTGTTCACTATTTTTTTT-AACATCATCTTAATAAAATTTTTAAATCTGTTCTTATCAGAGGGGTAGCCATGTTAGTCTGGA
AHGY01108749_[1566-1498]      GAAACTTCTATGCTCAAACTCTGTTCACTATTTTTTTT-AACATCATCTTAATAAAATTTTTAAATCTGTGCACTATAGCATTTGCACATCTGAGTCCTTAG
AHGY01237353_[9894-9825]      GAAACTTCTATGCTCAAACTCTGTTCACTATTTTTTTT-AACATCATCTTAATAAAATTTTTAAACCTGTTATCGGCTAGAACTTAAGTGGAGCCATTCCTG
AHGY01074155_[34645-34578]    GAAACTTCTATGCTCAAACTCTGTTCACTATTTTTTTT-AACATCATCTTAATAAAATTTTTAAATCTGCACAGATTCAGGGTGGCATTGGGGACGATAGAG
AHGY01317497_[5303-5236]      GAAACTTCTATGCTCAAACTCTGTTCACTATTTTTTTT-AACATCATCTTAATAAAATTTTTAAATCTGAAAAAAAAGAGGCAGGAGATTGCAGGAAAAGAA
AHGY01336528_[3798-3731]      GAAACTTCTATGCTCAAACTCTGTTCACTATTTTTTTT-AACATCATCTTAATAAAATTTTTAAATCTGAGCACCTTACCACACCCCTGTGAGGTAGGAAAG
AHGY01155193_[2124-2193]      GAAACTTCTATGCTCAAACTCTGTTCACTATTTTTTTT-AACATCATCTTAATAAAATTTTTAAATCGGTTACTGCTGCCTGCCACAGGGACACATCAACTG
AHGY01403853_[1628-1696]      GAAATTTCTATGCTCAAACTCTGTTCACTATTTTTTTT-AACATCATCTTAATAAAATTTTTAAATCTGTACTGTCAAGTTCCCATAAAGAGAGAAACAAAT
AHGY01520692_[1232-1164]      GAAACTTCTATGCTCAAACTCTGTTCACTATTTTTTTT-AACATCATCTTAATAAAATTTTTAAATCTATGCTGGTGATCACTTGACTGATCACTTTCTTGA
AHGY01232364_[3456-3387]      GAAACTTCTATGCTCAAACTCTGTTCACTTTTTTTTTT-AACATCATCTTAATAAAATTTTTAAATCTGTTAATAGAGTGATAGGGCATCTAGAGCCTTGTG
AHGY01508815_[5738-5669]      GAAACTTCTATGCTCAAACTCTGTTCACTATTTTTTTT-ATCATCATCTTAATAAAATTTTTAAATCTGTTCTTTTCAGCCCTAAGCAATGATCACTCAGAC
AHGY01088444_[3550-3616]      GAAACTTCTATGCTCAAACTCTGTTCACTATTTTTTTT-AACATCATCTTAATAAAATTTTTAAATCTCTCTGCCAGTCTCCCCATAGCCTAAGGGGTAAGG
AHGY01010951_[12726-12796]    GAAACTTCTATGCTCAAACTCTGTTCACTATTTTTTTTTAACATCATCTTAATAAAATTTTTAAATCTGTTCTTATCTCTAAGTAGTTTTTTGATGTTCCTC
AHGY01360647_[5072-5142]      GAAACTTCTATGCTCAAACTCTGTTCACTATTTTTTTTTAACATCATCTTAATAAAATTTTTAAATCTGTTTTTAGTCCATTTCTTGTATTTCTGGTTTCCT
```

```
Utopia-1B_CPB                 ATGTACAGTACCTTCCCTCTTAACTCGTGTATATTTAATTTTAAACATTAACTTTAATAAAATTTTTAAA
U2 snRNA gene (human)         ---------------------------------ATCGCTTCTCGGCCTTTTGGCTAAGATCAAGTGTAGTATCTGTTCTTATCAGTTTAATATCTGATACGTCCTCTA
AHGY01117997_[4626-4557]      ATGTACAGTACCTTCCCTCTTAACTTGTGTATATTTAATTTTAGACATTAGTTTTAATAACATTTTAAAA-TCTATTCTTATCATTTGAATATCTAATATGTCCTTTA
AHGY01290635_[48327-48258]    ATGTACATTACCTTCCCTCTTAACTTGTGTATATTTAATTTTAAACATTAACTTTAATAACATTTTTAAA-CCTGTTCTTATCTGTAAGATATTTGAATAGGTTATCT
AHGY01474619_[283-350]        --GCACAGTACCTTCCCTCTTAACTCGTGGATACTTAATTTTAAACATTAACTTTAATAAAAATTGTAAAACCTGTTCTTATCAGTTTCATATCTGATATGTCCTTTA
AHGY01251864_[235-168]        --GCACAGTACCTTCCCTCTTAAGTCGTGTCTATTTAATTTTAAACATTAACTTTAATAAAAATTGTAAAACCTGTTCTTATCAGTTTCATATCTGATATGTCCTTTA
AHGY01406714_[235-168]        --GCACAGTACCTTCCCTCTTAAGTCGTGAATATTTAATTTTAAACATTAACTTTAATAAAAATTGTAAAACCTGTTCTTATCAGTTTCATATCTGATATGTCCTTTA
AHGY01339126_[4170-4101]      ATGTACAGTACCTTCCCTCTTAACTTGTGTATACTTAATTTTAAACATTAACTTTAATAAAATTTTTAAA-TCTGTTCTCTGTGGGAAACATTGCTCATCTGGTGCTA
AHGY01516814_[115-46]         ATGTACAGTACCTTCCCTCTTAACTTGTGTATATTTAATTTTAAACATTAACTTGAATAAAATTTTTAAA-TCTGTTGTTGGAGCAACTTCTATTAGTGGAAAGGACA
AHGY01012499_[22331-22262]    ATGTACAGTACCTTCCCTCTTAACTTGTGTATATTTAATTTTAAACATTAACTTTAATAAAATTTTTAAA-TCTGAGTGCTCTCAACTCCCACTGAAGCCAGCTTGAT
AHGY01373345_[22297-22228]    ATGTACAGTACCTTCCCTCTTAACTTGTGTATATTTAATTTTAAACATTAACTTTAATAAAATTTTTAAA-TCTGTCTCCTTCAGAGGCAGTAGCTAGGTTGACATAA
AHGY01148758_[2523-2592]      ATGTACAGTACCTTCCCTCTTAACTTGTGTATATTTAATTTTAAACATTAACTTTAATAAAATTTTTAAA-TCTGTCTAACATGTGAGGATTCAAGTCAGTAGTGACA
AHGY01249424_[18957-19026]    ATGTACAGTACCTTCCCTCTTAACTCGTGTATATTTCATTTTAAACATTAACTTTAATAAAATTTTTAAA-TCTGAGCCATCTTGTACAGTATCTGAGTATCCTGGCT
AHGY01183067_[701-770]        ATGTACAGTACCTTCCCTCTTAACTTGTGTATATTTAATTTTAAACATTAACTTTAATAAAATTTTTAAA-TCTATTCT
AHGY01280305_[13562-13631]    ATGTACAGTACCTTCCCTCTTAACTTGTGTATATTTAATTTTAAACATTAACTTTAATAAAATTTTTAAA-TTTGTTAACTGTGCTTCCTGCAAAAGTACAACGTGTC
AHGY01287058_[89-20]          ATGTACAGTACCTTCCCTCTTAACTTGTGTATATTTAATTTTAAACATTAACTTTAATAAAATTTTTAAA-CTTGCTGCCTCACTGCTGC
AHGY01019227_[4383-4314]      ATGTACAGTACCTTCCCTCTTAACTCGTGTATATTTAATTTTAAACATTCACTTTAATAAAATTTTTAAA-TTTCTGAAAGGACTCTTTTCAACTATAAAGCTCACCA
AHGY01018078_[4076-4007]      ATGTACAGTACCTTCCCTCTTAACTTGTGTATATTTAATTTTAAACATTAACTTTAATAGAATTTTTAAA-TACATCAGGGATTGCAGGAAGAGAAAGGCTGCTCTCA
AHGY01095141_[6181-6112]      ATGTACAGTACCTTCTCTCTTAACTTGTGTATATTTAATTTTAAACATTAACTTTAATAAAATTTTTAAA-TCTTCTTGGAGCATTTAAGATCAGATGAGACTTCACT
AHGY01195534_[89-22]          ATGTACAGTACCTTCCCTCTTAACTTGTGTATATTTAATTTTAAACATTAACTTTAATAAAATTTTTA---GTGCTTTTCATCAGGAAATCT
AHGY01291750_[1148-1081]      ATGTACAGTACCTTCCCTCTTAACTTGTGTATATTTAATTTTAAACATTAACTTTAATAAAATTTTTATA-TCCATCCTTTTTCCCATATCTAACATCTGTGACCTAT
AHGY01299186_[14271-14339]    ATGTACAGCACCTTCCCTCTTAACTTGTGTATATTTAATTTTAAACATTAACTTTAATAAAATTTTTAA--CTGGACCAACTTCTGTTGGTGGCAGAGACAAGCTTTC
AHGY01322572_[14559-14490]    ATGTACAGTACCTTCCCTCTTAACTTGTGTATATTTAATTTTAAACATTAACTTTAATAAAATGTTTAAA-TCTCTAGATAAGCAGGGAGTAGTGATGCCAAATCCAT
AHGY01387753_[334-265]        ATGTACAGTACCTTCCCTCTTAACTTGTGTATATTTAATTTTAAACATTCACTTTAATAAAATTTTTAAA-TCTTTTTCTTGTTTACATAGGAAGGTGGAAACAGCAA
AHGY01317085_[4395-4329]      ATGTACAGTACCTTCCCTCTTAACTTGTGTATATTTAATTTTAAACATTAACTTTAATAAAATTTTTCCA-TAACCACAAACTCCCATTTTCTTTGAAGCATTTCTCA
AHGY01271176_[11487-11418]    ATGTACAGTACCTTCCCTCTTAACTTGTGTATATTTAATTTTAAACATTAACTTTAATAAAAATTTTAAA-TGTGCATACATACACCACCAGAGTGTGCTCATTGCTT
AHGY01284478_[4788-4719]      ATGTACAGTACCTTCCCTCTTAACTTGTGTATATTTAATTTTAAACATTAACTTTAATAAAAATTTTAAA-TCTATTTCTATTAACATCACCCTACTTGTTCCTGAGA
```

```
Utopia-2_CPB                  GACGCTTCAAATTTTCTGTATCGTTTATTTTTTTT--AAATATTCTCTAATAAACATTTAAA
U2 snRNA gene (human)         ------------------------ATCGCTTCTCGGCCTTTTGGCTAAGATCAAGTGTAGTATCTGTTCTTATCAGTTTAATATCTGATACGTCCTCTA
AHGY01009093_[12238-12305]    -ACGCTTCAAATTTTCTGTATCGTTTATTTTTTCT--AAATATTCTCTAATAAA-ATTTAAATCTGTTCTTATCAGTTTAATATCTGATATGTTCTCGC
```

```
AHGY01507166_[419-352]          -ACGCTTCAAATTTTCTGTATCGTTTATTTCTTTT--AAATATTCTCTAATAAA-ATTTAAATCTGTTCTTATCAGTTTAATATCTGATATGTTCTCGA
AHGY01521395_[569-501]          -ACGCTTCAAATTTTCTGTATCGTTTATTTTTTTTT-AAATATTCTCTAATAAA-ATTTAAATCTGTTCTTATCAGTTTAATATCTGATATGTTCTCGA
AHGY01404873_[1601-1534]        -ACGCTTCAAATTTCCTGTATCGTTTATTTCTTTT--AAATATTCTCTAATAAA-ATTTAAATCTGTTCTTATCAGGATACAGAAGCTATCTCCTGAGT
AHGY01342611_[71-4]             -ACGCTTCAAATTTTCTGTATCGTTTATTTTTTTCT--AAATATTCTCTAATAAA-ATTTAAGTCTGTTCTTATCA
AHGY01065581_[1282-1347]        -ACGCTTCAAATTTTCTGTATCGTTTATTTCTTTT--AAATATTCTCTAATAAA-ATTTAAATCTGTTCTCCACAGCAAGAAGAGTGTTATTCCCTACT
AHGY01080586_[113-48]           -ACGCTTCAAATTTTCTGTATCGTTTATTTTTTTTT--AAATATTCTCTAATAAA-ATTTAAATCTGCTCTGCCCAGGTATGCGAGACCTAAAGTGGCCA
AHGY01315532_[3505-3440]        -ACGCTTCAAATTTTCTGTATCGTTTATTTCTTTT--AAATATTCTCTAATAAA-ATTTAAATCTGTTCTCCCACTACAAGAAAGGGACCACGAGACCT
AHGY01163289_[502-434]          -ACGCTTCAAATTTTCTGTATCGTTTATTTTTTTTTTTAAATATTCTCTAATAAA-ATTTAAATCTGTTCTTCTTATCTGTTCTTAACAGTTTAACGTCT
AHGY01220023_[3475-3542]        -ACGCTTCAAATTTTCTGTATCGTTTATTTCTTTT--AAATATTCTCTAATAAA-ATTTAAATCTGTTTTCATTCTAGCTGTCACCTGGATTTCTCCCT
AHGY01363654_[211-276]          -ACGCTTCAAATTTTCTGTATCGTTTATTTCTTTT--AAATATTCTCTAATAAA-ATTTAAATCTGTCCTCTGACCCCCTCCGCCTCTCTGGCTGGCTG
AHGY01457656_[637-703]          -ACGCTTCAAATTTTCTGTATCGTTTATTTCTTTT--AAATATTCTCTAATAAA-ATTTAAATCTGTTATTGGACCAACAAGACCAAAGCTTGTCTCTT
AHGY01444818_[961-898]          -ACGCTTCAAATTTTCTGTATCGTTTATTTTTTTCT--AAATATTCTCTAATAAA-ATTTAAATCTGTTTATGACATATGCTATTACCGATGTAAGCAGA
AHGY01108263_[1776-1711]        GACGCTTCAAATTTTCTGTATCGTTTATTTTTT----AAATATTCTCTAATAAA-ATTTAAATCTGCTCTTCCAAACTGGGCATCCAAGGAGCAGCATG
AHGY01530517_[1451-1514]        -ACGCTTCAAATTTTCTGTATCGTTTATTTTTTT---AAATATTCTCTAATAAA-ATTTAAATCTGTTCCCCCGCATCGCCTTCGTCCTGAGGGGATCC
AHGY01018027_[2692-2759]        -ACGCTTCAAATTTTCTGTATCGTTTATTTTTTCT--AAATATTCTCTAATAAA-ATTTAAATCTGATATTAGCATGTAAGCTTCTTGGGACAGGGATT
AHGY01307176_[696-635]          -ACGCTTCAAATTTTCTGTATCGTTTATTTTTTCT--AAATATTCTCTAATAAA-ATTTAAATCTGGATGACTTCAGCAGCCAGGTTTAGGTACAGAAA
AHGY01315098_[672-611]          -ACGCTTCAAATTTTCTGTATCGTTTATTTCTTTT--AAATATTCTCTAATAAA-ATTTAAATCTGATGGTATGGGTCCTCTTTGAGGGTTTGAAACGC
AHGY01253071_[660-596]          -ACGCTTCAAATTTTCTGTATCGTTTATTTTTTT---AAATATTCTCTAATAAA-ATTTAAATCTGTTTGGTGGAATTGTTTGAGTGGCAAATGCTGA
AHGY01478533_[613-548]          -ACGCTTCAAATTTTCTGTATCGTTTATTTCTTTT--AAATATTCTCTAATAAA-ATTTAAATCTCCTCTCTCCACTGTTGAATGTCAGAGCTAACTTT
```

```
Utopia-3_CPB                    CACTCTTTTCTCAACCTATGTATTATATAATTTTTTTAACATTAGCTTTAATAAAA-TTTTTAAA
U2 snRNA gene (human)           ---------------------------ATCGCTTCTCGGCCTTTTGGCTAAGATCAAGTGTAGTATCTGTTCTTATCAGTTTAATATCTGATACGTCCTCTA
AHGY01097740_[1790-1857]        CACTCTTTTCTCAACCTATGTATGATATA-TTTTTT-AACATTAGCTTTAATAAAA-TTTTTAAATCTGTTCTTATCAGTTTAATTACTGAGGCAGAAACTA
AHGY01209431_[8385-8316]        CACTCTTTTCTCAACCTATGTATTATATAATTTTTTTAACATTAGCTTTAATAAAAATTTT-AAATCTGTTCCACATGAGTTGGGGTGTCAGAATGTGGTCC
AHGY01199401_[14668-14599]      CACTCTTTTCCCAACCTATGTATTATATAATTTTTTTAACATTAGCCTTAATAAAA-TTTTTAAATCTATTAGCTTCCAGCTGGACCGGTATCAAACCCTTC
AHGY01033298_[6509-6577]        CACTCTTTTCTCAATCTATGTATTATATAATTTTTTTAACATTAGCTTTAATAAAA-TTTTTAAATCGGTCCTTCCATCACCCCTGCTGTGCCCATTCCACA
AHGY01176311_[56934-56865]      CACTGTTTTCTCAACCTATGTATTATATAATTTTTTTAACATTAGCTTTAATCAAA-TTTTTAAATCTGTTTTTATCTCTCATAAAAATGCAAGCAAAGAGC
AHGY01186472_[2894-2825]        CACTCTTTTCCCAACCTATGTGTTATATAATTTTTTTAACATTAGCTTTAATAAAA-TTGTTAAATCTGTTCTTACAGTCCTTTGTATTTCGTTATCCTTCT
AHGY01085841_[750-682]          CACTCTTTTCTCAACCTATGTATTTTATAATTTTTTTAACATTAGCTTTAATAAA-TTTTTTAAATCTGTACCTGTAGTTGAGGGAGCACATCCACCATTTC
AHGY01421757_[1405-1337]        CACTCTTTTCCCAACCTATGTATTATATAATTTTTTTAACATTAGCTTTAATAAAAAATTTTTAAATCTGACTGTGGTCCTGAGACAGCATGTCTGGTCAAAA
AHGY01120423_[5011-4943]        CACTCTTTTCTCAACCGATGTATTATATAATTCTTTTAACATTAGCTTTAATAGAA-TTTTTAAATCTGTAAGAAGGCCTGGAGACAGCTCCTCCTCCTGCT
AHGY01122334_[9372-9441]        CACTCTTTTCCCAACCTATGTATTATATAATTTTTTTAACATGAGCTTTAATAAAA-TGTTTAAATCTGTTTATTTTATCCTACTGCAGGTCTTAGTGAAAC
AHGY01447760_[7233-7166]        CACTCTTTTCTCAACCTATGTATTATATACTTTTTTTAACATTAGCTTTAATAAAAATTTT-AAATCTGAGAATAGGAATCTACACGGATATAACTACACTG
AHGY01029926_[25795-25726]      CACTCTTTTCCCAACCTATGTATTATATAATTTTTTTAACATTAGCTTTAATAAAAAATTT-AAATCTGTTCTACACAGCAATGTTTGCACTGCTAGCATAG
AHGY01047428_[41379-41448]      CACTCTTTTCTCAACCTATGTATTATATAATATTTTAAACATTAGCTTTAATAAAA-CTTTTAAATCTGTTAAATTTCTATTTTTGCAGTGCTTGCAACACC
AHGY01176403_[13086-13017]      CACTCTTTTCTCAACCTATGTATTACATAATTTTTTTAACATTAGCTTTAATTAAAATTTT-AAATCTGTTGCCCTCTACTGATCAGAATGTCACACCTTCT
AHGY01313837_[10742-10673]      CACTCTTTTCTCAACCTATGTATTACATAATTTTTTTAACATTAGCTTTAATAAAA-TTTTAAAATATGTTAAGCTTCTACCCTTTCTGCTCAGAATTAGAG
AHGY01108257_[5219-5288]        CACTCTTTTCTCGACCGATGTATTACATAATTTTTTTAACATTAGCTTTAATAAAG-TTTTTAAATCTGTTCTCTGACATGCCAGAACAATGCGTTCTTGGG
AHGY01477501_[2416-2481]        CACTCTTTTCTCAACCTATGTATTATATAATTTT---AACATTAGCTTTAATAAAA-TTTTTAAATCTGTAAAAAATAAAATGCCCATGTTTGCTATTCACT
AHGY01472396_[3375-3306]        CACTCTTTTCTCAACCTATGTATTACGTAATTTTTTTAACATTAGCTTTAATAAAAACTTT-AAATCTGTTCTTATCAAAAGGCGAGCCAGTGGAGGGCTTC
AHGY01356459_[1925-1993]        CACTCTTTTCTCAACCTATGTATTATATAATTTTTTTAACATTAGCTTTAATAAA--TTTTTTAAGTGTGTACTATTGACGTAAGAGAAGAGTCTTTAGGAG
AHGY01375402_[2038-2106]        CACTCTTTTCTCAACCTATGTATTATATAATTTTTT-AACATTCGCTTTAATAAAA-TTTTTAAATCTGCTCTGGCACTGCCTAGCTGCTAAGGAAACAACA
```

***Chelonia mydas* (green seaturtle)**

```
Utopia-1_CMy                    CAGAAACTTCTATGCTTAAACTCTGTACCGTTTTTTTTTATTT-CAACATCATCTTAATAAAATTATTAAA
U2 snRNA gene (human)           ---------------------------------ATCGCTTCTCGGCCTTTTGGCTAAGATCAAGTGTAGTATCTGTTCTTATCAGTTTAATATCTGATACGTCCTCTA
AJIM01177336_[10245-10311]      CAGAAACTTCTACGCTTAAACTCTGTACCATTTTTTTT--ATTTTATCATC---TTAATAAAATTATTAAATCTGTTTTTATCAATTTAATATCTGATATGTCCTTTA
AJIM01181910_[64763-64695]      CAGAGACCTCTATGCTTAAACTCCGTACCGTTCACTTT--TTTTAAACATCATCTTAATAAAATTTTTAAATCTGTT---ATCAGTTTAACATCCAATATGTCTTCTG
AJIM01200570_[11799-11868]      CAGAAACTTCTATGCTTAAACTCTGTACCATTTTTTTTTATTTT-AACATCATCTTAATAAAATTATTAAACATGTCCCCAAACTGGCTGGGGCTTGGAAATTTGGAA
AJIM01158325_[12338-12408]      CAGAAACTTCTATGCTTAAACTCTGTACCGTTTTCTTTTTACTTCAACATCATCTTAATAAAATTATTAAATATGTAAGCAGAGTCAGGATGAGCTCTACCCTGACAT
AJIM01160070_[41054-40984]      CAGAAACTTCTATGCTTAAACTCTGTACCGTTTTTTTTTTACTTCAACATCATCTTAATAAAATTATTAAATGTGTATCGTGCACTCTGCCTCGCTGAAGCCTCTGAC
AJIM01160344_[19216-19286]      CAGAAACTTCTATGCTTAAACTCTGTACCGTTTTCTTTTTACTTCAACATCATCTTAATAAAATTATTAAATTTGAATGCAGGGGTAATTAAATGTTATCCTTATTGA
AJIM01161296_[20382-20312]      CAGAAACTTCTATGCTTAAACTCTGTACCGTTTTCTTTTTACTTCAACATCATCTTAATAAAATTATTAAATCTTTTATTGGAGTGTTGGTGAAAGAGACAAACTTCT
AJIM01162312_[20702-20772]      CAGAAACTTCTATGCTTAAACTCTGTACCGTTTTCTTTTTACTTCAACATCATCTTAATAAAATTATTAAATCTGTAACTTGTTTGTATTGATGTGTAAAAGGAGAAG
AJIM01178159_[7714-7784]        CAGAAACTTCTATGCTTAAACTCTGTACCGTTTTCTTTTTACTTCAACATCATCTTAATAAAATTATTAAAACGGAGATTCCATGTTATGTGACTACAGAGAACTACA
AJIM01178599_[2691-2761]        CAGAAACTTCTATGCTTAAACTCTGTACCGTTTTCTTTTTACTTCAACATCATCTTAATAAAATTATTAAATTTGTTACCTTCAAAATGGGCCCCACTCAAAGATAGT
AJIM01179709_[6676-6606]        CAGAAACTTCTATGCTTAAACTCTGTACCGTTTTCTTTTTACTTCAACATCATCTTAATAAAATTATTAAATCTGTACTAACACAATTAGCAGCTTGCAAGTTTCACA
AJIM01135411_[808-878]          CAGAAACTTCTATGCTTAAACTCTGTACCGTTTTCTTTTTACTTCAACATTATCTTAATAAAATTATTAAATTTGAAAGCAGGAGTAATGAAACATTGTTATCCTGAC
AJIM01140351_[10874-10804]      CAGAAACTTCTATGCTTAAACTCTGTACTGTTTTCTTTTTATTTCAACATTATCTTAATAAAATTATTAAATCTGTGTCATGTGGCTGCCATCCATTCCTCATTAGCG
AJIM01141487_[16228-16158]      CAGAAACTTCTATGCTTAAACTCTGTACTGTTTTCTTTTTACTTCAACATCATCTTAATAAAATTATTAAATCTGTCTTTGCTGCATTCTGACAGCAATAACAAGATG
AJIM01145354_[27932-28002]      CAGAAACTTTTATGCTTAAACTCTGTACCGTTTTCTTTTTACTTCAACATCATCTTAATAAAATTATTAAATACTAAAAAGGACTACATACTGGCATACGTTTGCCTC
AJIM01152631_[28017-27947]      CAGAAACTTCTATGCTTAAACTCTGTACCATTTTCTTTTTATTTTAACATCATCTTAATAAAATTATTAAATCTGTGAGGAATGGAGAGCACAGATGACAGAACTCCT
AJIM01154293_[26202-26132]      CAGAAACTTCTATGCTTAAACTCTGTACCGTTTTCTTTTTACTTCAGCATCATCTTAATAAAATTATTAAATCTGCTTTAGCCTCTGCCTACATTGCATGGGATGGGC
AJIM01167965_[11320-11390]      CAGAAACTTCTATGCTTAAACTCTGTACCGTTTTCTTTTTATTTTAACACCATCTTAATAAAATTATTAAATCTGATACCATTAGTTCCTGAGCTATATAATGGAGAT
AJIM01172375_[12821-12751]      CAGAAACTTCTATGCTTAAACTCTGTACCGTTTTCTTTTTACTTCAACATTATCTTAATAAAATTATTAAATCTGTTCTTATTTACTTTACAGTTCAAAGCAAATGCA
AJIM01173728_[2474-2544]        CAGAAACTTCTATGCTTAAACTCTGTACCGTTTTCTTTTTACTTCAACATTATCTTAATAAAATTATTAAATCTGTAAAAACCTAGCATTCCCCCAGATTTAGAAATG
AJIM01174816_[727-797]          CAGAAACTTCTATGCTTAAACTCTGTACTGTTTTCTTTTTACTTCAACATCATCTTAATAAAATTATTAAATCCACCTCTCCAAGGGGCGGTAGCCAGGTCAACAGAA
AJIM01175831_[1235-1305]        CAGAAACTTCTATGCTTAAACTCTGTACCGGTTTCTTTTTACTTCAACATCATCTTAATAAAATTATTAAATATAAACACCTCTCCCACTACACCCCACTACCTAATG
```

```
Utopia-2_CMy                    ATGTACAGACACTCTTTTCCCAACCTATGTATTATA----TTTTTTTAACATTAGCTTTAATAAAA--TTTTTAAA
U2 snRNA gene (human)           --------------------------------------ATCGCTTCTCGGCCTTTTGGCTAAGATCAAGTGTAGTATCTGTTCTTATCAGTTTAATATCTGATACGTCCTCTA
AJIM01199495_[13952-13881]      ATGTACAGACACTCTTTTCTCAACCTGGGTATTGTATAA----TTTTAACATTAGCTTAATAATAAAATTTTTAAATTTGTTCTTATCAGTTTAATAGCTGATATGTCCTTTA
AJIM01150619_[4391-44320]       ATGTACAGACACGCTTTTCTTAACCTGTGTTCTGTATAA--TTTTTTAAAATTAGCTTTAAGAAAAA--TTTTAAATCTGTTCTTATCAGTTTAATATTTGATATGTTCTCTA
AJIM01188015_[27743-27812]      ATGTACAGTACCTTCCCTCTTAACTTGTGTAAATTTGA----TTTTAAACATTAACTTTAACAAAA--TTTTTAAATCTGTTCTTATCAGTTTAATATCTGATATGTTCTCTA
AJIM01193952_[38056-37985]      ATGTACAGACATTCTTTTCCCAACCTATGTATTATATAA--TTTTTTAACATTAGTTTTAATAAAA--TTTTTAAACAGTCACAAATAGGCTTTCAAGTTAAAATTCTCTTAA
AJIM01172207_[16127-16200]      ATGTACAGACACTCTTTTCTCAACCTATGTATTATATAATTTTTTTTAACATTAGCTTTAATAAAA--TCTTTAAATCCACCATGACAGGTCATCAGCAAGGACTGAACCCAC
AJIM01149038_[3493-3424]        ATGTACAGACACTCTTTTCTCAACCTATGTATTATAG---TTTTTTTAACATTAGCTTTCATAAAA--TTTTTAA-TGACAACTATCAATCCCAAGATATGTATGTGTGTGCG
AJIM01142111_[3314-3386]        ATGTACAGACACTCTTTTCCCAACCTGTGTATTATATATA-TTTTTTAACATTAGCTTTAATAAAA--TTTTAAAATATCCTTGAGGATCTGGGCCTTCCTCAACTGGGGCCT
AJIM01179914_[1877-1946]        ATGTACAGACGCTCTTTTCTCAACCTATGTATTATATAA--TTTTTTAACATTAGCTTTAATAAAA--TTTTTATATCCGTGCTGGCTGCTAGTGATCATCCCTTCATCCTCC
AJIM01181969_[61192-61119]      ATGTACAGACACTCTTTTCCCAACCTATGTATTATATCATTTTTTTAAACATTAGCTTTAATAAAA--CTTTTAAATCTATTATTGAGCAAACTTCTGTTGCTGAGAGAGAGA
AJIM01136059_[15384-15456]      ATGTACAAACACTCTTTTCTCAACCTATGTATTGTATCT-TTTTTTTAACATTAGCTTTAATAAAA--TTTTTAAATCAGAGATTGACCATTCCAGTAGCTGTAAACCCCAAG
AJIM01165963_[117336-117404]    ATGTACAGACACTCTTTTCCCAACCTATGTATTATATAC-TTTTTTTAATATTAGCTTTAATAAAA--TTTTATTGGACCAACTTCTGTTGGTGGAAGAGGGAGAAGCTTTGA
AJIM01188910_[2393-2465]        ATGTACAGACACTCTTTTCCCAACCTATGTATTATATAA-TTTTTTTAACATTAGCTTTAATAAAAA--TGTTAAATCTGTTCTTATCCACTGGATCACAAAGAATATTGCCT
AJIM01180576_[3034-2961]        ATGTACAGACACTCTTTTCCCAATCTATGTATTACATAATTTTTTTTAACATTAGCTTTAATAAAA--TCTTTAAATCTGTTTATTTTATCCGACTGCAGGTCTTAGCGAAAC
AJIM01173068_[9326-9398]        ATGTACAGACACTCTTTTCCCAACCTATGTATTATATAA-TTTTTTTAACATTAGCTTTAATAAAAAA--TTTAAAATTTGCTTCCACTTGTATCTCCCATGACAGAGGAACA
AJIM01186662_[56519-56450]      ATGTACAGACACTCTTTTCTCAACCTATGTATTATATA----TTTTTAACATTAGCTTTAATAACA--TTTTAAAATCTGTTTCTAGAAAAAGCGTTAATAGTGTATTGTTAG
AJIM01170399_[16319-16391]      ATGTACAGATACTCTTTTCTCAACCTATGTATTATATAA-TTTTTTTAACATTAGCTTTAATAAAAA--TTTTAAATCTGTTCTACACAGTGATCATCAATATTGTGCCATGT
AJIM01199173_[2741-2809]        ATGTACAGACACTCTTTTCCCAACCTATGTAATATATAA-TTTTTTTAACATTAGCTTTAATAAAA--TTTTAAGATATAAGAAAGAGGAAAAAATCATTCAAGCCCGGTTTT
AJIM01198253_[40703-40772]      ATGTACAGACACTCTTTTCTCAACCTGTGTATTATATAA----TTTTAACATTAGCTTTAATGAAA--TTTTTAAATATTGAATGCAGGGGTTGTGTAATGTTGTTATCTTCA
AJIM01191198_[9689-9618]        ATGTACAGACACACTTTTCCCAAGCTATGTATTATATAA--TTTTTTAACGTTAGCTTTAATAAAA--TTTTTAAATCTGTTCTTACAGTCCTTTGTACTTCATTATCCTTCT
AJIM01144150_[7559-7488]        ATGTATAGACACTCTTTTCCCAACCTATGTATTATATAA-TTTTTTCAACATTCGCTTTAATAAAA--TTTTTAA-TTTGTGTGTGTGCTTAAATGTTTCAAGGATTGGGGCC
AJIM01144782_[7804-7732]        ATGTACAGACACTCTGTCCCCAACCTATGTATTATATATA-TTTTTTAACATTAGCTTTAATAAAA--TGTTTAAATTTGTTCTTATCAGCACCTGTGGCTAAGCAGTTGAGA
AJIM01148114_[5775-5846]        ACGTACAGACACTCTTTTCCCAACCTATGGATTATATC-TTTTTTTTAACATTAGCTTTAATAAAA--TTTCTAATACAGCTTGTAAGATCCTTGCAGTAGGACCCTTGTCTT
AJIM01137305_[8261-8333]        ATGTACAGACAGTCTTTTCTCAACCTATGTATTATATAA-TTTTTTTAACATTAGCTGTAATAAAA--TTTTTAAAAATGAGCACACACTTGACTGCCTCAACTCCTTATCTC
```

***Pelodiscus sinensis* (Chenese soft shell turtle)**

```
Utopia-1_PSi                    GTTCGCCAGATGTGTTTTACTTGTTTACTGTATTATATTTGG-AAACATTCACTCAATAAAACTCGTTT--TA
U2 snRNA gene (human)           -----------------------------------ATCGCTTCTCGGCCTTTTGGCTAAGATCAAGTGTAGTATCTGTTCTTATCAGTTTAATATCTGATACGTCCTCTA
AGCU01033222_[17349-17319]      -----------------------------------TGTTCGC-AAACATTCTCTCAATAAA---CG-TC--AATCTGTTCTTATCGGTTTAATATCTGATATGATCTCTA
AGCU01093769_[7350-7415]        -----------------------TTTGCTATCAATTTATTTC-AAACATTTGCTTAAACAAACATT-TC--TATCTGTTCTTATGAGTTTAATATCTGATATGTTCTCTG
AGCU01072766_[3352-3397]        --------------------------------------------AACATTCGCTCAATAAACTCTG-TC--AACCTGTTCTTATCAGTTTAGTAGCTGATATGTTCTATC
AGCU01041580_[1764-1707]        GCTCCCCAGATGTATTTTACTTGTTTACTGTATTTTATTTGA-AAACATTCACTCAATAAAACTCGTTT--AATCTGTTCTCCCCCGCCCCCGGCACAATGGGGAAAATA
AGCU01168676_[823-758]          TATTTTACCTCCTGTTTGTAAACTT-ACTGTGATCTGTTTG--AAACATTCGCTAAATAAAATTAGTTTTAAATCTGTTCTTATCAGTTTAAGATAAGAACAGATACTAC
AGCU01036331_[3350-3299]        -------AGATGCATTTTACTTGTTTAGTGTACTATA---GA--ATGCCTCACTTATTAAAACT---------TCAAAAGTGCCTGGAAACAAGGTGTATTGATAGTATT
AGCU01046927_[4915-4979]        ------------------------TAATGTGATTTTATTTTG-AAACATTTGCTCAATAAAACTTG-TC--AATCTGTTCTTATCAGTTTAATATCTTGTCTAATCTAAC
AGCU01127228_[13081-13143]      -----------------------TTATGTATTGTTTGTCTTTAAAAATTCT-CTAAATAAA-CGT---C--AATCTGTTCTTATCAGTTTAATATCTGATATGCTGGGAG
AGCU01113081_[1233-1184]        -------------------------------------ATTTG-AAACATTTGCTAAATAAAATTTG-TC--AATCTGTTCCTATCAGTTTATTACCTTACTTACACAGTG
AGCU01131182_[9904-9865]        -------------------------------------ATTTGTAAACATTCGCTCAATAAAACTTT-TT--AATCTGT-CCAAAGAACAGCCTTTTGTGATCCATGATAA
AGCU01166387_[38747-38701]      -------------------------------------ATTTG-AAACATTCGCTTAATAAAATTTG-TC--AATCTGTTCTCAGTTTATGCTGTTTGGGCTTGCAGGGTC
AGCU01003314_[44734-44781]      ---------------------------------------TGG-AAACATTCACTTAATAGAACTTTTTTTCTACATTTTATCATTAATGTGTACTGTACAGTAGAGCCTG
AGCU01039548_[12145-12099]      -----------------------------------------G-ACACATTCACTCAATAAAACTTT-TC--TATCTCTTGCCCTATCTGTTTCAATCTACCGCCTGCAAG
AGCU01039548_[12614-12660]      -----------------------------------------G-ACACATTCACTCAATAAAACTTT-TC--TATCTCTTGCCCTATCTGTTTCAATCTACCGCCTGCAAG
AGCU01054955_[6981-7023]        -------------------------------------------AAACATTCACTTAATAAACTTTG-TC--AATCTGTTCT-ATCAGTTTGTAAACTAGAGTATTTGCCA
AGCU01046144_[7674-7629]        -------------------------------------------AAACATTTGCTAAATAAA-CGTG-CC--AATCTGTTCTTATCAGTTTAATAAATTATCACTATTCAT
```

***Anolis carolinensis* (green anole)**

Utopia-1_ACar
The 3' terminus is uncertain.

***Alligator mississippiensis* (alligator)**

```
Utopia-1_AMi                    GTATTTCCCTACCGGCTTTGTCATCTTTTT---TGGATTCACAATCCTAAACATCTACTAATAAAAGTCAA
U2 snRNA gene (human)           ---------------------------------ATCGCTTCTCGGCCTTTTGGCTAAGATCAAGTGTAGTATCTGTTCTTATCAGTTTAATATCTGATACGTCCTCTA
AKHW01106026_[4671-4603]        GTTTTTCCCTATCGGCTTTGTCACCTTTT----TGGATTTACAATCCTTAACATCTACTAATAAAAGTCAATCTGTTCTTATCAGTTTAATATCTGATATGTCCTCGA
AKHW01071278_[15208-15138]      GTATTTCCCTATTAGCTTTGTCATCTTTTTT--TGGATTTACCATCCTTACCATCTACTAATAAAAGTCAATCTGTTCTTATCAGTTTAATCGCTGATACGTCCTCGA
AKHW01001837_[23597-23531]      GTATTTCCCTACTGACTTTGTCATTTTT-----TCA-TCCAGGACTCTTAACATCCACTAATAAAATTCAATCTGTTCTTCAATAAGCATATTCAATTACAATAATGT
AKHW01003250_[430-363]          GTATTTCCCTTATGGCTTTGTCATTTTTT----TGG-TTTATGACTCTTAACATTTTCTAATAAAATTCAATCTGCTCTCTGCAGACACAGAGAACAGAAGAAAGAGG
AKHW01001308_[306-374]          GTATTTCCCTACCGGCATTGTGAATCCAA----AGAGATGACAATCCTAAGCAAGTACTAATAAAAGTCAATCTGTTAGATTTAACTACCTTAGCTTAAACCAGTCTA
AKHW01000355_[95328-95370]      ------------------------TTTT-----TAA-TTCATGACTCTTAACATCTACTAATAAAATTCAATCTGTTCTCCTCTGTTCCTTTTTCTGCCCTTACTGTC
AKHW01001586_[623-691]          GTATTTCCCTACTGGCTTTGTTATCTTTTT---CGG-TTCATAACTCCTAACATCTATTAATAAAACTCAGTCTGTTTCTTTCCCAGCCCTGACAGAGGGTATTTGGT
AKHW01000122_[29406-29334]      GTATTTCCCTATCGGCTTTGTCATCTTTTTTTTTGGATTTACCATCCTTACCATTTACTAATAAAAGTCAGTCTGTTTATTAAGATGACGATTTATCTGAAAAGTAAA
AKHW01000541_[30572-30505]      GTATTTCCCTACTGGCTTTGTCATCTCTT----TGG-TTCATGACTCTTAAAATCCACTAATAAAATTCAATCTGTCTGGCTCCTCACTCCGAAGGAAGTAGTGGAGG
AKHW01001395_[142-210]          GTATTTCCCTACTGGCTTTGTCATCTTTT----TGGATTCACAATCCTAAACATCTGCTAATAAAATTCAATCTGTCCTGGGCATCAGTGTCTCACACCACCATTAAG
AKHW01002496_[1384-1313]        GTATTTCCCTACTGGCTTTGTCATCTTTTTTT-TGGATTCACAATCCTAAACATTTACTAATAAAAGTCAATCTGCTACCCAGGTATCTGCTACTCAGGTATAATATA
AKHW01000659_[9766-9836]        GTATTTCCCTACCAGCTTTGTCAGCTTTTTT--TGGATTCACAATCCTAAACATTTACTAATAAAAGTCAATCTGACATACCTGATTTGGGTCAAACTGGTTTATGCG
AKHW01003547_[1946-2014]        GTATTTCCCTACTAGCTTTGTCATCTTTT----TGGATTCATAACTCTTAACATCTACTAATAAAATTCAATCTGCATTTGAAAAACAAACATTTTCAAAATAAAACC
AKHW01002853_[23146-23211]      GTATTTCCATACTGGCTTTGTCATCTTTT----AGGAGTCACAATCCTAAACATCT---AATAAAATTCAATCTGACTTAACTGGTTTAGGTCAAACCCGTTTATGGA
AKHW01003559_[11943-11876]      --ATTTCCCTACCGGCTTTGTCATCTTTTT---TGGATTCACAATCCTAAACATCTACTAATAAAAGTCAATCAGACTTAAACAACTGAGGTCAAACCAGTTCATGGA
AKHW01000061_[15829-15759]      GTATTTCCCTACCGGCTTTGCCATCTTTTTC--TGGATTCACAATCCTAAACATTTACTAATAAAAGTCAATCTAACAGACCACATGCTGGGTTTGTGCTGAACCAAC
AKHW01000780_[643-712]          GTATTTCCCTACCAGCTTTGTCATCTTTTTT--TGGATTCACAATCCTAAACATCTACTAATGAAAGTCAATATTTCCTTCAGGTTATGAAGTTTTGGGGGTGAGCAG
AKHW01003465_[1399-1466]        -TATTTCCCTACTGGCTTTGTCATCTTTTT---TGAATTCACAAGCCTAAACATCTACTAATAAAAGTCAATATGATCAAAATGGATTAATCCATGGCTGACCTAATA
AKHW01000202_[10335-10267]      GTATTTCCCTACTGGCTTTGTCAGCTTTTTT--TG-ATTCACAATCCTAAACATTTACTAATAAAAGTCAATTTTTGGCACCAAGGGGAGGTTGTTAGAGCTGGCCCT
AKHW01000199_[28509-28577]      GTATTTCCCTATCGGCTTTGTCATCTTTT----TGGATTTACAATCCTTAACATCTACTAATAAAAGTCAATCAGTTTTATTGGTTTGGGTCAAACTGGTTTATGAAA
AKHW01002342_[88455-88520]      GTATTTCCCTACTGGCTTTGTCATCTTTTT---TGGATTCACAATCCTAAACATTTACTAATAAAAGTC--CTGTGGGCCACTCCCCCTGCCCTGGTGTTTTGTTGTG
AKHW01002342_[86669-86734]      GTATTTTCCTACCGGCTTTGTCAGCTTTTT---TGGATTCACAATCCTAAACATTTACTAATGAAATTC--CTGTGGTCCACCTCCACAAGCTGGATTCATCCCTGCT
AKHW01002720_[27509-27441]      GTATTTCTCTACTAGCTTTGTCATCTTTT----TGGATTCACAATCCTAAACATCTACTAATAAAATTCTATCAGACTTAACTGATTTGGATCAAACCAGTTTATGAA
AKHW01001547_[31667-31609]      GTATTTCCCTACTGGCTTTGTCAGCTTTTT---TGGATTCACAATCCTAAACATTTACTAATGTATAAAAAGGAGGCAAGTATTTCTATTTCAAATTAGAGTTGTTTT
AKHW01000909_[6048-6113]        GTATTTCCCTACTGGCTTGTCATCTTTT-----TGGATTCACAATCCTAAACATCTACTAAAAAAAATTCAAGCTGCTTCTCCTTTTCTCTTTTTTTAAGAACCTATGT
AKHW01002859_[10096-10029]      GTATTTCCCTACTGGCTTTGTCATATTTT----TGGATTCACAATCCTAAACATTTACTGATAAAATTCAGTTTGCTGCACTGGTCTGTTCCCTGAACCAAGATCACT
AKHW01003549_[8159-8221]        -TATTTCCCTACTGGCTTTGTCAGGTTT-----TGGATTCACAATCCTAAACATTTACTAATAAAAGTCCCTAACTGGACGTGAAGCTCTTTGGACTTGCTGAAGGAG
AKHW01000382_[117-177]          GTATTTCTCTACTGACTTTGTCATCTTTT----TGGATTCACAATCCTAAACATATACTAATAAA-TT---TCTATCAGATTTAACTGATTAAGGTCAAACTGATTTA
AKHW01001034_[9714-9776]        ---TTTCCCTACCAGCTTTGTCATCTGTTT---TGGATTCACAATTCTAAACTTCTACTAATA----TCAGTCTTAAATGATTTGGGTCAAACTGGTTTATGCAATGC
AKHW01002871_[7078-7146]        GTATTTCCCTACTGGTTTTGTCATCTTTTT---AGG-TTCATAACTCTTAACATCTACTAATAAAATTCAGTCCTCTCTGTGATCTCACAGTCTACAACGTTGTATTT
AKHW01003564_[5941-5876]        GTATTTCCCTTATGGCTTTGTCATCTTTT----TGG-TTCATGATTCTTAACATCTACTAATAAAATTCAAACTCAGAGATCCAATCAAAGACCCACAACTGGACATT
```

Strongylocentrotus purpuratus **(purple sea urchin)**

```
Utopia-1_SP                     CTCCAACTGGAGACCTACATAATTTGTTAAAATGATATAAATATTTCGAAGATGAAATTATTATTAATAAA
U2 snRNA gene (human)           ---------------------------------ATCGCTTCTCGGCCTTTTGGCTAAGATCAAGTGTAGTATCTGTTCTTATCAGTTTAATATCTGATACGTCCTCTA
AAGJ02140537.1_[7362-7431]      CTCCAACTGGAGACCTACATAATTTGTTAAAATGATATAAATATTTCGAAGATGAAATTATTATTAATAAATCTGTTCTTTTCAGCTTAATATCTGAAACGCGACTCA
AAGJ02154997.1_[953-884]        CTCCAACTGGAGACCTACATAATTTGTTAAAATGATATAAATATTTCGAAGATGAAATTATTATTAATAAATCTGTTCTTTTCAGCTTAATATCTGAAACGCGACTCA
AAGJ02119355.1_[355-419]        CTCCAACTGGAGACTTACATAATGTTGTTAAATGATATAACTATTTCAAAGATGAAAATATTATTTAAAAATCTGTTCTTTTCAGCTTAATATCTGAAACCCGACTCA
AAGJ02131711.1_[3013-3082]      CTCCAACTGCAGACTTATATCATTGTTGCTAAATGATATAACTGTTCCGAAGATGAAATATTATTAATAAATCTGTTCTTTTTAGCTTAACATCTGGAATGCAACTCA
AAGJ02137703.1_[7244-7175]      CTCCAACTGCAGACTTATATCATTGTTGCTAAATGATATAACTGTTCCGAAGATGAAATATTATTAATAAATCTGTTCTTTTTAGCTGAACATCTGGAATGCAACTCA
```

Lytechinus variegatus **(green sea urchin)**

```
Utopia-1_LV                     CTTTATACCGTTGGATCAACATA-TATGATTTGTAAAACTGTTATTTCTGAGTTTTTT---CTATGCTAATAAA
U2 snRNA gene (human)           ------------------------------------ATCGCTTCTCGGCCTTTTGGCTAAGATCAAGTGTAGTATCTGTTCTTATCAGTTTAATATCTGATACGTCCTCTA
AGCV01358106_[538-469]          CTTTATACCGTTGGATCAACATA-TATGATTTGTAAAACTGTTATTTCTGAGTTTTTT---CTATGCTAATAAATCTGTTCTTTTCAGCTTAATATCTGAAACGCGACTCA
AGCV01356300_[395-322]          CTTTATACCGTTGGATCAACATAATATGATTTGTTAAACTGTACCTACTGAGTTTTTTTTTATATGTTAATAAATCTCTCCTTTTCAGCATAATATCTGAAACGCGACTCA
```

Patiria miniata **(bat star)**

```
Utopia-1_PMi                    GAGGGTTTTTATTACTATTAATTTTGTTTACTCTTTGTAACTTGTTTGATTGAATATTTTAATAAACCAC
U2 snRNA gene (human)           --------------------------------ATCGCTTCTCGGCCTTTTGGCTAAGATCAAGTGTAGTATCTGTTCTTATCAGTTTAATATCTGATACGTCCTCTA
AKZP01115809_[1299-1368]        GAGGGTTTTTATTACTATTAATTTTGTTTACTCTTTGTAACTTGTTTGATTGAATATTTTAATAAACCACTCTGTTCTTTTCAGCTTAATATCTGAAACGCTGCTCA
AKZP01013404_[4021-3952]        GAGGGTTTTTATTACTATTAATTTTGTTTACTCTTTGTAACTTGTTTGATTGAATATTTTAATAAACCACTCTGTTCTTTTCAGGTACTGCCGGACGTGTGTCTCCA
```
