# Supplementary material for: Ancient Origin of the U2 Small Nuclear RNA Gene-Targeting Non-LTR Retrotransposons Utopia
Source: PLoS One. 2015 Nov 10;10(11):e0140084. doi: 10.1371/journal.pone.0140084 (PMC4640811; doi:10.1371/journal.pone.0140084)
Supplement: S3 Fig — The sequences of U2 snRNA genes are shown in uppercase. Unsequenced regions are shown by “n”. (PDF) [file pone.0140084.s003.pdf]

S3 Figure.

P. infestans

Accession no.	U2 Position		Sequence
(Intact U2)			
AATU01000141	4785-4887	1-103	ATACCTTCTCGGCCTCTTGGCTAAGATCAAGTGTAGTATCTGTTCTAATCATTGTGAAAACTGGTTTTCGCCTTTTGGCGGACTTGTT-CACTCGCATTTTTGG
AATU01000153	2443-2545	1-103	ATACCTTCTCGGCCTCAAGGCTAAGATCAAGTGTAGTATCTGTTCTAATCAGTGTGAAAACTGGTTTTCGCCTTTTGGCGGACTTTTT-CACTCGCATTTTTGG
AATU01001281	24789-24891	1-103	ATACCTTCTCGGCCTCAAGGCTAAGATCAAGTGTAGTATCTGTTCTAATCAGTGTGAAAACTGGTTTTCGCCTTTTGGCGGACTTTTT-CACTCGCATTTTTGG
AATU01001299	7335-7233	1-103	ATACCTTCTCGGCCTCAAGGCTAAGATCAAGTGTAGTATCTGTTCTAATCAGTGTGAAAACTGGTTTTCGCCTTTTGGCGGACTTTTT-CACTCGCATTTTTGG
AATU01001278	26548-26650	1-103	ATACCTTCTCGGCCTTCAGGCTAAGATCAAGTGTAGTATCTGTTCTAATCAGTGTGAAAACTGGTTTTCGCCTTTTGGCGGACTTTTT-CACTCGCATTTTTGG
AATU01000193	222688-222790	1-103	ATACCTTCTCGGCCTCCAGGCTAAGATCAAGTGTAGTACCTGTTCTAATCAATGTGAAAACTGGTTTTCGCCTTTTGGCG-AGTTTTTTCACGCGCATTTTTGG
AATU01000137	7009-7105	1-96	ATACCTTCTCGGCCTCAAGGCTAAGATCAAGTGTAGTATCTGTTCTAATCAGTGTGAACACTGGTTTTGCACCGTTTGGCGGACTTTTTCACTCGCAnnnnnnn
Utopia-1_PI			
AATU01001281	4723-4787	39-103	tacggagaggttactaactggtaaatacgaacacatatTCTGTTCTAATCAGTGTGAAAACTGGTTTTCGCCTTTTGGCGGACTTTTT-CACTCGCATTTTTGG
AATU01000138	8818-8882	39-103	tacggagaggttactaactggtaaatacgaacacatatTCTGTTCTAATCAGTGTGAAAACTGGTTTTCGCCTTTTGGCGGACTTTTT-CACTCGCATTTTTGG
AATU01000163	3038-3102	39-103	tacggagaggttactaactggtaaatacgaacacatatTCTGTTCTAATCAGTGTGAAAACTGGTTTTCGCCTTTTGGCGGACTTTTT-CACTCGCATTTTTGG
AATU01001283	28487-28551	39-103	tacggagaggttactaactggtaaatacgaacacatatTCTGTTCTAATCAGTGTGAAAACTGGTTTTCGCCTTTTGGCGGACTTTTT-CACTCGCATTTTTGG
AATU01001290	2178-2114	39-103	tacggagaggttactaactggtaaatacgaacacatatTCTGTTCTAATCAGTGTGAAAACTGGTTTTCGCCTTTTGGCGGACTTTTT-CACTCGCATTTTTGG
AATU01001290	8718-8654	39-103	tacggagaggttactaactggtaaatacgaacacatatTCTGTTCTAATCAGTGTGAAAACTGGTTTTCGCCTTTTGGCGGACTTTTT-CACTCGCATTTTTGG
AATU01001290	15271-15207	39-103	tacggagaggttactaactggtaaatacgaacacatatTCTGTTCTAATCAGTGTGAAAACTGGTTTTCGCCTTTTGGCGGACTTTTT-CACTCGCATTTTTGG
AATU01001295	2245-2181	39-103	tacggagaggttactaactggtaaatacgaacacatatTCTGTTCTAATCAGTGTGAAAACTGGTTTTCGCCTTTTGGCGGACTTTTT-CACTCGCATTTTTGG
AATU01000133	1681-1617	39-103	tacggagaggttactaactggtaaatacgaacacatatTCTGTTCTAATCAGTGTGAAAACTGGTTTTCGCCTTTTGGCGGACCTTTT-CACTCGCATTTTTGG
AATU01000132	861-927	37-103	tacggagaggttactaactggtaaatacgaacacaTA-TCTGTTCTAATCAGTGTGAAAACTGGTTTTCGCCTTTTGGCGGACTTTTT-CACTCGCATTTTTGG
Utopia-2_PI			
AATU01000141	3920-3984	39-103	tggtaacgcgtaagccaaatggtaatacacaaaactttTCTGTTCTAATCAGTGTGAAAACTGGTTTTCGCCTTTTGGCGGACTTTTT-CACTCGCATTTTTGG
AATU01001299	416-352	39-103	tggtaacgagtaagccaaatggtaatacacaaaactttTCTGTTCTAATCAGTGTGAAAACTGGTTTTCGCCTTTTGGCGGACTTTTT-CACTCGCATTTTTGG
AATU01000150	2395-2331	39-103	tggtaacgagtaagccaaatggtaatacacaaaactttTCTGTTCTAATCAGTGTGAAAACTGGTTTTCGCCTTTTGGCGGACTTTTT-CACTCGCATTTTTGG
AATU01000162	906-970	39-103	tggtaacgagtaagccaaatggtaatacacaaaactttTCTGTTCTAATCAGTGTGAAAACTGGTTTTCGCCTTTTGGCGGACTTTTT-CACTCGCATTTTTGG
AATU01000164	6292-6356	39-103	tggtaacgagtaagccaaatggtaatacacaaaactttTCTGTTCTAATCAGTGTGAAAACTGGTTTTCGCCTTTTGGCGGACTTTTT-CACTCGCATTTTTGG
AATU01001283	8036-8100	39-103	tggtaacgagtaagccaaatggtaatacacaaaactttTCTGTTCTAATCAGTGTGAAAACTGGTTTTCGCCTTTTGGCGGACTTTTT-CACTCGCATTTTTGG
AATU01001283	14921-14985	39-103	tggtaacgagtaagccaaatggtaatacacaaaactttTCTGTTCTAATCAGTGTGAAAACTGGTTTTCGCCTTTTGGCGGACTTTTT-CACTCGCATTTTTGG
AATU01001287	10393-10329	39-103	tggtaacgagtaagccaaatggtaatacacaaaactttTCTGTTCTAATCAGTGTGAAAACTGGTTTTCGCCTTTTGGCGGACTTTTT-CACTCGCATTTTTGG
AATU01004588	437-373	39-103	tggtaacgagtaagccaaatggtaatacacaaaactttTCTGTTCTAATCAGTGTGAAAACTGGTTTTCGCCTTTTGGCGGACTTTTT-CACTCGCATTTTTGG
AATU01000166	1842-1906	39-103	ttggtaacgagtaagccaaatggtaatacacaaaactcTCTGTTCTAATCAGTGTGAAAACTGGTTTTCGCCTTTTGGCGGACTTTTT-CACTCGCATTTTTGG
AATU01000169	821-885	39-103	ttggtaacgagtaagccaaatggtaatacacaaaactcTCTGTTCTAATCAGTGTGAAAACTGGTTTTCGCCTTTTGGCGGACTTTTT-CACTCGCATTTTTGG
AATU01000145	3811-3748	39-103	ttggtaacgagtgagccaaatggtaatacacaaaaattTCTGTTCTAATCAGTGTGAAAACTGGTTTTCGCCTTTTGGCGGACTTTT--CACTCGCATTTTTGG

AATU01000141	15247-15186	39-100	tgttaacgagtaagccaaatggtaatacacaaaactttTCTGTTCTAATCAGTGTGAAAACTGGTTTTCGCCTTTTGGCGGACTTTTT-CACTCGCATTTT-GG
AATU01000143	7828-7764	39-103	tgttaacgagtaagccaaatggtaatacacaaaactttTCTGTTCTAATCAGTGTGAAAACTGGTTTTCGCCTTTTGGCGGACTTTTT-CACTCGCATTTTTGG
AATU01000149	499-435	39-103	tgttagcgagtaagccaaatggtaatacacaaaactttTCTGTTCTAATCAGTGTGAAAACTGGTTTTCGCCTTTTGGCGGACTTTTT-CACTCGCATTTTTGG
AATU01001290	21770-21706	39-103	ataacgggtacaccggatggtaaatatacaaaaccttcTCTGTTCTAATCAGTGTGAAAACTGGGTTTCGCCTTTTGGCGGACTTTTT-CACTCGCATTTTTGG
Utopia-3_PI			
AATU01001281	11703-11767	39-103	ataacgggtacaccggatggtaaatatacaaaaccttcTCTGTTCTAATCAGTGTGAAAACTGGTTTTCGCCTTTTGGCGGACTTTTT-CACTCGCATTTTTGG
AATU01001283	21947-22011	39-103	ataacgggtacaccggatggtaaatatacaaaaccttcTCTGTTCTAATCAGTGTGAAAACTGGTTTTCGCCTTTTGGCGGACTTTTT-CACTCGCATTTTTGG
AATU01001290	28829-28765	39-103	ataacgggtacaccggatggtaaatatacaaaaccttcTCTGTTCTAATCAGTGTGAAAACTGGTTTTCGCCTTTTGGCGGACTTTTT-CACTCGCATTTTTGG
AATU01001291	772-708	39-103	ataacgggtacaccggatggtaaatatacaaaaccttcTCTGTTCTAATCAGTGTGAAAACTGGTTTTCGCCTTTTGGCGGACTTTTT-CACTCGCATTTTTGG
AATU01001292	6148-6085	39-103	gataacgggtacaccggatgtaaatatacaaaaccttcTCTGTTCTAATCAGTGTGAAAACTGGTTT-CGCCTTTTGGCGGACTTTTT-CACTCGCATTTTTGG
Utopia-4_PI			
AATU01001281	18264-18328	39-103	tacggagaggttactaactggtacataaaattacacatTCTGTTCTAATCAGTGTGAAAACTGGTTTTCGCCTTTTGGCGGACTTTTT-CACTCGCATTTTTGG
AATU01000137	6153-6217	39-103	tacggagaggttactaactggtacataaaattacacatTCTGTTCTAATCAGTGTGAAAACTGGTTTTCGCCTTTTGGCGGACTTTTT-CACTCGCATTTTTGG
AATU01001287	1076-1012	39-103	tacggagaggttactaactggtacataaaattacacatTCTGTTCTAATCAGTGTGAAAACTGGTTTTCGCCTTTTGGCGGACTTTTT-CACTCGCATTTTTGG
AATU01001283	1206-1270	39-103	tacggagaggttactaactggtacataaaattacacatTCTGTTCTAATCAGTGTGAAAACTGGTTTTCGCCTTTTGGCGGACTTTTT-CACTCGCATTTTTGG
AATU01016980	2273-2209	39-103	tacggagaggttactaactggtacataaaattacacatTCTGTTCTAATCAGTGTGAAAACTGGTTTTCGCCTTTTGGCGGACTTTTT-CACTCGCATTTTTGG
AATU01000138	2161-2223	41-103	tacggagaggttactaactggtacataaaattacacattgTGTTCTAATCAGTGTGAAAACTGGTTTTCGCCTTTTGGCGGACTTTTT-CACTCGCATTTTTGG
AATU01000138	5507-5569	41-103	tacggagaggctactaactggtacataaaattacacattgTGTTCTAATCAGTGTGAAAACTGGTTTTCGCCTTTTGGCGGACTTTTT-CACTCGCATTTTTGG
AATU01001295	8788-8726	39-103	tacggagaggttactaactggtacataaaattacacattaTGTTCTAATCAGTGTGAAAACTGGTTTTCGCCTTTTGGCGGACTTTTT-CACTCGCATTTTTGG
Utopia-5_PI			
AATU01010946	457-393	39-103	tggtaacgagtaagccaaatggtaatacacaaaactttTCTGTTCTAATCAGTGTGAAAACTGGTTTTCGCCTTTTGGCGGACTTTTT-CACTCGCATTTTTGG
AATU01000164	38-102	39-103	ntcggtacgagtaagccaaatggtaatacacaaaactcTCTGTTCTAATCAGTGTGAAAACTGGTTTTCGCCTTTTGGCGGACTTTTT-CACTCGCATTTTTGG
(Flagmented U2)			
AATU01001286	1734-1635	4-103	tggCCTTCTCGGCCTCAAGGCTAAGATCAAGTGTAGTATCTGTTCTAATCAGTGTGAAAACTGGTTTTCGCCTTTTGGCGGACTTTTT-CACTCGCATTTTTGG

P. sojae

Scaffold	U2 Position		Sequence
(Intact U2)			
scaffold_72	396777-396879	3-105	ATACCTTCTCGGCCTTT-GGCTAAGATCAAGTGTAGTATCTGTTCTAATCAGTGTGAAAACTGGTTTCCCTGCTTTTGGTGGGGTCTTTTTCACATTTATTTTTGG
scaffold_55	453757-453859	3-105	ATACCTTCTCGGCCTTT-GGCTAAGATCAAGTGTAGTATCTGTTCTAATCAGTGTGAAAACTGGTTTCCCTGCTTTTGGTGGGGTCTTTTTCACATTTATTTTTGG
scaffold_72	329674-329570	1-104	GGACCTTCTCGGCCTTTTGGCTAAGATCAAGTGTAGTATCTGTTCTAATCAGCGTGAAAACTGGTTTCCCTGCTTTTGGTGGGGTCTTGTTCACATTCATTTTTGC
scaffold_892	7495-7391	1-104	GGACCTTCTCGGCCTTTTGGCTAAGATCAAGTGTAGTATCTGTTCTAATCAGCGTGAAAACTGGTTTCCCTGCTTTTGGTGGGGTCTTGTTCACATTCATTTTTGC
scaffold_370	132-28	1-104	GGACCTTCTCGGCCTTTTGGCTAAGATCAAGTGTAGTATCTGTTCTAATCAGCGTGAAAACTGGTTTCCCTGCTTTTGGTGGGGTCTTGTTCACATTCATTTTTGC
scaffold_370	4881-4781	1-104	GGACCTTCTCGGCCTTTTGGCTAAGATCAAGTGTAGTATCTGTTCTAATCAGCGTGAAAACTG-TTTCC-TGCTTTTG-TGGG-TCTTGTTCACATTCATTTTTGC
scaffold_25	787113-787217	1-105	GGACCTTCTCGGCCTTT-GGCTAAGATCAAGTGTAGTATCTGTTCTAATCAGTGTGAAAACTGGTTTCCCTGCTTTTGGTGGGGTCTTTTTCACATTCATTTTTGG

scaffold_36	5634-5738	1-105	GGACCTTCTCGGCCTTT-GGCTAAAATCAAGTGTAGTATCTGTTCTAATCAGTGTGAAAACTGGTTTCCCTGCTTTTGGTGGGGTCTTTTTCACATTTATTTTTGG
scaffold_72	334310-334271	1-40	GGACCTTCTCGGCCTTT-GGCTAAGATCAAGTGTAGTATCTnnnnnnnnnnnnnnnnnnnnnnnnnnnnnnnnnnnnnnnnnnnnnnnnnnnnnnnnnnnnnnnnn
scaffold_36	2685-2723	1-39	GGACCTTCTCGGCCTTT-GGCTAAGATCAAGTGTAGTATCnnnnnnnnnnnnnnnnnnnnnnnnnnnnnnnnnnnnnnnnnnnnnnnnnnnnnnnnnnnnnnnnnn
Utopia-1_PS			
scaffold_25	786672-786739	38-105	tacggagaggttactaactggtaaacacgaacacacatTCTGTTCTAATCAGTGTGAAAACTGGTTTCCCTGCTTTTGGTGGGGTCTTTTTCACATTTATTTTTGG
scaffold_72	326767-326700	38-105	tacggagaggttactaactggtaaacacgaacacacatTCTGTTCTAATCAGTGTGAAAACTGGTTTCCCTGCTTTTGGTGGGGTCTTTTTAACATTCATTTTTGG
scaffold_72	330075-330008	38-105	tacggagaggttactaactggtaaacacgaacacacatTCTGTTCTAATCGGTGTGAAAACTGGTTTCCCTGCTTTTGGTGGGGTCTTTTTCACATTCATTTTTGG
scaffold_72	334751-334684	38-105	tacggagaggttactaactggtaaacacgaacacacatTCTGTTCTAATCAGTGTGAAAACTGGTTTCCCTGCTTTTGGTGGGGTCTTTTTCACATTTATTTTTGG
scaffold_36	2244-2311	38-105	tacggagaggttactaactggtaaacacgaacacacatTCTGTTCTAATCAGTGTGAAAACTGGTTTCCCTGCTTTTGGTGGGGTCTTTTTCACATTTTTTTTTGG
scaffold_36	5193-5260	38-105	tacggagaggttactaactggtaaacacgaacacacatTCTGTTCTAATCAGTGTGAAAACTGGTTTCCCTGCTTTTGGTGGGGTCTTTTTCACATTTTTTTTTGG
scaffold_370	5280-5215	40-105	tacggagaggttactaactggtaaacacgaacacacattgTGTTCTAATCGGTGTGAAAACTGGTTTCCCTGCTTTTGGTGGGGTCTTTTTCACATTCATTTTTGG
Utopia-2_PS			
scaffold_25	772663-772730	38-105	acgcaacggactaaccggcaggtaatacattaaccctTCTGTTCTAATCAGTGTGAAAACTGGTTTCCCTGCTTTTGGTGGGGTCTTTTTCACATTCATTTTTGG
scaffold_25	777467-777534	38-105	acgcaacggactaaccggcaggtaatacattaaccctTCTGTTCTAATCAGTGTGAAAACTGGTTTCCCTGCTTTTGGTGGGGTCTTTTTCACATTCATTTTTGG
scaffold_25	761325-761392	38-105	acgcaacggactaaccggcaggtaatacattaaccctTCTGTTCTAATCAGTGTGAAAACTGGTTTCCCTGCTTTTGGTGGGGTCTTTTTCACATTTATTTTTGG
scaffold_370	12786-12719	38-105	nnnnnnnggactaaccggcaggtaatacattaaccctTCTGTTCTAATCAGTGTGAAAACTGGTTTCCCTGCTTTTGGTGGGGTCTTTTTCACATTTATTTTTGG
(Flagmented U2)			
scaffold_25	773083-773160	1-78	GGACCTTCTCGGCCTTT-GGCTAAGATCAAGTGTAGTATCTGTTCTAATCAGTGTGAAAACTGGTTTCCCTGCTTTTGGccgcagccaacgccaccgtcgcggcgg
scaffold_25	761046-761008	67-105	gcgacggagtccccccggtctccgtcacccgactgcccctggtgtcttggctggggcgcttgtccccCCCTGCTTTTGGTGGGGTCTTTTTCACATTTATTTTTGG
(Unclassifiable)			
scaffold_25	790490-790429	44-105	nnnnnnnnnnnnnnnnnnnnnnnnnnnnnnnnnnnnnnnnnnnnCTAATCAGTGTGAAAACTGGTTTCCCTGCTTTTGGTGGGGTCTTTTTCACATTCATTTTTGG
scaffold_1116	1-44	61-104	nnnnnnnnnnnnnnnnnnnnnnnnnnnnnnnnnnnnnnnnnnnnnnnnnnnnnnnnnnnnnTGGTTTCCCTGCTTTTGGTGGGGTCTTGTTCACATTCATTTTTGC
scaffold_55	465075-465110	69-105	nnnnnnnnnnnnnnnnnnnnnnnnnnnnnnnnnnnnnnnnnnnnnnnnnnnnnnnnnnnnnnnnnnnnnnCTGCTTTTGGTGGGGTCTTTTTCCATTTATTTTTGG

P. ramorum

Scaffold	U2 Position		Sequence
(Intact U2)			
scaffold_1698	2882-2777	1-106	ATACCTTCTCGGCCTTTTGGCTAAGATCAAGTGTAGTATCTGTTCTAATCAGTGTGAAAACTGGTTTCC---GA-CGTTTTTCGTTGGTCTTTTTCACATTCATTTTTGG
scaffold_1672	232-337	1-106	ATACCTTCTCGGCCTTTTGGCTAAGATCAAGTGTAGTATCTGTTCTAATCAGTGTGAAAACTGGTTTCC---GA-CGTTTTTCGTTGGTCTTTTTCACATTCATTTTTGG
scaffold_323	6667-6562	1-106	ATACCTTCTCGGCCTTTTGGCTAAGATCAAGTGTAGTATCTGTTCTAATCAGTGTGAAAACTGGTTTCC---GA-CGTTTTTCGTTGGTCTTTTTCACATTCATTTTTGG
scaffold_196	7801-7906	1-106	ATACCTTCTCGGCCTTTTGGCTAAGATCAAGTGTAGTATCTGTTCTAATCAGTGTGAAAACTGGTTTCC---GA-CGTTTTTCGTTGGTCTTTTTCACATTCATTTTTGG
scaffold_76	21879-21774	1-106	ATACCTTCTCGGCCTTTTGGCTAAGATCAAGTGTAGTATCTGTTCTAATCAGTGTGAAAACTGGTTTCC---GA-CGTTTTTCGTTGGTCTTTTTCACATTCATTTTTGG
scaffold_76	2992-3097	1-106	ATACCTTCTCGGCCTTTTGGCTAAGATCAAGTGTAGTATCTGTTCTAATCAGTGTGAAAACTGGTTTCC---GA-CGTTTTTCGTTGGTCTTTTTCTCATTGAGTTTTGG
scaffold_271	19089-18980	1-106	ATACCTTCTCGGCCTTTTGGCTAAGATCAAGTGTAGTATCTGTTCTAATCAGTGTGAAAACTGGTTTCCGGGGATCGTTTTTCGTTGGTCTTTTTCACATTCATTTTTGG
scaffold_196	8320-8411	1-93	ATACCTTCTCGGCCTTTTGGCTAAGATCAAGTGTAGTATCTGTTCTAATCAGTGTG-AAACTGGTTTCC---GA-CGTTTTTCGTTGGTCTTTTTCAnnnnnnnnnnnn
scaffold_271	13067-12993	1-75	ATACCTTCTCGGCCTTTTGGCTAAGATCAAGTGTAGTATCTGTTCTAATCAGTGTGAAAACTGGTTTCC---GA-CGTTnnnnnnnnnnnnnnnnnnnnnnnnnnnnnnn

scaffold_76	26417-26359	1-59	ATACCTTCTCGGCCTTTTGGCTAAGATCAAGTGTAGTATCTGTTCTAATCAGTGTG-AAActggtt-cc---ga-cnnnnnnnnnnnnnnnnnnnnnnnnnnnnnnnnnn
Utopia-1_PR			
scaffold_76	40051-39984	39-106	ttacggagaggttactaactggtaacacgaaaacacatTCTGTTCTAATCAGTGTGAAAACTGGTTTCC---GA-CGTTTTTCGTTGGTCTTTTTCACATTCATTTTTGG
scaffold_271	13548-13481	39-106	ttacggagaggttactaactggtaacacgaaaacacatTCTGTTCTAATCAGTGTGAAAACTGGTTTCC---GA-CGTTTTTCGTTGGTCTTTTTCACATTCATTTTTGG
Utopia-2_PR			
scaffold_271	6559-6492	39-106	aagccaaggattaaccggtcggtaatacacataactttTCTGTTCTAATCAGTGTGAAAACTGGTTTCC---GA-CGTTTTTCGTTGGTCTTTTTCACATTCATTTTTGG
Utopia-3_PR			
scaffold_76	33111-33044	39-106	taacgggtataccggatggtaaataaaataaaaacttcTCTGTTCTAATCAGTGTGAAAACTGGTTTCC---GA-CGTTTTTCGTTGGTCTTTTTCACATTCATTTTTGG
scaffold_271	19550-19483	39-106	taacgggtataccggatggtaaataaaataaacacttcTCTGTTCTAATCAGTGTGAAAACTGGTTTCC---GA-CGTTTTTCGTTGGTCTTTTTCACATTCATTTTTGG
scaffold_323	67-1	39-105	taacgggtataccggatggtaaataaaataaaaacttcTCTGTTCTAATCAGTGTGAAAACTGGTTTCT---GA-CGTTTTTCGTTGGTCTTTTTCACATTCATTTTTGn
Utopia-4_PR			
scaffold_1494	543-476	39-106	aagccaaggattaaccggtcggtaatacacataactttTCTGTTCTAATCAGTGTGAAAACTGGTTTCC---GA-CGTTTTTCGTTGGTCTTTTTCACATTCATTTTTGG
scaffold_1698	3343-3276	39-106	aagccaaggattaaccggtcggtaatacacataactttTCTGTTCTAATCAGTGTGAAAACTGGTTTCT---GA-CGTTTTTCGTTGGTCTTTTTCACATTCATTTTTGG
scaffold_196	42601-42668	39-106	aagccaaggattaaccggtcggtaatacacataactttTCTGTTCTAATCAGTGTGAAAACTGGTTTCC---GA-CGTTTTTCGTTGGTCTTTTTCACATTCATTTTTGG
scaffold_76	58827-58760	39-106	aagccaaggattaaccggtcggtaatacacataactttTCTGTTCTAATCAGTGTGAAAACTGGTTTCC---GA-CGTTTTTCGTTGGTCTTTTTCACATTCATTTTTGG
Utopia-5_PR			
scaffold_76	22360-22293	39-106	aagccaaggattaaccggtcggtaatacacataactttTCTGTTCTAATCAGTGTGAAAACTGGTTTCC---GA-CGTTTTTCGTTGGTCTTTTTCACATTCATTTTTGG
(Other Utopia elements)			
scaffold_76	17872-17805	39-106	aagccaaggattaaccggtcggtaatacgcataactttTCTGTTCTAATCAGTGTGAAAACTGGTTTCC---GA-CGTTTTTCGTTGGTCTTTTTCACATTCATTTTTGG
scaffold_76	26878-26811	39-106	aagccaaggattaaccggtcggtaatacacataactttTCTGTTCTAATCAGTGTGAAAACTGGTTTCC---GA-CGTTTTTCGTTGGTCTTTTTCACATTCATTTTTGG
scaffold_1551	488-555	39-106	aagccaaggattaaccggtcggtaatacacataactttTCTGTTCTAATCAGTGTGAAAACTGGTTTCC---GA-CGTTTTTCGTTGGTCTTTTTCACATTCATTTTTGG
(Fragmented U2)			
scaffold_76	958-1002	62-106	aatccagagagatgcgaaaagccccccatctagaaggcatgcacatattcacctactactaTGGTTTCC---GA-CGTTTTTCGTTGGTCTTTTTCACATTCATTTTTGG
(Unclassifiable)			
scaffold_323	16620-16591	76-106	nnnnnnnnnnnnnnnnnnnnnnnnnnnnnnnnnnnnnnnnnnnnnnnnnnnnnnnnnnnnnnnnnnnnnnnnnnnnnnnTTTCGTTGGTCTTTTTCAC-TTCATTTTTGG
