# Supplementary material for: Ancient Origin of the U2 Small Nuclear RNA Gene-Targeting Non-LTR Retrotransposons Utopia
Source: PLoS One. 2015 Nov 10;10(11):e0140084. doi: 10.1371/journal.pone.0140084 (PMC4640811; doi:10.1371/journal.pone.0140084)
Supplement: S4 Fig — The sequence accession numbers or scaffold numbers, and nucleotide positions are shown below each alignment. Sequences similar to the specific insertion site in the U2 genes are underlined. Nucleotides of Utopia insertions are colored in blue. TSDs are in red. Sequences representing the original uninserted state of the locus, such as the orthologous loci from turtles, birds or mammals and the consensus sequences for transposable elements, are shown if available. (PDF) [file pone.0140084.s004.pdf]

S4 Figure.

Crocodile-only

```
Utopia-1_AMi                                                    GCCCCGCACGCCGACCGCGT//CATCTACTAATAAAAGTCAA
Crocodile               CTAGCGTGGTAATACCAAGAGGCT--TCCTGGGGGCTTGGGCCCCGCACGCCGACCGCAT//CATCTACTAATAAAAGTCAATCTGATGCAGTAACATAAAAGAAGACAGGATCCCTGTCCT
Gharial                 CTAGTGTGGTAATACCAAGAGGCTGTACCTGGGGGCTTGGGC----------------------------TTTATTGTAGTATCTGATACAGTAACATAAAAGAAGACAGGATCCCTGTCCT
Alligator               CTAGTGTGGTAATGCCAAGAGGCTCTACCTGGGGGCTTGGGC----------------------------TTTATTATAGTATTTGATGCAGTAACATAAAAGAAGACAGGATCCCTGTCCT
```

Utopia-1_AMi 3470-4727; Crocodile scaffold-1153 49680-50981; Gharial scaffold4222 101480-101573; Alligator AKHW01099966 34205-34298

```
Utopia-1_AMi                                                    GGACCTTCCTGGAGAGGTTC//CATCTACTAATAAAAGTCAA
Crocodile               AAACAGTCAAGTTTTAGACTATACTGATCTGATTTCAAGTGGACCTTCCTGGAGAGGTTC//CATCTACTAATAAAAGTCAATCTGTTCTTTTGAGTGCATTAGCTTTCTCAGTTTCAATAC
Gharial                 AAACAGTCAAGTTATAGACTATACTGATCTGATTTCAAGTGTAC--------------------------------------TATGTTCTTTTGAGTGCATTAGCTTTCTCAGTTTCAGTAC
Alligator               AAACAGTCAAGTTTTAGACTATACCGATCAGATTTCAAGTGTAC--------------------------------------TATGTTCTTTTGAGTGCATTAGCTTTCTCAGTTTCAATAC
```

Utopia-1_AMi 3510-4727; Crocodile, scaffold-801 108378-107090; Gharial scaffold4443 64812-64729; Alligator, AKHW01046432 21237-21320

```
Utopia-1_AMi                                                    ACTTGGCCACCTTCCTCAGC//CATCTACTAATAAAAGTCAA
Crocodile               GGTGTAAATTAGCACAGGCCTGTTGGCTTCTAAAGAGTGTACTTGGCCACCTTCCTCAGT//CATCTACTAATAAAAGTCAATATATGTCATATTCACATTGAAAATTTGTATTTGTAATCC
Gharial                 GGTGTAAATTAGCACAGGCCTGTTGGCTTCTAAAGAGTGTACTT------------------------------------AATATATGTCATGTTCACATTGAAAACTTGTATTCGTAAGCC
Alligator               GGTGTAAATTAGCAAAGGTCTGTTGGCTTCTAAAGAGTGTATTT--------------------------------------AATATGTCATATTCACATTGAAAATTTGCATTTGTAATCC
```

Utopia-1_AMi 3297-4727; Crocodile scaffold-13599 95222-93718; Gharial scaffold14432 208303-208218; Alligator AKHW01097790 18824-18741

```
Utopia-1_AMi                                                    GACCGCGTCACCGTGACGCC//CATCTACTAATAAAAGTCAA
Crocodile               TTTGAAATTGTCCAAGTACTGGGTAAGGACAATATTTAGTGACTGCATCACCGTGACGCC//CATCTACTAATAAAAGTTAAATCTGGGTTAAATAAGCCATTCACTTCAGCTGCAGTCAGA
Gharial                 TTTGAAATTCTCCAAGTACTGGGTAAGGACAATATTTAGT---------------------------------------ATAACCTGGGTTAAATAAGCCATTCACTTCAGCTACAGTCAGA
Alligator               TTTATGAGATTTGAAATCCTCCAAGTACTGGGTAAGAACA------------------------------GTATTTAGAATAACCTGGGTTAAATAAGCCATTAACTTTAGCTACAGTCAGA
```

Utopia-1_AMi 3482-4727; Crocodile scaffold-18689 97743-99017; Gharial scaffold1629 15382-15300; Alligator AKHW01077819 5405-5314

```
Utopia-1_AMi                                                    TGAGAGAGATCCTGGAGGGA//CATCTACTAATAAAAGTCAA
Crocodile               AGCTTGGTACAGGATTGAATGTACTTCATGTTCTCAGGAATGAGAGAGATCCTGGAGGGG//CATCTTCTAATAAATGTCCATCACCATGGTATCTGGGTTCCTCAGTCAGCTGTAGTTGGG
Gharial                 AGCTTGGTACAGGAATGAGTGTAC--CATGTCC------------------------------------------------ATCACCATGGTATCTGGGTTCCTCAGTCAACTGGAGTTGGG
Alligator               AGCTTGGTACAGGAATAAATGTAC--CATGTCC------------------------------------------------ATCACCATGGTATCTGGGTTCCTCAGTCAACTGGAGTTGGG
```

Utopia-1_AMi 1731-4727; Crocodile scaffold-19659 224271-227380; Gharial scaffold31265 26792-26863; Alligator AKHW01072378 369-440

```
Utopia-1_AMi                                                    GACTTGGCCACCTTCCTCAG//CATCTACTAATAAAAGTCAA
Crocodile               TGTTGGCTAGAATTAATGTGTATTAATTCTTATTAATTAAGACTTGGCCACCTTCCTCAG//CATCTACTAATAAAAGTCAATCTGTTCATGTAATTGTTTCTTACCATTTATACTTTTATT
Gharial                 AGTTGGCTAGAATTAATGT-------------------------------------------------------------ATCATATTCATGTAATTGTTTCTTACCATTTATACTTTTATT
Alligator               TGTTGCTTAGAATTAATATAT-------------------------------------------------------------CTTATTCATGTAATTTTTTCTTACCATTTATACTTCTATT
```

Utopia-1_AMi 3296-4727; Crocodile scaffold-10922 69431-70939; Gharial scaffold21472 35789-35729; Alligator AKHW01096279 9823-9883

```
Utopia-1_AMi                                                    CCAGCAATGAGGTCCTGCAC//CATCTACTAATAAAAGTCAA
Crocodile               TCCCTATAACTAACATTGAGAAATTCCTCCCTGTGTAGTACCAGCAATGAGGTCCTGCAC//CATCTACTAATAAAAGTCAATCTGCTCCACATGTTTTCAAAGAGTATATAGTA-TGCACA
Gharial                 TCCCTATAATTAATATTAAGAAATTCTTCACTGTGTGATA------------------------------------------TCTGTTCCACAT-TTT-CAAAGGGTACAAAGTACTGCATA
Alligator               TCCCTATAACTAACA---------------CTGTGTAGTA------------------------------------------TCTGCTCCACATGTTTTCAAAGAGTATATAGT-CTGCACC
```

Utopia-1_AMi 3099-4727; Crocodile scaffold-20288 34012-32327; Gharial scaffold4537 156566-156643; Alligator AKHW01049057 12705-12641

```
Utopia-1_AMi                                                    TGCAACCAGCCCCGCACGCC//CATCTACTAATAAAAGTCAA
Crocodile               TAAAGCACTAATCGATCTAAATCCCATGGCATTCAAGTCATGCACCCAGCCCCGCACGCT//CATCTACTAATAAAAGTCAATCTGGACTCACTGCCAGATCCTTGTGGGGCAGGTCCAAAG
Gharial                 TAAAGCACTGATCGATCTAAATCCCATGGCATTCAAGTCATGGGCCC----------------------TGAGCATGTGTAATATGGACTCATTGCCAGATCCTTGTGGGGCAGGTCCGAAG
Alligator               TAAAGCACTGATCGATCTAAATCCCATGGCATTCAAGTCATGGGCCC----------------------TTAGCATGTGTAATATGGACTCACTGCCAGATCCTTGTGGGGCAGGTCCAAAG
```

Utopia-1_AMi 3462-4727; Crocodile scaffold-797 339931-341274; Gharial scaffold4577 133267-133168; Alligator AKHW01107663 13766-13865

Crocodile/Gharial-only

```
Utopia-1_AMi                                                    GGCGGGGACTTTGCCTCGCT//CATCTACTAATAAAAGTCAA
Crocodile               TCTATGGGTTCACTTTCAGGACTATGTTCCTGTTATAGTAGGCAGGG-CTTTGCCTCACT//CATCTACTAATAAAAGTCAATCTATTTCTGCAGCTTTGCTACTTTTTAACTGCTTTACCA
Gharial                 -CTATGGGTTCACTTTCAGGACTATGTTCCTGTTATAGTAGGCGGGG-CTTTGCCTTGCT//CATCTACTAATAAAAGTCAATCTATTTCTGCAGCTTTGCTACTTTTTAACTGCTTTACCA
Alligator               TCTATGGGTTCACTTCCAGGAGTATGTTCCTGTTATAGTA------------------------------------------TCTATTTCTGCAGCTCTGCTACTTTTTAACTGCTTTACCA
```

Utopia-1_AMi 3347-4727; Crocodile, scaffold-1377 20391-21821; Gharial scaffold14734 106128-107585; Alligator, AKHW01013097 10939-10860

```
Utopia-1_AMi                                                    GGATCGGGAGGCAGCCCACC//CATCTACTAATAAAAGTCAA
Crocodile               AGTTGTAGCACCAGAAATGTCCTTTATAATTTTTTAGTAGGGATCATGAGGCAGCCAACT//CATTTACTAATAAAAGTCAATCTGTACCTGTGCTGGGTAGAATTTATTTTTTATCTGATG
Gharial                 AGTTCTACCACCATAAATGCCCTTTATAATTTTTTAGTAAGGATCGCGAGGCAGCCGACT//CATTTACTAATAAAAGTCAATCTGTACCTGTGCTGGGTAGAATTTATTTTTTATCTGATG
Alligator               AGTTATAGCACTGTAAATGTCCTTTATCTTTTTTTAGTA-------------------------------------------TCTGTACCTGTGTTGGGTGGAATTTATTTTTTCTCTGATG
```

Utopia-1_AMi 3270-4727; Crocodile scaffold-11928 80471-78989; Gharial scaffold389 28073-29633; Alligator AKHW01089192 4412-4490

```
Utopia-1_AMi                                                    CCAGCAATGAGGTCCTGCAC//CATCTACTAATAAAAGTCAA
Crocodile               TCCCTATAACTAACATTGAGAAATTCCTCCCTGTGTAGTACCAGCAATGAGGTCCTGCAC//CATCTACTAATAAAAGTCAATCTGCTCCACATGTTTTCAAAGAGTATATAGTATGCACAG
Gharial                 TCCCTATAACTAACATTGAGAAATTCCTCCCTGTGTAGTACCAGCAACAAGGTCCTGCAC//CATCTACTAATAAAAGTCAATCTGCTCCACATGTTTTCAAAGAGTATATAGTATGCACAG
Alligator               TCCCTATAACTAACA---------------CTGTGTAGTA------------------------------------------TCTGCTCCACATGTTTTCAAAGAGTATATAGTCTGCACCG
```

Utopia-1_AMi 3099-4727; Crocodile scaffold-20288 34012-32327; Gharial scaffold4537 230894-238868; Alligator AKHW01049057 12705-12641

```
Utopia-1_AMi                                                    GCCTCGCTGTGGAGCCGAGC//CATCTACTAATAAAAGTCAA
Crocodile               ATGCCTTTGCAAACAGCTGCTTTGTACCCAGCTCCATATAGCCTTGCTGTGGAGCTGAGC//CATCTACTAATAAAAGTCAACCTTTTTGTAGGTGATGGAGGTGAAAGGAAAAGTAAAAAG
Gharial                 ATGCCTTTGCAAACAGCTGCTTTGTATCTAGCTCCATATAGCCTCGCTGTGGAGCTGAGC//CATCTACTAATAAAAGTCAATCTTTTTGTAGGTGATGGAAGTGAAAGGAAAAGTAAAAAG
Alligator               GTACCTTTGCAAACAGCTGCTTTGTACCTAGCTCCATGTA----------------------------------------TATCTTTTTGTAAGTGATGGAAGTGAAAGGAAAAGTAAAAAG
```

Utopia-1_AMi 3359-4727; Crocodile scaffold-20147 33090-34471; Gharial scaffold4950 120531-119139; Alligator AKHW01086296 29143-29061

```
Utopia-1_AMi                                                    GAGGTCCTGCACATCCCCTA//CATCTACTAATAAAAGTCAA
Crocodile               TGAGTAAAGAACATAATGTTGGTGTGTCCTGCACATCCCCGAGGTCCTGCACATCACCTA//CATTTACTAAAAAAAGTCATTCTGTCATTTTTGCCAAGGTGCAAGGGATCAGCTTAAAGA
Gharial                 TGAGTAAAGAACAAAATGTTGGTGTGTCCTGCACATCCCCGAGGTCCTGCACATCCCCTA//CATTTACTAATAAAAGTCATTCTGTCATTTTTGCCAAGATGCAATGGATCAGCTTAAAGA
Alligator               TGAGTAAAGAACAAAATGTTGGTGTGTTATTCTGTGAGAAGGATACTGCCTGTGTTATAA//GGCAGAAACAATCCAGCGCGCTATTCATTTTTGCCAAGATGCAATGGATCAGCTTAAAGA
```

Utopia-1_AMi 3507-4727; Crocodile scaffold-8701 224418-226108; Gharial scaffold7013 204970-203270; Alligator AKHW01075084 13209-13067

```
Utopia-1_AMi                                                    GCAGCCCACCAGACGTGACT//CATCTACTAATAAAAGTCAA
```

```
Crocodile             ACATTTGAATGTAATACAGCATAGGCTAAGGTATGAAGTCGCATCCAACCGGATGCAACT//CATCTATGAATAAAATTTAATCTATTAAAATAATG------TAAAATATGTTGAGTCAAT
Gharial               AAATTTGAATGTAACACAGCATAGGCTAAGGTATGAAGTCACATCCAACCAGATGCGACT//CATCCATGAATAAAATTTACTCTATTAAAATAATG------TAAAATATGTTGAGTCAAT
Alligator             AACTTTGAATTTAATACAGCATAGGCTAAGGTATAAATT-----------------------------------TAGTATAATCTATTAAAATTTTAAAATAAAATAATATGCTGAGTTAAT
```

Utopia-1_AMi 3280-4727; Crocodile scaffold-14009 427411-425836; Gharial scaffold8294 24075-22616; Alligator AKHW01083126 10991-11077

```
Utopia-1_AMi                                                  GTCGCCAAGTGCGCCTCCCT//CATCTACTAATAAAAGTCAA
Crocodile             AAATTACTTCTCGGCCTCTTGAAGGCTCAGATCAAGTGTAGTAGCTAAGTGCGCCTCCCT//CATTTATTAATAAAAGTCAATCTCTACCTAGTATGAGAAGTATTAAATAGTTATGATTTT
Gharial               AAATTACTTCTCAGCCTCTTGAAGGCTCAGATCAAGTGTAGTAGCCAAGTGCACCTCCCT//CATTTATTAATAAAAGTCAATCTCTATCTACTGTGAGAGGTATTAAATAGTTATGATTTT
Alligator             AAATCACTTATTGGCCTCTTGAAGGCTCAGATCAAGTGTAGTA---------------------------------------TCTCTACCTACTATGAGAAGTATTAAATAGTTATGATCTT
```

Utopia-1_AMi 2729-4727; Crocodile scaffold-18219 209819-207772; Gharial scaffold7387 49049-41877; Alligator AKHW01025445 2168-2250

```
Utopia-1_AMi                                                  TAGCCTTCTACTGCGCCCTC//ACATCTACTAATAAAAGTCAA
Crocodile             GAACCTGGTTGAATGTAGTATCAGACTTAACTGATTGAGTTAGCATTCTACTGCTCCCGC//GCATTTACTAATAAAGGTCAATCAGACTTAACTGATTGAACTTAAACCAGTTTATGGAAAT
Gharial               GAACCTGATTGAATGTAGTATCAGACTTAACTGATTGAGTTAGCATTCTACTGCTCCCGC//ACATTTACTAATAAAGGTCAATCAGACTTAACTGATTGAGCTCAAACCAGTTTATGGAACT
Alligator             GAACCTGGTTGAATGTAGTATCAGACTTAACTGATTGA---------------------------------------------------------------GCTCAAACCAGTTTATGGAACT
Chompy-8_Crp          AAACCTGGTTGAATGTAGTATCAGACTTAACTGATTGA--------------------//-----------------------------------------GCTCAAACCGGTTTATGGAACT
```

Utopia-1_AMi 1025-4727; Crocodile scaffold-19386 212836-216614; Gharial scaffold10282 26821-28573; Alligator AKHW01065950 23903-23961; Chompy-8_Crp 123-182

Crocodile/Gharial/Alligator-shared

```
Utopia-1_AMi                                                  TCTGCGGCACCGAGTACGAG//CATCTACTAATAAAAGTCAA
Crocodile             TGAATGTACTCCCTAATAATTGGAGTACATTAACATGTAGTTTGTGGCACCGAGTACGAG//CATCTACTAATAAAATTCAATCTATCCTGGTATTTGTGCATGAAGCTACAA-TGCCCAAC
Gharial               TGAATGTACTCCCTAATAATTGGAGTACATTAACATGTAGTTTGTGGCACCGAGTACGAG//CATCTACTAATAAAATTCAATTTATCCTGGTATTTGTGCATGAAGCTACAA-TGCCCAGC
Alligator             TGAATGTACTCCCTAATAATTGGAGTATATTAACATGTAGTTTGCGGCACTGAGTACGAG//CATCTACTAATAAAATTCAATCTATCCTGGTATTTGTGCATGAAGCTACAA-TGCCCAGC
Soft shell turtle     TGAGAGAACCTTCTAGTGATTGGAGAGCATTAAAATGTAC------------------------------------------ATTATCTTAGAATTTATGTATAAAGTTATAAGTGTCTAAC
Zebrafinch            TGAGCGCGA-CAGAAACAATTGCAGCTCATTAAAATGGAT------------------------------------------CATGTCCTGACAGTCATGCATAATGAC-CAAGT-CCCA-C
```

Utopia-1_AMi 1043-4727; Crocodile scaffold-1505 17573-13936; Gharial scaffold25094 111274-114906; Alligator, AKHW01107126 10086-13669; Soft shell turtle AGCU01102438 2782-2703; Zebrafinch ABQF01035931 95886-95811

```
Utopia-1_AMi                                                  AGGACCTTCCTGGAGAGGTT//CATCTACTAATAAAAGTCAA
Crocodile             TCATACATTA--TTATCGCTTCCCAGCCTACTGAAGGCTCAGGACCTTCCTGGAGAGGTT//CATCAACTAATAAAATTCAGTCTGACCCAGATTCCTTTGTATTGATCTGT------AGCT
Gharial               CCATACATTA--TTATCACTTCCCAGCCTATTGAAGGCTCACGACCTTCCTGGAGAGGTT//CATCTACTAATAAAATTCAGTCTGACCCAGATTCCTTTGTACTGATCTGT------AGCT
Alligator             CCATACATTG--TTATCGCTTCCTAGCCTTCTGAAGGCTCAGGACCTTCCTGGAGCAGTT//CATCTACTAATAAAATTCAATCTGACCCAGATTCCTTTGTATTGATCTGT------AGCT
Seaturtle             CCATTCTTTAGTTTATA---TACCAG------------------------------------------------------------AACCATATTCCTTTTTATTTATATCTTCTCAGAACT
```

Utopia-1_AMi 3509-4727; Crocodile scaffold-13582 11689-12931; Gharial scaffold16508 44161-42924; Alligator AKHW01073376 20797-19547; Seaturtle AJIM01142539 67919-67977

```
Utopia-1_AMi                                                  GCGGGGACTTTGCCTCGCTG//CATCTACTAATAAAAGTCAA
Crocodile             AAAGAATTTATGGGACAGCAATTTTATTTT----------GCGGGGACTTGGCCTCGCTG//CATCTACTAATAAAATTCAGTCTGTTCCTTTATTTAAGAGACAGTCACATGCCAAAAACG
Gharial               AAAGAATTTATGGGACAGCAATTTTATTTT----------GCGGGGACTTGGCCTCGCTG//CATCTA---ATAAAATTCAGTCTGTTCCTTTATTTAAGAGACAGTCACATGCCAAAAACG
Alligator             AAAGAATTTATGGGACAGCATTTTTTTTTTTTTTAATTTTGCGGGGACTTTGCCTTGCCG//CATCTA---ATAAAATTCAATCTGTTCCTTTATTTAAGAGACAGTGACATGCCAAAAAGG
Platypus              AACTAATCTATTGGTAGAAATTCTATTT------------------------------------------------------TGTGTGTCTTTATTTAGGAGA--GTGAAAGGCCAAAAACT
```

Utopia-1_AMi 3348-4727; Crocodile scaffold-20245 3200-4595; Gharial scaffold19401 14234-12830; Alligator AKHW01081697 4303-5101; Platypus AAPN01001335 768-833

```
Utopia-1_AMi                                       CTCATCCACAAGAAGGGAGACCCGACCGACC//ACATCTACTAATAAAAGTCAA
Crocodile             ATTGGGAGAG-TCCCAAATGACTGGAAAAGGGCAAATGTAGAAGGGAGACCCGACCAACC//ACATCTACTAATAAAATTCAAT-TGTATTTAGCAAAAGGAAGAAAAAGGATCCAGGGAACT
Gharial               ATTGGGAGAG-CCCCAAATGACTGGAAAAGGGCGAATGTAGAAGGGAGACCCGACCGACC//ACATCTACTAATAAAATTCAATCTGTATTTAGCAAAAGGAAGAAAAAGGATCCAGGGAACT
Alligator             ATTGAGAGAGGTCCCAAATGACTGGAAAAGGGCAAATGTAGAAGGGAAACCCGACCGACC//ACATCTACTAATAACATTCAATCTGTCTTTAGCAAAAGGAAAAAAAAGTATCCAGGGAACT
CR1-1_Croc            ATCAGGAGAGGTCCCAGATGATTGGAAAAGGGCAAA----------------------------------------CATAGTGCCCATATTTAAGAAAGGGAAAAAGGAGGATCCAGGGAACT
```

Utopia-1_AMi 2081-4727; Crocodile scaffold-16800 137390-134483; Gharial scaffold8291 103330-100653; Alligator, AKHW01029955 1857-1788 and AKHW01029954 82957-82897; CR1-1_Croc 2864-2946

```
Utopia-1_AMi                                                  TGGCGGCCCGCATCACCGAC//ACATCTACTAATAAAAGTCAA
Crocodile             AAGAGTTTCCTCACCTGTTTGGGACTGACTTGATATAGTATGGTGGCCCGCATCGTCAAC//ACATCTACTAAGAAAAGTCAATCTGTAATCTTCCCCATATTCCGTGGGCCTCTGGACAGGT
Gharial               AAGAGTTCCCTCACCTGTTTGGGACTGACTTGACGTAGTATGGCGGCCCACATTGCCAAC//ACATCTACTAAGAAAAGTCAGTCTGTTATCTTCCCCATATTCCCTGGGCCTCTGGACAGGT
Alligator             AAGAGATCCCTCACCTGTTTG---------------------------------------//ACATTTACTAATAAAAGTCAATCTGTTATCTTCCCCATATTCCCTGGGCCTCTGGACAGGT
CR1-12_Crp            AAGAGTACCCTCACCTGTTCGGGACTGATTTCATGTGG---------------------------------------------TCTGTTGTCTTCCCCATATTCCCTGGGCCTCTGGACAGGT
```

Utopia-1_AMi 2172-4727; Crocodile scaffold-22084 129502-126971; Gharial scaffold4456 26265-23682; Alligator AKHW01078040 6015-4077 (it has a deletion including the 5' insertion junction.); CR1-12_Crp 2250-2147

```
Utopia-1_AMi                                                  ATCGGGAGGCAGCCCACCAG//CATCTACTAATAAAAGTCAA
Crocodile             GACAGAGATGCCTGTTGTGCAGTTGGGAAGAGACTGTCATATCGCGAAACAACTGACTGG//CATCTAATAATAAAATTCAATCTATATTATGTATTCACATGGGAACTATGTGCCGCATAC
Gharial               GACAGAGATGCCTGTTGTGCAGTTGGGAAGAGACT--CATATCGCGAGACAACCAACTAG//CATCTAATAATAAAATTCAATCTATATTATGTATCCACATGGGAACTATGTGCCACATAC
Alligator             GACAGAGATGTCTGTTGTGCAGTTGGGAAGAGA----------------------ACTGG//CATCTAATAATAAAATTCAATCTATAATATGTATCCACATGGGAACTATGCACCACATAC
```

Utopia-1_AMi 3272-4727; Crocodile scaffold-20945 103821-102343; Gharial scaffold14756 52063-53538; Alligator AKHW01041984 20030-21506

```
Utopia-1_AMi                                                  GCCTTCCGCCTCCTGACCTG//CATCTACTAATAAAAGTCAA
Crocodile             GTCAGTTATGACCAGCATGGCACTTTATCTCCCTCTCATGGCCTTCCACCTCCCGACCTG//CATTTACTAATATAAGTCCTTGTCCCCATAAGTCAGTTATGACCAGCATAGCACTTTGTC
Gharial               TTCAGTTATGACCAGCATGGCACTTTGTCTCCCTCCTGTGGCCTTCCGCCTCCTGACCTG//CATTTACTAATATAAGTCCTTGTCCCCGTAAGTCAGTTATGACCAGCATGGCACTTTGTC
Alligator             ATCAGTTATGACCGGCATGGCACTTTGTCTCCTTCTCATGGCCTTCCACCTCCTGACCTG//CATTTACTA---TAAGTCCTTGTCCCCATAAGTCAGTTATGGCCAGCATGGCACTTTGTC
```

Utopia-1_AMi 3191-4725; Crocodile scaffold-2443 225114-223585; Gharial scaffold5168 147016-144742; Alligator AKHW01078492 20691-19104

```
Utopia-1_AMi                                                  CGAGGACCTTCCTGGAGAGG//CATCTACTAATAAAAGTCAA
Crocodile             -------------ATGGGATCCTGCCAACGTGTATTCTGCCAAGGACCTTCCTGGAGTGG//CATCTACTAATAAAATTAAATCTGTTTTTACGGTCCAGCTAACTAACTAGTTGAACGAGC
Gharial               CCAGAATCCATGAATGGGATTCTGCCAACATGTACTCTGCTAAGGACCTTCCTGGAGCAG//CATCTACTAATAAAATTAAATCTGTTTTTACGGTCCAGCTAACTAACTAGTTGAATGAGC
Alligator             CTGGAATCCATGAATGGGATTCTGCCAATGTGTATTCTGCCGAGGACCTTCCTGGAGTGG//CATCTACTAATAAAATTAAATCTGTTTTTAAGGTCCAGCTAACAAACTAGTTGGATGAGT
```

Utopia-1_AMi 3507-4727; Crocodile scaffold-4550 189096-187839; Gharial scaffold6743 71456-70182; Alligator AKHW01060224 8205-6923

```
Utopia-1_AMi                                                  CTCGCTGTGGAGCCGAGCCC//CATCTACTAATAAAAGTCAA
Crocodile             ACTACATGGTGAGGTACTGAGCGGCCAAAGTCCAAATGTACTAGCTGTGGAGCCGAGCCC//CATACACTAATAAAAGTAAATCTGTATTCTGCCCAGCCAGCTCTGCAGTCACTCAGTTCA
Gharial               ACTACATAGTGAGGTACCAAGCAGCCAAAGCCCAAATGTACTAGCTGTGGAGCCGAGCCC//CATGTACTAATAAAAGTAAATCTGTACTCCGCCCAGCCAGCTGTGCAGTCACTCAGTTCA
Alligator             ACTACACAGTGAGGTACTGAGCAGCCAAAGCCCAAATGTACTAGCTGTGGAGCTGAGCCC//CATTTACTAGTAAAAGTCAATCTGTACTCTGCCCAGCCAGCACTGCAGCCACTCAGTTCA
```

Utopia-1_AMi 3361-4727; Crocodile scaffold-982 248495-249921; Gharial scaffold10888 37438-38865; Alligator AKHW01013213 4638-4697 & AKHW01013214 4319-4378

```
Utopia-1_AMi                                                  GTGGCCCGGCTCACAGAGGA//CATCTACTAATAAAAGTCAA
Crocodile             CCCCATGAGGGTACACCGCCTTTTGGCTCAGCTCAAGTGTGTGGCCTGGCTTACAGAGGA//CATCTACTAACAAAATTCATTCTGCTCTAGCCAGTTTTTTAAAGGAAATAGTAAGTGTTG
Gharial               CCCCATGAGGGTACACCGCCTTTTGACTCAGCTCAAGTGTGTGGCCTGGCTCACAGAGGA//CATCTACTAATAAAATTCAATCTGCTCTAGCCAGTTTTTTAAAGGAAACAGTAAGTGTTG
```

```
Alligator               CCCCATGAGGATACACCACCTTTTGGCTCAGCTCAAGTGTGTGGCCCAGCTCACAGAAGA//
```

Utopia-1_AMi 3270-4727; Crocodile scaffold-11932 5199-1995; Gharial scaffold15809 56682-48263; Alligator AKHW01099609 13566-13507

```
Utopia-1_AMi                                                GGAGCCGAGCCCGCAACGCC//CATCTACTAATAAAAGTCAA
Crocodile               ATTATTTCCTATGATTGTTATGGTAAAATATTATGTATAAGGAGCCGAGCCCACAATGCC//CATCTACTAATAAAATACAATATGTTTGGCCTTACAAAGCATACCTCTGAGTAACTGTGT
Gharial                 ATTATTTCCTATGATTGTTATGGTAAAATATTATGTATAAGGAGCCGAGCCCGCAATGCC//CATCTACTAATAAAATTCAATATGTTTGGCCTTACAAAGCATACCTCTGAGTAACTGTGT
Alligator                                                                       //CATCTACTAATAAAATTCAATATGTTTGGCCTTACAAAGCATATCTCTGAGTAACTGTGT
```

Utopia-1_AMi 3369-4727; Crocodile scaffold-10887 110295-108923; Gharial scaffold25492 52915-51531; Alligator AKHW01019039 3306-3247

```
Utopia-1_AMi                                                GAGGTCCTGCACATCCCCTA//CATCTACTAATAAAAGTCAA
Crocodile               GAAAGATTCAAATCAGGTGTACTAATCAGGTGTACAAATCGAGGTCCTGCACATCCCCTA//TATCATTTACTAATATTAGTCTACACTACGGGGCTCACTTTATGTCAAAAGCAGGGACAG
Gharial                 GAAAGATTCAAATCAGGTGTACTAATCAGGTGTACAAATCAAGGTCCTGCACATCCCCTA//TATCATTTACTAATAATAGTCTACACTACAGGGCTCACTTTATGTCAAGAGCAGGGACAG
Alligator                                                                       //AAACATTTACTAATAATAGTCTACACTACAGGGCTCACTTTATGTCAACAGCAGGGATAG
```

Utopia-1_AMi 3107-4727; Crocodile scaffold-23077 65551-67170; Gharial scaffold32567 17633-15957; Alligator AKHW01067637 38806-38865

```
Utopia-1_AMi                                                GGCAGGGAGGTGCCAACGTC//CATCTACTAATAAAAGTCAA
Crocodile               CAGAGTCCTCTGCACTCAGCAACGTTTGCCCCCATTAGTAGATAGGGAGATGCCAACGTC//CATATACTAATAAAAGTCAATCTGTTGTGTTTAACCAGTAAATCAAGAAAATTTATCAGA
Gharial                 CAGAGTCCTCTGCACTCAGCAAGGTTTGCCCCCATTAGTAGATAGGGAGGTGCCAACGTC//CATATACTAATAAAAGTCAATCTGTTGTGTTTAACCAGTAAATCAAGAAAATTTATCAGA
Alligator                                                                       //CATGTACTAATAAAAGTCAATCTGTTGTGTTTAACCAGTAAATCAAGAAAATTAAACAGA
```

Utopia-1_AMi 3129-4727; Crocodile scaffold-16976 6507-8136; Gharial scaffold7495 23441-25995; Alligator AKHW01011401 86534-86593
