# Supplementary figures and images for: Ancient Origin of the U2 Small Nuclear RNA Gene-Targeting Non-LTR Retrotransposons Utopia
Source: PLoS One. 2015 Nov 10;10(11):e0140084. doi: 10.1371/journal.pone.0140084 (PMC4640811; doi:10.1371/journal.pone.0140084)

## S5 Figure

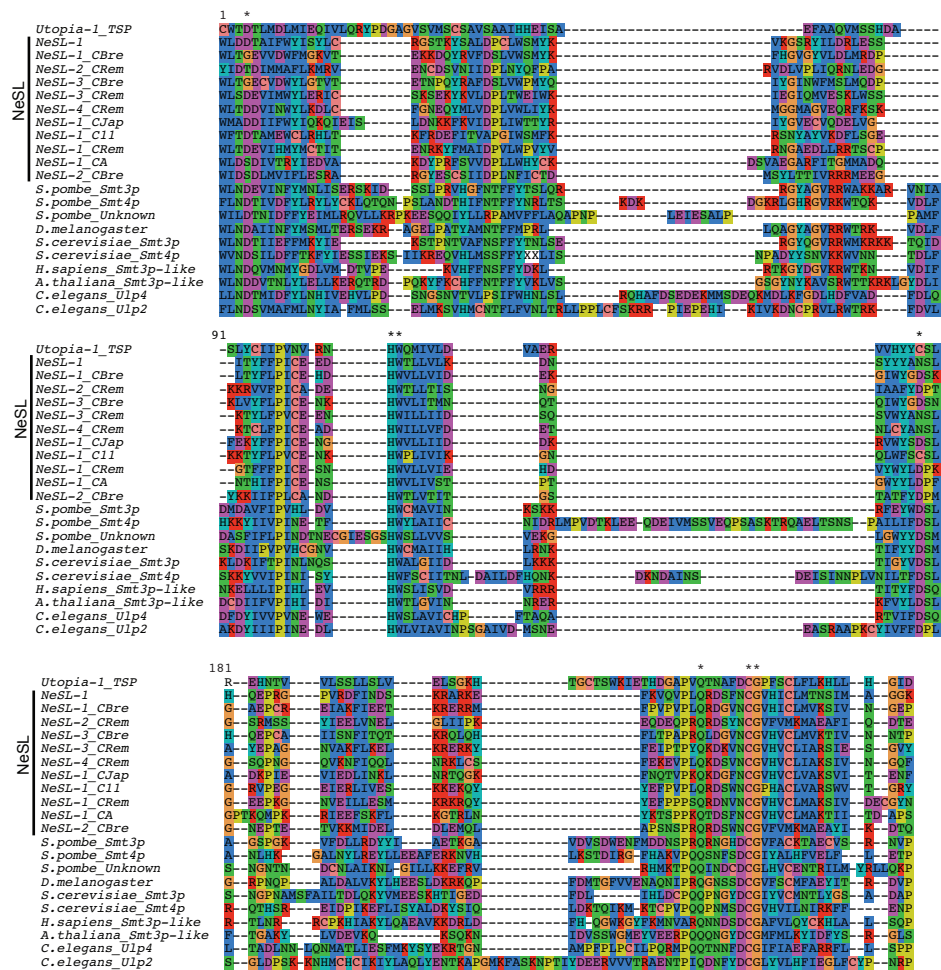

Supplement: S5 Fig — (PDF) [file pone.0140084.s005.pdf]
