# Supplementary material for: Ancient Origin of the U2 Small Nuclear RNA Gene-Targeting Non-LTR Retrotransposons Utopia
Source: PLoS One. 2015 Nov 10;10(11):e0140084. doi: 10.1371/journal.pone.0140084 (PMC4640811; doi:10.1371/journal.pone.0140084)
Supplement: S6 Fig — Retrotransposon sequences are in lowercase while flanking sequences are in uppercase. Sequences similar to target sequences are in red. (PDF) [file pone.0140084.s006.pdf]

S6 Figure.

LIN9_SM

3' boundaries

```
28S rDNA      AAAGCGACGTCGCTATGAACGCTTGGCCGCCACAAGCCAGTTATCCCTGTGGTAACTTTTCTGACACCTCTTGCTACAAACTCTGTAAAAATCAAAAGGATCGATAGGCCGCGCTTTCAC
AAWT01003009  ttcagatgctcacggtgatatgaaaggacaccactagtaaaaaccgctagtttttTTTTCTGACACCTCTTGCTACAAACTCTGTAAAAATCAAAAGGATCGATAGGCCGCGCTTTCAC
AAWT01060376  ttcagatactcacggtgatatgaaaggacaccactagtaacaaccgc-aa-tttttTTTTCTGACACCTCTTGCTACAAACTCTGTAAAAATCAAAAGGATCGATAGGCCGCGCGTTCAC
AAWT01078088  ttcagatgctcgcggtgatatgaatggacaccactagtaaaaaccgctag-tttttTTTTCTGACACCTCTTGCTTCAAACTCTGAAGACATCAAAAGGATCAA
AAWT01077286  ttcagatgctcacggtgatatgaaaggacaccactagtaaaaaccgttag-tttttTTTTCTGACACCTCTTGC
AAWT01088271  ctcagatgctcacggtgatatgaaaagacaacactagtaaaaaccgctag-tttatTTTTCTGACACTTCTTGCTACAAACTCTGTAAAAATCAAAAGGATCGATAGGCCGCGCTTTCAC
AAWT01022977  ttcagatgctcgcggggatatgaaaggataccactagtaaaaaccactag-tttttTTTTCTGACACCTCTTGCTACAAACTCTGTAAAAATCAAAAGGATCGATAGGCCGCGCTTTCAC
AAWT01009947  ctcagatgctcacggtgatatgaaaggacaccactagtaaaaaccgctcg-tttttTTTTCTGACACCTCTTGCTACAAACTCTGTAAAAATCAAAAGGATCGATAGGCCGCGCTTTCAC
AAWT01030948  ttcagacgctcacggtgacaagaaagaacaccactagtaaaaaccactagtag----TTTCTGACACCTCTTGCTTCAAACTCTGAAGACATCAAAAGGATCGATAGGCCGCGCTTTCAC
AAWT01008734  ttcagacactcacggtgacaagaaaggacaccactagtaaaaaccactag------TTTTCTGACACCTCTTGCTACAAACTCTGTAAAAATCAAAAGGATCGATAGGCCGCGCTTTCAC
```

5' boundaries

```
28S rDNA      AAAGCGACGTCGCTATGAACGCTTGGCCGCCACAAGCCAGTTATCCCTGTGGTAACTTTTCTGACACCTCTTGCTACAAACTCTGTAAAAATCAAAAGGATCGATAGGCCGCGCTTTCAC
AAWT01008734  AAAGCGACGTCGCTATGAACGCTTGGCCGCCACAAGCCAGTTATCCCTGCGGTAACattgtggaactcataagacaagtactaaaagaagaattagaaaaattagaagaaaaaattgaaa
AAWT01008734  AAA-CGACAT--C-ATGAACGCTTGGCCGCAACAATCCAGTTATCCCTGCGGTAACattgtggaactcataagacaagtactaaaagaagaattagaaaaattagaagaaaaaattgaaa
AAWT01008741  AAA-CGACAT--C-ATGAACGCTTGGCCACAACAATCCAGTTATCCCTGCGGTAACattgtggaactcataagacaagtactaaaagaagaattagaaaaattagaagaaaaaattgaaa
AAWT01077338  --------------ATGAACGCTTGGCCGCAACAATCTAGTTATCCCTGCGGTAACattgtggaactcaaaagacaagtactaaaagaagaattagaaaaattagaagaaaaaattgaaa
AAWT01044953  AAAGCGACGTCGCTATGAACGCTTGGCCGCCACAAGCCAGTTATCCCTGTGGTAACattgtggaactcataagacaagtactaaaagaagaattagaaaaattagaagaaaaaattgaaa
AAWT01081629  AAA-CGACAT--C-ATGAACGCTTGGCCGCAACAATCCAGTTATCCCTGCGGTAACattgtggaactcataagacaagtactaaaagaagaattagaaaaattagaagaaaaaattgaaa
AAWT01005389  AAAGCGACGTCGCTATGAACGCTTGGCCGCCACAAGCCAGTTATCCCTGCGGTAACattgtggaactcataagacaagtacgaaaagaagaattagaaaaattagaagaaaaaattgaaa
AAWT01058011  AAA-CGACAT--C-ATGAACGCTTGGCCGCAACAATCCAGTTATCCCTGCGGTAACattgtggaactcataagacaagtactaaaagaagaattagaaaaattagaagaaaaaattgaaa
AAWT01008734  AAA-CGACAT--C-ATGAACGCTTGGCCGCAACAATCCAGTTATCCCTGCGGTAACattgtggaactcataagacaagtactaaaagaagaattagaaaaattagaagaaaaaattgaaa
AAWT01008741  AAA-CGACAT--C-ATGAACGCTTGGCCACAACAATCCAGTTATCCCTGCGGTAACattgtggaactcataagacaagtactaaaagaagaattagaaaaattagaagaaaaaattgaaa
AAWT01044928  AAA-CGACA--AC-ATGAACGCTTGGCCGCAACAATCCAGTTATCCCTGCGGTAACattgtggaactcataagacaagtactaaaagaagaattagaaaaattagaagaaaaaattgaaa
AAWT01030948  AAAGCGACGTCGCTATGAATGCTTGGCCGCAACAATCCAGTTATCCCTGCGCTAACattgtggaactcataagacaagtactaaaagaagaattagaaaaattagaagaaaaaattgaaa
```

LIN15_SM

3' boundaries

```
tRNA-Arg-CCG  -TTGACCTGGTGGCCTAATGGATAAGGCGTCGGTTTCCGGAGCCGAAGATTGAGGGTTCGAGTCCCTTCCAGGTCGAATTTTTT--TCTTTGATGAATATATTATTTCGGAATTTTTTCA
AAWT01018554  aatgttataattaatatatttttaaaGCGTCGGTTTCCGGAGCCGAAGATTGAGGGTTCGAGTCCCTTCCAGGTCGAATTTTTT--TCTTTGATGAATATATTATTTCGGAATTTTTTCA
AAWT01076251  aatgttataattaatatatttttaaaGCGTCGGTTTCCGGAGCCGAAGATTGAGGGTTCGAGTCCCTTCCAGGTCGAATTTTTT--TCTTTGATGAATATATTATTTCGGAATTTTTTCA
AAWT01043703  aatgttataattaatatatttttaaaGCGTCGGTTTCCGGAGCCGAAGATTGAGGGTTCGAGTCCCTTCCAGGTCGAATTTTTT--TCTTTGATGAATATATTATTTCGGAATTTTTTCA
AAWT01076251  aatgttataatttatatatttttaaaGCGTCGGTTTCCGGAGCCGAAGATTGAGGGTTCGAGTCCCTTCCAGGTCGAATTTTTT--TCTTTGATGAATATATTATTTCGGAATTTTTTCA

tRNA-Arg-CCT  TAAGCCCGCGTGGCCTAATGGATAAGGCGTCTGCCTCCTAAGCAGAAGATTGCGGGTTCGAGTCCCGCCGTGGGTAAATTTTAT--TCTCAACTAGAAATTTTAAAATTTAGAAAATTGA
AAWT01081073  aatgttataattaatatatttttaaaGCGTCTGCCTCCTAAGCAGAAGATTGCGGGTTCGAGTCCCACCGTGGGTAAATTTTAT--TCTCAACTAGAAATTTTAAAATTTAGAAAATTGA
AAWT01055983  catgttataattaatatatttttaaaGGGTCTTCCTCCTTAGCAGAATATTGCGGGTTCGAGTCCCGCCGTGGGTAAATTTTAT--TCTCAACTAGAAATTTTAAAATTTAGAAAATTGA
AAWT01009870  aatgttataattaatatatttttaaaGCGTCTGCCTCCTAAGCAGAAGATTGCGGGTTCGAGTCCCGCCGTGGGTAAATTTTAT--TCTCAACTAGAAATTTTAAAATTTAGAAAATTGA
AAWT01081074  aatgttataactaatatatttttaaaGCGTCTGCCTCCTAAGCAGAAGATTGCGGGTTCGAGTCCCGCCGTGGGTAAATTTTAT--TCTCAACTAGAAATTTTAAAATTTAGAAAATTGA
AAWT01013479  aatgttataattaatatatttttaaaGCGTCTGCCTCCTAAGCAGAAGATTGCGGGTTCGAGTCCCGCCGTGGGTAAATTTTAT--TCTCAACTAGAAATTTTAAAATTTAGAAAATTGA
AAWT01027286  aatgttataattaatatatttttaaaGCGTCTGCCTCCTAAGCAGAAGATTGCGGGTTCGAGTCCCGCCGTGGGTAAATTTTAT--TCTCAACTAGAAATTTTAAAATTTAGAAAATTGA

tRNA-Arg-TCG  TAAGGCCCTGTGGCCCAATGGATAAGGCGTCTGACTTCGAATCAGAAGATTGCAGGTTCGAGTCCTGCCAGGGTCGTATTTTTTTGTCATTCTTGTTAATTCTAATTTTTAGGCCCTGCA
AAWT01076691  aatgttatgattaatatatttttaaaGCGTCTGACTTCGAATCAGAAGATTGCAGGTTCGAGTCCTGCCAGGGTCGTATTTTTTTGTCATTCTTGTTAATTCTAATTTTTAGGCCCTGCA
AAWT01073663  aatgttataattaatatatttttaaaGCGTCTGACTTCGAATCAGAAGATTGCAGGTTCGAGTCCTGCCAGGGTCGTATTTTTTTGTCATTCTTGTTAATTCTAATTTTTAGGCCCTGCA
AAWT01032776  aatgttataattaatatatttttaaaGCGTCTGACTTCGAATCAGAAGATTGCAGGTTCGAGTCCTGCCAGGGTCGTATTTTTTTGTCATTCTTGTTAATTCTAATTTTTAGGCCCTGCA
AAWT01079374  aatgttataattaatatatttttaaaGCGTCTGACTTCGAATCAGAAGATTGCAGGTTCGAGTCCTGCCAGGGTCGTATTTTTTTGTCATTCTTGTTAATTCTAATTTTTAGGCCCTGCA

tRNA-Arg-TCT  AGAGTCTCAGTGGCGCAATGGATAGCGCGTCGGACTTCTAATCCGGAGGTTGTGGGTTCGAGTCCCACCTGAGATGCATTTTTACAGAATTTTGAGTGAGAAAATTCTGTTTTGTTTTCA
AAWT01086548  aatgttataattaatatatttttaaaGCGTCGGACTTCTAATCCGGAGGTTGTGGGTTCGAGTCCCACCTGAGATGCATTTTTACAGAATTTTGAGTGAGAAAATTCTGTTTTGTTTTCA
AAWT01004690  aatgttataattaatatatttttaaaGCGTCGGACTTCTAATCCGGAGGTTGTGGGTTCGAGTCCCACCTGAGATGCATTTTTACAGAATTTTGAGTGAGAAAATTCTGTTTTGTTTTCA
```

5' boundaries

```
tRNA-Arg-TCG  TAAGGCCCTGTGGCCCAATGGATAAGGCGTCTGACTTCGAATCAGAAGATTGCAGGTTCGAGTCCTGCCAGGGTCGTATTTTTTTGTCATTCTTGTTAATTCTAATTTTTAGGCCCTGCA
AAWT01055054  TAAGGCCCTGTGGCCCAATGGATAAGGCGTCTGACTTCGAATTAGAAGATTGCAGGTTCGAGTCCTGaaacaattacacacataaattctttcctgagcgaaatagataaagtctaccct
```

LIN24_SM

3' boundaries

```
28S rDNA        TAAGAGAGTCATAGTTACTCCCGCCGTTAACCAGAGCTTAGTTGAATTTCTTCACATTGACATTCGAATAGCACTGGGCAGAAATCACGTTGTGTCAAAGCCGTTTACCAGCCATCACAA
AAWT01013143    aagcttgtaaagccgcttgttatattataataaattgtta-----------------tttttttttttttttttttttttttttttACATTGCGTCAAAGCCATTTACTAGCCATCGCAA
AAWT01013144    aagcttgtaaagccgcttgttatattataataaaatggtaatttttttttttttttttttttttttttttttttttttttttttttACATTGCGTCAAAGCCATTTACTAGCCATCGCAA
AAWT01013391    aagcttgtaaagccgcttgttatattataataaattgtta---------tttttttttttttttttttttttttttttttttttttACATTGCGTCAAAGCCATTTACTAGCCATCGCAA
AAWT01022575    aagcttgtaaagccgcttgttatattacaataaattgtta--------------------------ttttttttttttttttttttACATTGCGTCAAAGACATTTACTAGCCATCGCAA
AAWT01046698    aagcttgtaaagccgcttgttatattataataaattgtta-------------------tttttttttttttttttttttttttttACGTTGTGTCAAAGCCGTTTACCAGCCATCACAA
AAWT01006698    aagcttgtaaagccgcttgttatactataataaaatgtta---------------------------------ttattttttttttACATTGCGTCAAAGCCATTTACTAGCCATCGCAA
AAWT01051718    aagcttgtaaagccgcttgtctatattataataaactggt---------------------------------------tttttttACATTGCGTCAAAGCCATTTACTAGCCATCGCAA
AAWT01060466    aagcttgtaaagccgcttgttatattacaataaattgtta--------------------------ttttttttttttttttttttACATTGCGTCAAAGACATTTACTAGCCATCGCAA
AAWT01022969    aagcttgtaaagccgcttgttatattataataaattgtta-----------------tttttttttttttttttttttttttttttACGTTGTGTCAAAGCTGTTTACCAGCCAAGCCGT
AAWT01050849    aagcttgtaaagccgcttgttatattataataaattgtta--------------------ttttttttttttatttttttttttttACGTTGTGTCAAAGCCATTTACCAGAAAAAAAGA
AAWT01071922    aagcttgtaaagccgcttgttatattataataaattgttt---------------------tttttttttttttttgtttttttttACGTTGTGTCAAAGCCGTTTTCCAGtGGACACTC
AAWT01013779    aagcttgtaaagccgcttgttatattataataaattgttt-------------------------------tttttttttttttttACGTTGTGTCAAAGCCGTTTACCAGCCAGTGCGA
AAWT01003456    aagcttgtaaagccgcttgttatattataataaattgtt------------------------tttttttttttttttttttttttACGTTGTGTCAAAGCCGTTTACCAGCCGATTGTA
AAWT01030271    aagcttgtaaagccgcttgttatactataataaaatgtta------------------------------ttatttttttttttttACATTGCG
AAWT01025957    aagcttgtaaagccgcttgttatattataataaattgtta---------------tttttttttttttttttttttttttttttttAGATTCTCTGATGCCCAAGTGCTAATTGATTTTA
AAWT01050383    aagcttgtaaagccgcttgttatattataataaattgtta------------------------ttttttttttttttttttttttAGATTCTCTGATGCTCAAGTGCTAATTGATTTTA
AAWT01079086    aagcttgtaaagccgcttgttatattacaataaattgtta-------------------------------tttttttttgtttttCTGCTCTGATGTTGAAATTTTGACATCTTTTTTA
```

LIN25_SM

3' boundaries

```
tRNA-Glu-TTC    gaatgaatatttggtaataatattataattaactaaTCCCTGATGGTCTAGCGGTTAGGATTCCTGGTTTTCACCCAGGCGGCCCGGGTTCGACTCCCGGTCAGGGAA
AAWT01038405    gtaaaaattgacaaatttattataaataataaacgtttaaTGATGGTCTAGCGGTTAGGATTCCTGGTTTTCACCCAGGCGGCCCGGGTTCGACTCCCGGTCAGGGAA
```

```
AAWT01050628    gtaaaaattgacaaatttattataaataataaacgtttaaTGATGGTCTAGCGGTTAGGATTCCTGGTTTTCACCCAGGCGGCCCGGGTTCGACTCCCGGTCAGGGAA
AAWT01053061    gtaaaaattgacaaatttattataaataataaacgtttaaTGATGGTCTAGCGGTTAGGATTCCTGGTTTTCACCCAGGCGGCCCGGGTTCGACTCCCGGTCAGGGAA
AAWT01053509    gtaaaaattgacaaatttattataaataataaacgtttaaTGATGGTCTAGCGGTTAGGATTCCTGGTTTTCACCCAGGCGGCCCGGGTTCGACTCCCGGTCAGGGAA
AAWT01054914    gtaaaaattgacaaatttattataaataataaacgtttaaTGATGGTCTAGCGGTTAGGATTCCTGGTTTTCACCCAGGCGGCCCGGGTTCGACTCCCGGTCAGGGAA
AAWT01028898    gtaaaaattgacaaatttattataaataataaacgtttaaTGATGGTCTAGCGGTTAGGATTCCTGGCTCTCACCCAGGTGGCCCGGGTTCGACTCCCGGTCAGGGAA
AAWT01043390    gtaaaaattgacaaatttattataaataataaacgtttaaTGATGGTCTAGCGGTTAGGATTCCTGGCTCTCACCCAGGTGGCCCGGGTTCGACTACCGGTCAGGGAA
AAWT01053082    gtaaaaattgacaaatttattataaataataaacgtttaaTGATGGTCTAGCGGTTAGGATTCCTGGCTCTCACCCAGGTGGCCCGGGTTCGACTCCCGGTCAGGGAA
AAWT01007554    gtattaaatgtcaaacttattataaataataaatttttacTGATGGTCTAGCGGTTAGGATTCTTGGCTCTCACCCAGGTGGCCCGGGTTCGACTCCCGGTCAGGGAA
AAWT01010426    gtaaaaattgtcaaacttattataaataataaacttttatTGATAGCTCAGTTGGTAGAGCTGAGGACTGTAGTGGTAGCAGTGATCCTTAGGTCGGTGGTTCAAATC

Polinton-2_SM   ACTCATACGTTTATATGAAGTAAAAAGTATAATAATAATACCAATAGTACTAGTGAGTGATTTTGCTAAGTCTACGACTTAGTCGTAGACGTAGTTGTAGGCGTGTTG
SINEL_SM        ACTCTTACGTTTATATGAAGTAAACAGTATAATAATAATACCAATAGTACTAGTGAGTGATTTTGTTAAGTCTACGACTTAGTCGTAGACGTAGTTGTAGGCGTGTTG
AAWT01045199    gtattaaatgtcaaacttattataaataataaattttttaaTGAATAGTACTAGTGAGTGATTTTGTTA------CGACTTATTCGTAGACGTAGTTGTAGGCGTATTG
AAWT01066673    gtattaaatgtcaaacttattataaataataaattttttaaTGAATAGTACTAGTGAATGATTTTGTTA------CGACTTAGTCGTAGACGTAGTTGTAGGCGTATTG

Polinton-4_SM   AAAAATTTAGAGTGAAAAAAAATGATAAAAAAATCAACCCGGATAGCTCAGTGGGAATAGAGTAGCACAGTGGTTCAAATAGACATGGGTTCAAATCCCAACTAGAAT
AAWT01034431    gtaaaatatgtcaaacttattataaataataaattttttaaAGATAGCTCAGTGGGAATAGAGTTGCACTGTGGTTCAAATAGACAAGG-TTCATATTCCAACTAGAAT
AAWT01055093    gtaaaatatgtcaaacttattatgaataataaattttttaaAGATAGCTCAGTGGGAATAGAGTTGCACTGTGGTTCAAATAGATAAAG-TTCATATTCCAACTAGAAT
AAWT01036164    gcaaaaaatgtcaaacttattataaataattaactttttaaCGATAACTCAGTCCAAATAGTAGTTCATAGTGATTTAAATAGATATGGGTTCAAATCCCAACTAGAAT
AAWT01040864    gtaaaaattgtcaaccttattataaataataaacttttatTCATGGTACAGTAGGAA--GCTATTCACATTTTTCATCATAGCGCCGGGTTCAAATCCTGATTTGGAC
AAWT01080863    gtaaaaattgtcaaacttattataaatagtaaatttttaaAGATGGTACAGTGTG-GAATACATTAAACTTTGCATCCGGGCGTCGTGATTCAAATCCTAATTTCAGT

AAWT01073331    gtaaaaattgtcaaacttattataaataataaattttttaaTGATTTTATTTGTTAATAATCATTTGCATAGAAGTTATACTGTCATTCGATTTATGAATGGAAATTGA
AAWT01080446    gtaaaaattcacaaacttat--ataataaaaaacgtttatTGATTTTATAGTATACCAGAAACGATAATTATCGCGATACAAATAATTTTTTCCATTATTGAGTTTGA
AAWT01084288    gtaaaaattgtcaaacttattataaataataaatttttttaATGAATCGGTGATGATCGGTGATCGTTGGTTGATGTGTGTGTGAACGGTTTAAGAACGGTAGGTCCTA
```

LIN26_SM

3’ boundaries

```
28S rDNA      TAAGAGAGTCATAGTTACTCCCGCCGTTAACCAGAGCTTAGTTGAATTTCTTCACATTGACATTCGAATAGCACTGGGCAGAAATCACGTTGTGTCAAAGCCGTTTACCAGCCATCACAA
AAWT01011543  gccacgctcgcatatagtagccgaaaggtcgctttttgttgttaaaataaaatttagt---------------tttttttttttttACGCCGTGTCAAAGCCGTTTACCAGCCATCACAA
AAWT01066798  gccacgctcgcatacagtagccgaaaggtcgctttttgttgttaaaataaaatttagt--------------ttttttttttttttACGTCGTGTCAAAGCCGTTTACCAGCCATCACAA
AAWT01069610  gccacgctcgcatatagtagccgaaagatcgctttttgttgttaaaataaaatttagt-----------tttttttttttttttttACGTCGTGTCAAAGCCGTTTACCAGCCATCACAA
AAWT01079467  gccacgctcgcatatagtagccgaaaggtcgctttttgttgttaaaataaaatttagt-------------tttttttttttttttACGTCGTGTCAAAGCCGTTTACCAGCCATCACAA
AAWT01079467  gccacgctcgcatatagtagccgaaaggtcgctttttgttgttaaaataaaatttagt------------ttttttttttttttttACGTCGTGTCAAAGCCGTTTACCAGCCATCACAA
AAWT01083274  gccacgctcgcatatagtagccgaaagatcgctttttgttgttaaaataaaatttagt----------------ttttttttttttACGTCGTGTCAAAGCCGTTTACCAGCCATCACAA
AAWT01023898  tccacgctcacatatattagccgaaaggccactttttgttgttaaaataaaatttagt-------------ttttttttttttttttaGTCGTGTCAAAGCC-----------ATCACAA
AAWT01003008  gccacgctcgcatatattagccgaaaggccgcttt-gtcatt-aaaataaaatttagt-------tttttttttttttttttttttACGTCGTGTCAAAGCCGTTTACCAGCCATCACAA

Polinton-5_SM CAAATAAAAGTTAACTAACTTGGTATCAACATCTGATTACCGATAACCTCTTCACACCGAATAACCCCCTGATTAAGCGCTATATACTAATAACTAGGCACTATACACCATGTACTGTA
AAWT01000709  gccacgctcgcatatattagccgaaaggccgcttttgttatt-aaaataaaatttagt-------tttttttttttttttttttttAATAGGTACTATACACCATATACCAAGAACTGTA
AAWT01016893  gccacgctcgcatatattagccgaaaggccgcttttgttatt-aaaataaaatttagt-------------tttttttttttttttAATAGGTACTATACACCATATACCAAGAACTGTA
AAWT01067833  gccacgctcgcatatattagccgaaaggccgcttttgttatt-aaaataaaatttagt----------ttttttttttttttttttAATAGGTACTATACACCATATACCAAGAACTGTA

GLT4_SM       TCTTCGCCCATCTCAATTGGCATATCGAGCTCCAACCACCAATCCAACACCAGTCGCGACTCCATCAACGACACCCTCCAGCAGTTACAGGCCGGGGCGCAAGGAACGCGCCAGACGCAG
AAWT01026667  gccacgctcgcatatattagccgaaaggccgcttttgttatt-aaaataaaatttagt--------------------ttttttttACCGGCCGGGGCGTAAGGAACGCGCCAGACGCAG
AAWT01061215  gccacgctcgcatatattagccgaaaggccgcttttgttatt-aaaataaaatttaga------------------ttttttttttACAGGCCGGGGCGCAAGGAGCGCGCCAGACGTAG

piggyBac-9_SM ACAGTGCACCGTTTTCGATCAATACACATGTAGCAGTCACCACGCTTACATGGAGTCGACGATAGAGAGTCGGCGGCAGCAGTCCTTCTTCTGTCTTCTACATACGCACTTATTAAACTG
AAWT01007743  gccacgctcgcatatgttagccgaaaggccgcttttg--atttaaaataaaatttagt----------ttttttttttttttttttCTTCCTGTCTTCTACATACGCACTTATTAAACTG
AAWT01013533  gccacgctcgcatatgttagccgaaaggccgcttttg--atttaaaataaaatttagt----------ttttttttttttttttttCTTCCTGTCTTCTACATACGCACTTATTAAACTG

AAWT01010151  gccacgctcgcatatattagtcgaaaggccgcttttgttatt-aaaataaaatttagt--------------ttttttttttttttAAA--TAAAATAAAA-CATATTAAATAAAACAAA
AAWT01039054  gccacgctcgcatatattagtcgaaaggccgcttttgttatt-aaaataaaatttagt--------ttttttttttttttttttttAAA--TAAAATAAAA-CATATTAAATAAAACAAA
AAWT01073024  gccacgctcgcatatgttagccgaaaggccgcttttg--atttaaaataaaatttagt--------ttttttttttttttttttttAAAAATATAATAAAAACA--TTAAATAAAACAAA
AAWT01004811  gccacgctcgcatatattagccgaaaggccgctttttgtt---aaaataaaatttagt---------tttttttttttttttttttAGATTTTCTGATGCCCAAGTGCTTGTAAATTTTA
AAWT01005272  gccacgctcgcatatattagccgaaaggccgcttttgtcatttaaaataaaatttagt------ttttttttttttttttttttttAGATATTCTGATGACTAGATGCTTGTGAACTTTA
AAWT01064691  gccacgcttgcatatattagccgaaaggccgcttttg--ttttaaaataaaatttagt-------------------tttttttttGATATGAAAAAAATATCAACCCTATTAAATATTA
AAWT01004482  gccacgctcgcatatattagccgaaaggccgttttttg-at--aaaataaaatttagt--------------------ttttttttCAATATGCCAGCAAAATATACCCATCATCACGAC
AAWT01066539  gcctcgctcgcatata--agccgaaaggccgcttttgttat--aaaataaaatttttt--------------------------ttCTACTATTGAGGTTTTATCTATTCAACACAAAT
AAWT01008582  gccacgctcgcatatattagccgaaagtccgcttttgttatt-aaaataaaatttagaaaaaaaaaaaaacaa---------------TTTGTAATAGCTGGGCGATATTTTGTATGAAT
AAWT01020120  gccacgctcgcatata--agccgaaaggccgcttttgttat--aaaataaaattttggaaaaaaaaaaaaaaa---------------CATACTGATGCCACGAGTATAAATAGTCAATG
AAWT01020210  acctagctcgcatatt--agccgaaaggccgcttttgttat--aaaataaaattttgaaaaaaaaaaaaagaaaaaaagaa-------TTGGGAATGATCTTGGAAAGGGTTCATAAAAA
AAWT01025460  gcctagctcgcatata--agccgaaaggccgcttttgttat--aaaataaagttttgaaaaaaaaaaaaaaaaaaaa-----------CTTAGCAAGTAATTAAAATTTTTTATTAAATT
AAWT01032826  gcatcgctcgcatatt-tagccgaaaggtcgttttttgtt---gaaataaaattttgaaaaaaaaaaataaataaaaaaaaaaaaaaaTCTCCCAAGACGATCAGAGTCTTTATTATTCT
AAWT01047706  gcctagctcgcatatt--agccgaaaggccgcttttgttat--aaaataaaattttggaaaaaaaaaaaaaaaagaa-----------TTGGGAATGATCTTGGAAAGGGTTCATAAAAA
AAWT01048908  gccacgctcgcatata--agccgaaaggccgcttttgttat--aaaataaaattttgaaaaaaaaaaaaaaaaaa-------------CCAGCCGGGACGCAAGGAACGTGCCCGACGCA
```

NeSL-1_TV

3’ boundaries

```
tRNA-Pro-TGG    GGGTGAGTAGTCTAGTGGTATGATTCCTGTTTTGGGTACAGGAGGTCCCGGGTTCAATTCCCGGCTCGCCCCATTTTTTCTTTTTTCATT
DS113626        actttgctcgctcgcttcgctcgctcgtcttaacccttccgaataaacacttaCAATTCCCGGCTCGCCCCATTTTTT
DS114997        actttgctcgctcgcttcgctcgctcgtcttaacccttccgaataaacacttaCAATTCCCGGCTCGCCCCATTTTTT
DS113485        actttgctcgctcgcttcgctcgctcgtcttaacccttccgaataaacacttaCAATTCCCGGCTCGCCCCATTTTTT
DS115424        actttgc---------tcgctcgctcgtcttaacccttccgaataaacacttaCAATTCCCGGCTCGCCCCATTACTA
DS133744        actttgctcgctcgcttcgctcgctcgtcttaacccttccgaataaacacttaCAATTCCCGGCTCGCCCCATTTTTT
```

5’ boundaries

```
tRNA-Pro-TGG    GGGTGAGTAGTCTAGTGGTATGATTCCTGTTTTGGGTACAGGAGGTCCCGGGTTCAATTCCCGGCTCGCCCCATTTTTTCTTTTTTCATT
DS113626        GGGTGAGTAGTCTAGTGGTATGATTCCTGTTTTGGGTACAGGAGGTCCCGagaagcttccactgcaatcgtacgtgtactgtggcaacacagctataacagacagtttcacgccaaccgc
DS113298        GGGTGAGTAGTCTAGTGGTATGATTCCTGTTTTGGGTACAGGAGGTCCCGagaagcttccactgcaatcgtacgtgtactgtggcaacacagctataacagacagtttcacgccaaccgc
DS113485        GGGTGAGTAGTCTAGTGGTATGATTCCTGTTTTGGGTACAGGAGGTCCCGagaagcttccactgcaatcgtacgtgtactgtggcaacacagctataacagacagtttcacgccaaccgc
DS113187        GGGTGAGGAGTCTAGTGGTATGATTCCTGTTTTGGGTACAGGAGGTCCCGagaagtttccactgcaatcgtacgtgtactgtggcaacacagctataacagacagtttcacgccaaccgc
DS113586        GGGTGAGTAGTCTAGTGGTATGATTCCTGTTTTGGGTACAGGAGGTCCCGagaagcttccactgcaatcgtacgtgtactgtggcaacacagctataacagacagtttcacgccaaccgc
DS129582        GGGTGAGTAGTCTAGTGGTATGATTCCTGTTTTGGGTACAGGAGGTCCCGagaagcttccactgcaatcgtacgtgtactgtggcaacacagctataacagacagtttcacgccaaccgc
```

Togen-1_DR

3’ boundaries

```
Zv7_NA1020_[50544-51093]             gattatttggatgctgtgttgttctaat-agtattagtcattccaaataaaatgaatcgtgTTCGTGTGTGTGTGTGCGTGTGTGCGTGTGTGCGTGTGTGTGTGTGTGTGTTAAGCCT
Zv7_NA21_[139424-138755]             gattatttggatgctgtgttgttttaat-agtattagtcattccaaataaaatgaatcaaaTGTGTGTGTGTGTGTGTGTGTGTGTGTGTGTGTGTGTGTGTGTGTGTGTGTGTGTGTG
Zv7_scaffold2628_[61683-62495]       gattatttggatgctgtgttgttttaat-agtattagtcattccaaataaaatgaatcaaaTGTGTGTGTGTGTGTGTGTGTGTGTGTGTGTGTGTGTGTGTGTGTGTGGAGGGATTTA
chr11_[17336952-17336478]            gattatttggatgctgtgttgttttaat-agtattagtcattccaaataaaatgaatcaaaTGTGTGTGTGTGTGTGTGTGTGTGTGTGTGTGTGTGTGTGTGTGTGTGTGTGTGTGTG
chr11_[37646849-37647135]            gattatttggatgctgcgttgcgttaattagtattagtcattccaaataaaatgaatcaaaTGTGCATGTGTGTGTGTGTGTGAGAATGTAAGAGTATATAGGTGTTTCCCAGTACTGG
chr12_[32869914-32869431]            gattatttggatgctgtgttgttctaat-agtattagtcattccaaataaaatgaatcaaaTGTTGTGTGTGTGTGTGTGTGTGTGTGTGTGTGTGTGTGTGTGTGTGTGTGTGTGTGT
chr13_[11700615-11699786]            aattatttggatgct---------------------------ccaaataaaatgaatcaaaTGTGTGTGTATGGCTATGTGTGTGCGTTTGTGTGTAAGTGTGTGTGGCTCTTTGTGTG
chr14_[28318614-28322031]            gattatttggatgctgaattgttttaat-agtattagtcattccaaataaaattaatcaaaTGTGTTTGTGTGTGTGTTTGTGTGTGTGTGTGTGTTTCTCAGTCTTCATCAGTGTCAT
chr14_[32752097-32755510]            gattatttggatgctgtgttgttttaat-agtattagtcattccaaataaaatgaatcaaaTGTGTGTGTGACTACTATTACAAACAGCAATGTCTATATATATATATAGAATTTGAAT
chr14_[6426330-6426365]              gaatatttggatgctgtgttgttctaat-agtattagtcattccaaataaaatgaatcaaaTGTGTTTGTGTATATATTGATTCATATTCTTTTCGGCTTAGTCCCTTATTAATCTGGG
chr15_[44782787-44783167]            gattatttggatgctgtgttgctttaattagcaatagtcattccaaataaaatgaatcaaGTGTGTGTGTGTGTGTGTGTGTGTGTGTGTGTGTGTGTTAGGGATGCAACAATACAGTT
```

```
chr16_[14874220-14877639]          gattatttggatgctgtgttgttttaat-agtattagtcattccaaataaaatgaatcaaaTGTGTGTGTGTGTGTGTGTGTGTGTGTGTGTGAATGCGTGTGTGGATGTTTCCCAG
chr16_[18111641-18114787]          gattatttggatgctgtgttgttttaat-agtattagtcattccaaataaaatgaatcaaaTGTGTGTGTGTGTGTGTGTGTGTGTGTGTGTGTGTGTGTGTGTGTGTGTGTGTACA
chr17_[12754170-12753884]          gattatttggatgctgcgttgcgttaattagtattagtcattccaaataaaatgaatcaaaTGTGCATGTGTGTGTGTGTGTGAGAATGTAAGAGTATATAGGTGTTTCCCAGTACTGG
chr17_[14203270-14202976]          gattatttggatgctgtgttgttttaat-agtattagtcattccaaataaaatgaatcaaaTGTGTTGACTCTGAAAATCAGGGCGTGCCCCAATCAACACTCCCATATTTATGCAAAA
chr18_[563432-563651]              gattatttggatgctgtgttgttttaat-agtattagtcattccaaatcaaatgaatcaaaTGTATGTATATATATATATATATATATATATATATATGTATATATATATATATATA
chr19_[10348046-10349748]          aattatttggatgctgtgttattctaat-agtattagtcatttcaaataaaatgaattaaaTGTGTGTTCGTGTGTGTGTGTGTGTGTATAATAACAATAAAAAAGAAAATCTGTCACA
chr2_[30234513-30234751]           gattatttggatgctgtgttgttttaat-agtattagtcattccaaataaaatgaatcaaaTGTGTGTGTGTGTGTGTGTGTGTGTGTGTGTGTGTCTGTGTGTGTGTGTCTGTGTGTG
chr20_[26127395-26129919]          gattatttggatgctgtgttgttttaat-agtattagtcattccaaataaaatgaatcaaaTGTGTGTGTATTCAAAATTTTGATCTCCAAATATACAGCTTAATGCACAAATGTTTTA
chr20_[43148983-43147753]          gattatttggatgctgtgttgttttaat-agtattagtcattccaaataaaatgaatcaaaTGTGTGTGTGTGTGTGTGTGTGTGTGTGTGTGTGTGTGTGTGGAAAATACTCATAGCG
chr20_[47783112-47783911]          gattatttggatgctgtgttgttttaat-agtattagtcattccaaataaaatgaatcaaaTGTGTGTGTGTGTGTGTGTGTGTGTGTATACACACACACACACACACACACACACACA
chr20_[47970334-47970590]          gattatttggatgctgtgttgttttaat-agtattagtcattccaaataaaatgaatcaaaTGTGTGTGTGTGTGTGTGTGTGTGTGTGTGTGTCGTAGCAATGGAGCCGCTGGTTCGCTG
chr20_[7210638-7210074]            ggttatatggatgctgtgttgctttaattagtattagtcattccaaataaaattaatcaaaTGTGTGTGGATGTTTCCCAGAGATGGGTTGTGGCTGGAAGGGCATCTGCTGCGTAAAA
chr21_[41165320-41165079]          gattatttggatgctgtgttgcttttattagtattagtcattctaaataaaatgaatcaaaTGTGTGTGTGTGTGTGTGTGTGTGTGTGTGTGTGTGTGTGTGTGTGTGTGTGTGTGTG
chr22_[24295736-24295160]          gattatttggatgctgtgttgttctaat-agtattagtcattccaaataaaatgaatcaaaTTTGTGTGTGTGTAACCACAAGCTTTCGAGATGTGCATTAAAAAGTTAGCACCGAATT
chr24_[34903493-34904016]          gattatttggatgctgtgttgttttaat-agtattagtcattccaaataaaatgaatcaagTGTGTGTGTGTGTGTGTGTGTGTGTGTGTATTTATTTATATATTTTTTTCTGTTTAAAGG
chr24_[39351951-39352483]          gattatttggatgctgtgttgttctaat-agtattagtcattccaaataaaatgaatcaaaTGTGAGTAAGTCTTTTAGCCCGTTTCCACTGAGTGGTGCGATATTGTGTGGTTCGGTA
chr3_[881150-881350]               gattatttggatgctgtgttgttttaat-agtattagtcattccaaataaaatgaatcaaaTGTGTGTGTGTGTATGTATGTATGTATGTATGTATGTGTGTATGTGTGTGTGTGTGTG
chr4_[31692477-31692425]           gattatttggatgctgtgttgttttaat-agtattagtcattccaaataaaatgaatcaaaTGTGTGTGAGAAAGCATGTTTGTGTTTATGAGAGAGCATGTGTGTGTGAGTGAGAGAA
chr4_[39729528-39728635]           gattatttggatgctgtgttgttttaat-agtgttagtcattccaaataaaatgaatcaaaTGTGTGTGTGTGTTCAGGTAACCCGCAGTGTGATGAAGGCGTCTCTGGCCCTCCCAGC
chr6_[49457277-49457581]           gattatttggatgctgtgttgttttaat-agtattagtcattccaaataaaatgaatcaaaTGTGTGTGTGTGTATGTGTGAATGAGTGTGAATAATGGTCCGTTTCCAGTGAGAGGTA
chr7_[24116604-24117775]           gattatttggatgctgtgttgttttaat-agtattagtcattccaaataaaataaatcaaaTGTGTGTGTGTGTGTGTGTGTGTGTGTGTGTGTGTGTGTGTGTGTGTGTGTGTGTGTG
chr7_[53451622-53451872]           gattatttggttgctgtgttgttctaat-agtattagtcactccaaataaaatgaatcaaaTGTGTGTGTGTGTGTGTGTGTGTGTGTATGTGTGTGTGTGTGTGTGTGTGTGTCCTGC
chr8_[5633558-5631681]             gattatttggatgctgtgttgttttaat-agtattagtcattccaaataaaatgaatcaaaTGTGTGTGTGTGTAAGTCTGTAGAGTTCAACAGGTGTCCTTGACAGGGAACAGGAATT
chr9_[3846398-3846160]             gattatttggatgctgtgttgctttaattagtgttagtcattccaaataaaatgaatcaaaTGTGTGTGTGTGTGTGTGTGTGTGTGTGTGTGTGTGTGTGTGTGTGTGTGTGTGTGTG
chr9_[42228309-42228545]           gattatttggatgctgtgttgttctaat-agtattagtcattccaaataaaatgaatcaaaTGTGTGTGTGTGTGTGCGTGTATGTATATATATATATATATATATATATATATATATA
Zv7_NA4518_[7380-7132]             gattatttggatgctgtgttgctctaat-agtattagtcattccaaataaaatgaatcaagTGTGTGTGTGTGTGTGTGTGTGTGTGTGTGTGTGTGTGTGTGTTTGCATATACAGTTG
chr18_[41903299-41902919]          gattatttgaatgctgtgttgctttaattagtattagtcattccaaataaaattaatcaaaTGTGTGTGTGTGTGTGTGTGTGTGTGTGTGTGTGTGTGTGTGAATTAAAAAAAAAACAT
```

5’ boundaries

```
chr14_[32752097-32755510]          GCTCAAAGACAAACACTAGTGCTGTTACTGTGTGTGTGTGTGTGTGTGTATGTGTGTGGATGcgcatgcgttgggagtaacatggcgattgctcccacgcgcaaacatgctgcactaaacaa
chr17_[2359635-2357363]            TGTATGTCTGTTTGTGTGTGTTTGTTTCTTTCTGTGTGTGTTATACAGTTTGTGTGTGTGcgcatgcgttgggagtaacatggcgattgctcccacgcgcaaacatgctgcactaaacaa
chr18_[48922789-48924360]          TGTGTGTGTGTGTGTGTGTGTGTGTGTGTGTGTGTGTGTGTGTGTGTGTGTGTGTGTGTGcgcatgcgttgggagtaacatggcgattgctcccacgcgcaaacatgctgcactaaacaa
chr20_[43152697-43150437]          TATAAACTGCAGGTTAACTGTGTGTGTGTGTGTGTGTGTGTGTGTGTGTGTGCGTGTGTGcgcatgcgttgggagtaacatggcgattgctcccacgcgcaaacatgctgcactaaacaa
chr7_[24109574-24110015]           TCCTCCGAGGAGGAGGGTAATGTGTGTGTGTGTGTGTGTGTGTGTGTGTGTGTGTGTGTGcgcatgcgttgggagtaacatggcgattgctcccacgcgcaaacatgctgcactaagcaa
```
