# Supplementary material for: Ancient Origin of the U2 Small Nuclear RNA Gene-Targeting Non-LTR Retrotransposons Utopia
Source: PLoS One. 2015 Nov 10;10(11):e0140084. doi: 10.1371/journal.pone.0140084 (PMC4640811; doi:10.1371/journal.pone.0140084)
Supplement: S1 Table — (PDF) [file pone.0140084.s007.pdf]

S1 Table. *Utopia* transposons used in this study.

Classification	Organism	Element name[1]	Representative sequence accession number or reference
Acanthamoeba	*Acanthamoeba castellanii*	*Utopia-1_ACa (NeSL-1_ACa*)*	Kapitonov and Jurka 2009b
Oomycetes	*Phytophthora infestans*	*Utopia-1_PI (R2I-1_PI*)*	Kapitonov and Jurka 2009a
		Utopia-2_PI (R2I-2_PI)*	Kapitonov and Jurka 2009a
		Utopia-3_PI (R2I-3_PI)*	Kapitonov and Jurka 2009a
		Utopia-4_PI (R2I-4_PI)*	Kapitonov and Jurka 2009a
		*Utopia-5_PI**	AATU01010946 5826-458
	Phytophthora sojae	*Utopia-1_PS**	AATU02000290 151602-147317
		*Utopia-2_PS**	AAQY02000317 56568-61511
	Phytophthora ramorum	*Utopia-1_PR**	AAQX01002784 5895-373
		*Utopia-2_PR**	AAQX01002782 11665-6560
		*Utopia-3_PR**	AAQX01002786 4190-1
		*Utopia-4_PR**	AAQX01000364 1-4164
		*Utopia-5_PR**	AAQX01001557 4943-1076
	Phytophthora capsici	*Utopia-1_PCa**	ADVJ01006931 1-5715
		*Utopia-2_PCa**	ADVJ01005511 6099-711
		*Utopia-3_PCa**	ADVJ01006926 1-3090
	Pythium ultimum	*Utopia-1_PU**	ADOS01001321 16954-22061
		Utopia-2_PU	ADOS01001527 6306-13211
	Saprolegnia parasitica	*Utopia-1_SaPa**	ADCG01001153 2696-1
	Saprolegnia diclina	*Utopia-1_SaDi*	AIJL01003665 1-5131

	Hyaloperonospora arabidopsidis	*Utopia-1_HAra*	ABWE02004867 1548-1, ABWE02006758 1584-1
	Pseudoperonospora cubensis	*Utopia-1_PCu*	AHJF01004292 3113-1
Arthropoda	*Nasonia vitripennis*	*Utopia-1_NVit**	AAZX01023258 6989-10984
	Ganaspis sp. G1	*Utopia-1_GG1**	GAIW01019287
	Lasioglossum albipes	*Utopia-1_LAl*	ANOB01038936 1-901
	Megachile rotundata	*Utopia-1_MRo**	AFJA01012833 1616-1
	Solenopsis invicta	*Utopia-1_SIn**	AEAQ01004818 1-3581
	Acromyrmex echinatior	*Utopia-1_AEc**	AEVX01007346 14080-9651
	Atta cephalotes	*Utopia-1_ACep*	ADTU01020077 6267-1
	Pogonomyrmex barbatus	*Utopia-1_PBa**	ADIH01019771 13896-9728
	Harpegnathos saltator	*Utopia-1_HSal*	AEAC01025536 5360-1764
	Camponotus floridanus	*Utopia-1_CFl*	AEAB01024608 6010-206
	Drosophila yakuba	*Utopia-1_DYak**	AAEU02000228 170743-167731
	Heliconius melpomene	*Utopia-1_HMM**	CAEZ01003146 10281-6394
	Chrysopa pallens	*Utopia-1_CPa*	GAGF01007276 1-3418
	Dendroctonus ponderosae	*Utopia-1_DPo**	APGL01014278 340-2348
	Agrilus planipennis	*Utopia-1_APl*	GAAB010068679, GAAB01011383
	Acyrthosiphon pisum	*Utopia-1_APi**	ABLF02060616 1-2997
		*Utopia-2_APi**	ABLF02021456 14527-11959
	Ladona fulva	*Utopia-1_LFu*	APVN01008518 6893-4277
		Utopia-2_LFu	APVN01011335 7139-5136
		Utopia-3_LFu	APVN01022670 4324-7137
		Utopia-4_LFu	APVN01023172 17077-14325
		Utopia-5_LFu	APVN01034835 4467-6794

		Utopia-6_LFu	APVN01044638 3468-428
	Daphnia pulex	*Utopia-1_DPu*	ACJG01000357 58205-53595
		Utopia-2_DPu	ACJG01003145 6300-2161
		Utopia-3_DPu	ACJG01010545 2867-5887
	Strigamia maritima	*Utopia-1_SM**	AFFK01010958 426-4816
	Ixodes scapularis	*Utopia-1_IS**	ABJB010418708 825-3421
	Parasteatoda tepidariorum	*Utopia-1_PT**	AOMJ01245954 24152-17421
Nematoda	*Trichinella spiralis*	*Utopia-1_TSP**	ABIR02001268 5958-475
	Pristionchus pacificus	*Utopia-1_PPac**	ABKE01006509 1-4196
Chordata	*Ciona savignyi*	*YURE-2_Cis (R2-1a_Cis)*	Smit 2008
		YURE_CSa	Kojima and Jurka 2012
	Ciona intestinalis	*YURECi*	Kojima and Fujiwara 2004
	Chrysemys picta	*Utopia-1_CPB**	AHGY01240971 2830-7805
		*Utopia-1B_CPB**	AHGY01381757 6888-12068
		*Utopia-2_CPB**	AHGY01246147 4688-423
		*Utopia-3_CPB**	AHGY01048556 31964-27426
	Chelonia mydas	*Utopia-1_CMy**	AJIM01159899 8360-4147
		*Utopia-2_CMy**	AJIM01175835 13299-8408
	Pelodiscus sinensis	*Utopia-1_PSi**	AGCU01127001 1203-5587
	Anolis carolinensis	*Utopia-1_ACar (NeSL-1_ACar)*	Kojima and Jurka 2012
	Alligator mississippiensis	*Utopia-1_AMi**	AKHW01026695 1828-6544
Echinodermata	*Strongylocentrotus purpuratus*	*Utopia-1_SP**	AAGJ04138299 6919-1360
	Lytechinus variegatus	*Utopia-1_LV**	AGCV01358106 4498-469
	Patiria miniata	*Utopia-1_PMi**	AKZP01013404 8903-5221

1 For families whose names are replaced in this study, original names are shown in

parentheses.

* At least one copy are flanked by fragments of U2 snRNA genes.
