# Supplementary material for: Ancient Origin of the U2 Small Nuclear RNA Gene-Targeting Non-LTR Retrotransposons Utopia
Source: PLoS One. 2015 Nov 10;10(11):e0140084. doi: 10.1371/journal.pone.0140084 (PMC4640811; doi:10.1371/journal.pone.0140084)
Supplement: S2 Table — (PDF) [file pone.0140084.s008.pdf]

S2 Table. All RT-coding sequences of *Utopia* elements in *P. infestans*.

Family	Accession no.	Position of RT	Identity[1]	3' Flanking seq.
Utopia-1_PI	AATU01001290	17964-16681	100%	U2 (15271-15207)
	AATU01001290	4873-3590	99%	U2 (2178-2114)
	AATU01001290	11403-10120	99%	U2 (8718-8654)
	AATU01001283	25796-27079	99%	U2 (28487-28551)
	AATU01001281	22100-23383	99%	U2 (24789-24891)
	AATU01001281	2031-3315	97%	U2 (4723-4787)
	AATU01000136	531-1813	99%	Unsequenced
	AATU01000159	54->1046	98%	Unsequenced
	AATU01001296	962-<3	98%	Unsequenced
	AATU01016121	67->1005	98%	3' truncated
	AATU01010944	>940-<7	98%	Unsequenced
	AATU01001279	978-1721	99%	Unsequenced
Utopia-2_PI	AATU01001283	12190-13470	100%	U2 (14921-14985)
	AATU01001283	5305-6585	99%	U2 (8036-8100)
	AATU01001299	3142-1862	99%	U2 (416-352)
	AATU01000164	3567-4847	98%	U2 (6292-6356)
	AATU01000141	1211-2495	97%	U2 (3920-3984)
	AATU01000170	257->1230	98%	Unsequenced
Utopia-3_PI	AATU01001281	8953-10233	100%	U2 (11703-11767)
	AATU01001283	19194-20474	99%	U2 (21947-22011)
	AATU01001290	24521-23241	99%	U2 (21770-21706)
	AATU01001290	31558-30279	99%	U2 (28829-28765)
	AATU01000157	842-2120	99%	3' truncated
	AATU01000157	3282->3934	99%	Unsequenced
	AATU01001292	1851-571	99%	Unsequenced
	AATU01000167	1020-2300	99%	Unsequenced
	AATU01001293	>2402-1225	99%	3' truncated
	AATU01001287	9322-8151	97%	3' truncated
	AATU01004087	<2688-3392	99%	Unsequenced
Utopia-4_PI	AATU01001281	15572-16855	100%	U2 (18264-18328)
	AATU01001281	28625-29908	99%	Unsequenced
	AATU01001282	1036-2320	98%	Unsequenced

	AATU01001287	3775-2487	98%	U2 (1076-1012)
	AATU01001298	2425-1137	98%	Unsequenced
	AATU01000137	3484-4769	97%	U2 (6153-6217)
	AATU01000138	6150-7434	96%	U2 (8818-8882)
	AATU01000138	2804-4094	96%	U2 (5507-5569)
	AATU01000138	9460->10339	98%	Unsequenced
	AATU01000138	<48-746	96%	U2 (2161-2223)
	AATU01000151	<1756-2984	95%	Unsequenced
	AATU01018263	>706-<1	98%	Unsequenced
	AATU01001295	>10810-10199	99%	U2 (8788-8726)
	AATU01009644	>1956-<1743	96%	3’ truncated
Utopia-5_PI	AATU01010946	3176-1896	100%	U2 (457-393)
	AATU01000193	>228427-227151	98%	3’ truncated

1 Identity to the first copy of each *Utopia* family.
