# Supplementary material for: Ancient Origin of the U2 Small Nuclear RNA Gene-Targeting Non-LTR Retrotransposons Utopia
Source: PLoS One. 2015 Nov 10;10(11):e0140084. doi: 10.1371/journal.pone.0140084 (PMC4640811; doi:10.1371/journal.pone.0140084)
Supplement: S3 Table — (PDF) [file pone.0140084.s009.pdf]

**S3 Table.** All RT-coding sequences of *Utopia* elements in *P. sojae*.

| Family | Scaffold | Position of RT | Identity[1] | 3’ Flanking seq. |
|---|---|---|---|---|
| *Utopia-1_PS* | 25 | 782643-783926 | 100% | Unsequenced |
| | 25 | <767931-769023 | 97% | U2 fragment[2] |
| | 72 | 337441-336158 | 97% | U2 (334751-334684) |
| | 72 | 329386-328111 | 97% | U2 (326767-326700) |
| | 36 | 1058->1499 | 99% | U2 (2244-2311) |
| | 36 | <2774-3786 | 99% | U2 (5193-5260) |
| | 892 | 7207-<6570 | 98% | Unsequenced |
| | 1116 | 228->883 | 97% | Unsequenced |
| *Utopia-2_PS* | 25 | 758495-759775 | 100% | U2 (761325-761392) |
| | 1893 | 987-2264 | 98% | Unsequenced |
| | 784 | 7830-<6601 | 98% | Unsequenced |
| | 72 | >331966-330804 | 98% | U2 (330075-330008) 3’ truncated[3] |
| | 72 | 343570-<342648 | 99% | Unsequenced |
| | 72 | <333501-334220 | 99% | Unsequenced |
| | 1191 | >4846-3833 | 98% | Unsequenced |
| | 370 | 7138-6007 | 99% | U2 (5280-5215) 3’ truncated[3] |
| | 1678 | 3106->3564 | 99% | Unsequenced |

1 Identity to the first copy of each *Utopia* family.

2 TCTGTTCTAATCAGTGTGAAA (770204-770224).

3 The 3’ truncated *Utopia-2_PS* copy is followed by a 5’ truncated *Utopia-1_PS* copy.
