# Supplementary material for: Ancient Origin of the U2 Small Nuclear RNA Gene-Targeting Non-LTR Retrotransposons Utopia
Source: PLoS One. 2015 Nov 10;10(11):e0140084. doi: 10.1371/journal.pone.0140084 (PMC4640811; doi:10.1371/journal.pone.0140084)
Supplement: S4 Table — (PDF) [file pone.0140084.s010.pdf]

S4 Table. All RT-coding sequences of *Utopia* elements in *P. ramorum*.

**Family**	**Scaffold**	**Position of RT**	**Identity[1]**	**3' Flanking seq.**
Utopia-1_PR	271	16238-14955	100%	U2 (13548-13481)
	76	42741-41458	99%	U2 (40051-39984)
Utopia-2_PR	1303	4133-5413	100%	Unsequenced
	271	9250-8052	97%	U2 (6559-6492)
	597	7307-6265	99%	3' truncated
	1960	589-<1	98%	Unsequenced
Utopia-3_PR	271	22521-21246	100%	U2 (19550-19483)
	76	35822-34617	99%	U2 (33111-33044)
	323	2628-1591	99%	U2 (67-1)
Utopia-4_PR	1494	3349-2069	100%	U2 (543-476)
	12	636501-637780	99%	Unsequenced
	196	39599->40536	100%	U2 (42601-42668)
	76	>60992-60276	99%	U2 (58827-58760)
Utopia-5_PR	76	25019-23739	100%	U2 (26878-26811)
	2014	<5-1030	97%	Unsequenced
	323	12537-<11604	99%	Unsequenced

1 Identity to the first copy of each *Utopia* family.
